# Palladium-catalyzed benzylic C(sp^3^)–H carbonylative arylation of azaarylmethyl amines with aryl bromides†

**DOI:** 10.1039/d1sc02078a

**Published:** 2021-07-08

**Authors:** Haoqiang Zhao, Bowen Hu, Lijin Xu, Patrick J. Walsh

**Affiliations:** Roy and Diana Vagelos Laboratories, Penn/Merck Laboratory for High-Throughput Experimentation, Department of Chemistry, University of Pennsylvania 231 South 34th Street Philadelphia Pennsylvania 19104-6323 USA pwalsh@sas.upenn.edu; Department of Chemistry, Renmin University of China Beijing 100872 China 20050062@ruc.edu.cn

## Abstract

A highly selective palladium-catalyzed carbonylative arylation of weakly acidic benzylic C(sp^3^)–H bonds of azaarylmethylamines with aryl bromides under 1 atm of CO gas has been achieved. This work represents the first examples of use of such weakly acidic pronucleophiles in this class of transformations. In the presence of a NIXANTPHOS-based palladium catalyst, this one-pot cascade process allows a range of azaarylmethylamines containing pyridyl, quinolinyl and pyrimidyl moieties and acyclic and cyclic amines to undergo efficient reactions with aryl bromides and CO to provide α-amino aryl-azaarylmethyl ketones in moderate to high yields with a broad substrate scope and good tolerance of functional groups. This reaction proceeds *via in situ* reversible deprotonation of the benzylic C–H bonds to give the active carbanions, thereby avoiding prefunctionalized organometallic reagents and generation of additional waste. Importantly, the operational simplicity, scalability and diversity of the products highlight the potential applicability of this protocol.

## Abstract 2

Introduced is a method for the deprotonative carbonylation of azaarylmethyl amines with aryl bromides. The reaction employs a Pd(NIXANTPHOS)-based catalyst and takes place under 1 atm CO.

## Graphical abstract

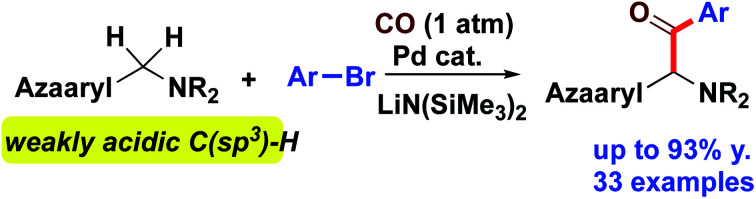

## Introduction

**Fig. 1:**
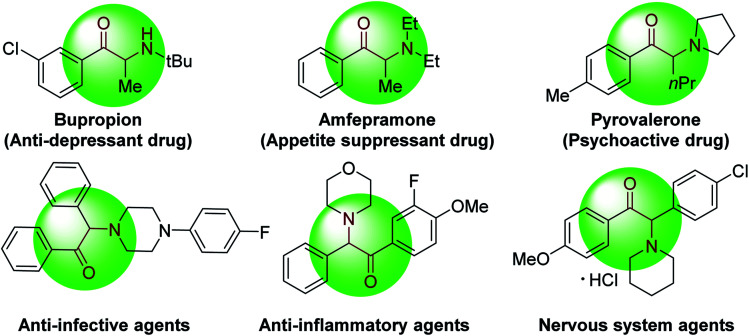
Selected pharmacologically active compounds containing α-amino aryl ketones.

α-Amino ketones are key components of numerous biologically active natural products and synthetic compounds. They display a wide range of medicinal and biological activities, such as anti-depressant, appetite suppressant, and anti-platelet properties (Fig. 1).^1^ α-Amino ketones also serve as effective synthetic intermediates for the preparation of various heterocycles and 1,2-amino alcohols.^1*b*,2^ As a result of their widespread utility, there has been substantial and long-standing interest in the efficient construction of α-amino ketones in the synthetic community.^3^ General approaches to construct α-amino ketones from ketones or their derivatives involve nucleophilic amination,^4^ electrophilic amination^5^ or oxidative amination.^6^ Considerable efforts have also been made to synthesize α-amino ketones *via* acylation of imines with aldehydes, acylsilanes or carboxylic acids,^7^ Stille reaction of sulfonamides with benzoyl chlorides,^8^ cross-coupling of thiol esters with boronic acids or organostannanes,^9^ hydrogenation of α-dehydroamino ketones or α-ketoketimines,^10^ and rearrangement of α-hydroxyl imines or enamines.^11^ Moreover, recent years have witnessed the preparation of α-amino ketones starting from alkenes,^12^ alkynes^13^ and sulfonium ylides.^14^ Despite these promising advances, exploration of straightforward methods that enable formation of multiple C–C bonds remain appealing.

**Scheme 1:**
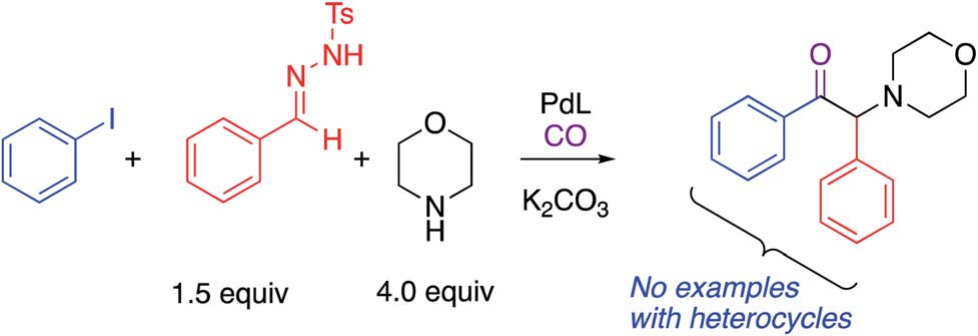
Carbene-based approach of Wang and co-workers.

Due to its low cost, high reactivity and abundance, CO has been extensively explored as a versatile C1 building block for the production of carbonyl-containing compounds and heterocycles.^15^ Impressive achievements have been recorded in transition-metal catalyzed reactions of CO in multicomponent carbonylation reactions to construct carbonyl-containing molecules from simple starting materials.^15*d*,16^ Little attention, however, has been paid to the application of this strategy for the preparation of synthetically valuable α-amino ketones. There is only one such report in the literature. In 2018, Wang, Zhang and co-workers described an elegant synthesis of α-amino ketones *via* a Pd(0)-catalyzed four-component carbonylation reaction of aryl iodides, *N*-tosylhydrazones and amines under 1 atm of CO (Scheme 1).^17^ Despite the synthetic potential of this 4 component coupling, this protocol is only applicable to aryl iodides and no examples with heteroaryl groups were reported.

**Scheme 2:**
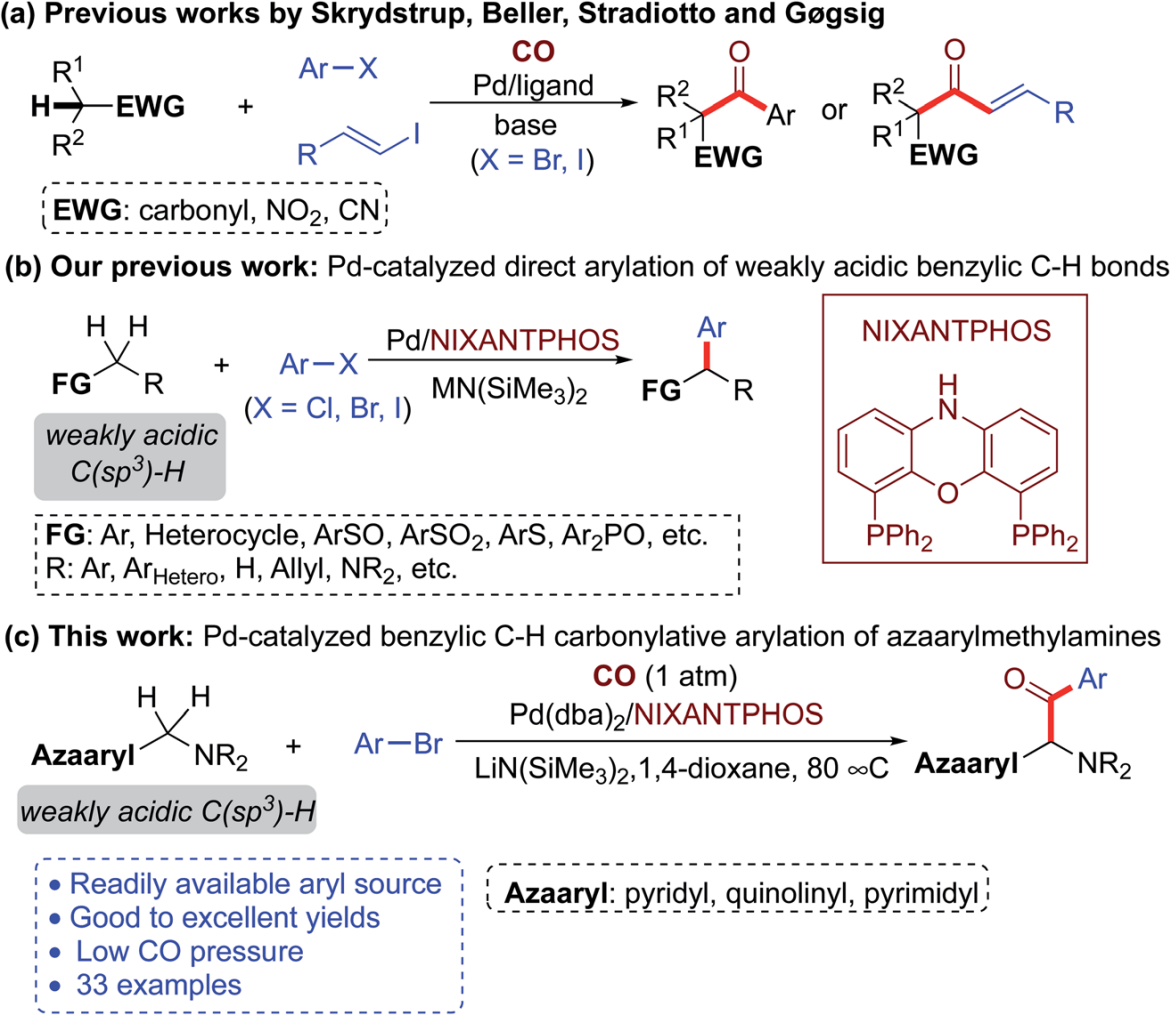
Deprotonative carbonylative cross-coupling reactions (a) Prior art with more acidic pro-nucleophiles, (b) Our prior work on the direct coupling reaction in the absence of CO, (c) The current study using weakly acidic Azaarylmethylamine pro-nucleophiles.

Recent progress has established the viability of palladium-catalyzed carbonylative cross-coupling reactions of acidic C(sp^3^)–H bonds for the concurrent formation of two new C–C bonds with the introduction of a carbonyl group.^18,19^ Early reports focused on the arylation of activated C(sp^3^)–H bonds of malonate derivatives.^18^ In 2012, Skrydstrup and co-workers first realized carbonylative α-arylation of ketones with aryl iodides using CO in the presence of a catalytic amount of [Pd(dba)_2_] and a bidentate phosphine ligand to afford 1,3-diketones (Scheme 2a).^19*a*^ Subsequently, the same group accomplished carbonylative α-arylation of monoester potassium malonate,^19*b*^ acetylacetones,^19*d*^ ketones,^19*e*^ 2-oxindoles,^19*g*^ nitromethanes,^19*h*^ substituted 1,3-dioxin-4-ones,^19*i*^ and cyanoacetates^19*j*^ with aryl iodides and aryl bromides under palladium catalysis. Meanwhile, Beller and co-workers described the Pd-catalyzed carbonylative α-arylation of ketones and nitriles with aryl iodides and pressurized CO gas (Scheme 2a).^19*c*,*f*^ These studies take advantage of strongly activated C(sp^3^)–H bonds to facilitate deprotonation. The carbonylative α-arylation of weakly acidic C(sp^3^)–H bonds remains underdeveloped, despite the potential utility of such a method with a wide variety of pronucleophiles.

In recent years, our team has built a program around direct arylation of weakly acidic C(sp^3^)–H bonds (p*K*_a_ 25–43 in DMSO)^20^ with aryl halides by employing palladium catalysts and suitable bases (Scheme 2b). We called these reactions deprotonative cross-coupling processes (DCCP).^21^ Based on these studies from our lab, we envisioned that merging DCCP with carbonylation reactions would enable preparation of a host of new ketones. Herein we describe such a new and efficient process that allows highly selective carbonylative arylation of weakly acidic benzylic C(sp^3^)–H bonds of azaarylmethylamines with aryl bromides. These reactions are conveniently conducted with 1 atm of CO and a palladium catalyst to deliver α-amino aryl-azaarylmethyl-ketone products in good to excellent yields. The reaction has a broad substrate scope and good tolerance of functional groups (Scheme 2c).

## Results and discussion

We started our studies by exploring the reaction conditions of benzylic C–H carbonylative arylation of 4-(pyridin-2-ylmethyl)morpholine (**1a**) with 1-bromo-4-(*tert*-butyl)benzene (**2a**) under 1 atm of CO gas (Table 1). Considering that we have recently achieved the coupling of azaarylmethyl amines with aryl halides to generate aryl(azaaryl)methyl amines in 1,4-dioxane using a Pd(OAc)_2_/NIXANTPHOS-based catalyst together with LiN(SiMe_3_)_2_ as the base,^21*c*^ we first assessed the feasibility of the proposed reaction of **1a**, **2a** and CO (1 atm) at 65 °C for 16 h by employing the same catalytic system. To our delight, the carbonylative arylation reaction indeed occurred, delivering the expected product **3aa** in 27% AY (AY = assay yield, determined by ^1^H NMR spectroscopy) with the formation of the non-carbonylative coupling product (also called the direct coupling product) **3aa′** in 8% AY (Table 1, entry 1 and ESI, Table S2†).

**Table 1:** Optimization of reaction conditions for benzylic C–H carbonylative arylation of **1a** with **2a**^a^

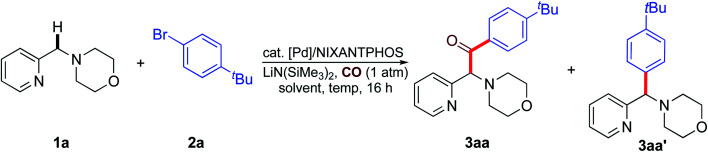				
Entry	Pd source	Solvent	Temp (°C)	**3aa** Assay yield^b^ (%)
1	Pd(OAc)_2_	1,4-Dioxane	65	27
2	Pd(OAc)_2_	THF	65	22
3	Pd(OAc)_2_	CPME	65	21
4	Pd(OAc)_2_	DME	65	10
5	Pd_2_(dba)_3_	1,4-Dioxane	65	31
6	Pd G4 dimer	1,4-Dioxane	65	37
7	[PdCl(allyl)]_2_	1,4-Dioxane	65	7
8	Pd(dba)_2_	1,4-Dioxane	65	43
9^c^	Pd(dba)_2_	1,4-Dioxane	65	89
10^c^	Pd(dba)_2_	1,4-Dioxane	80	93
11^c^	Pd(dba)_2_	1,4-Dioxane	100	89
**12** c ^,^ d	**Pd(dba)**_**2**_	**1,4-Dioxane**	**80**	**97 (92)** e
13^c^^,^^d^^,^^f^	Pd(dba)_2_	1,4-Dioxane	80	0
14^c^^,^^d^	—	1,4-Dioxane	80	0
15^c^^,^^d^^,^^g^	Pd(dba)_2_	1,4-Dioxane	80	4

a Reaction conditions: **1a** (0.1 mmol), **2a** (0.12 mmol), LiN(SiMe_3_)_2_ (2 equiv.), [Pd] (5 mol%), NIXANTPHOS (6 mol%), solvent (1 mL).

b Yields were determined by ^1^H NMR analysis of unpurified reaction mixtures with internal standard CH_2_Br_2_.

c LiN(SiMe_3_)_2_ (3 equiv.) was employed.

d **2a** (1.5 equiv.) was employed.

e Isolated yield.

f In the absence of NIXANTPHOS.

g CO (8.6 atm) was applied. Pd G4 dimer: Buchwald G4 precatalysts; dba: dibenzylideneacetone.

With an aim to improve the reaction efficiency, the performance of the coupling in other solvents, such as THF, CPME (cyclopentyl methyl ether) and DME were examined (Table 1, entries 2–4), but none outperform 1,4-dioxane. Replacement of LiN(SiMe_3_)_2_ with NaN(SiMe_3_)_2_, or KN(SiMe_3_)_2_ failed to give better results, and the reaction was not promoted with LiO^*t*^Bu, NaO^*t*^Bu, or KO^*t*^Bu as the base (ESI, Table S2†). The subsequent screening of palladium salts revealed the superiority of Pd(dba)_2_ in this reaction, allowing the generation of product **3aa** in 43% yield (Table 1, entry 8). Unexpectedly, increasing the loading of LiN(SiMe_3_)_2_ to 3 equiv. improved the yield of **3aa** to 89% with only a small amount of direct coupling byproduct **3aa′** (7%) (Table 1, entry 9 and ESI, Table S2†). The yield of **3aa** could be further enhanced to 93% with an increase of reaction temperature to 80 °C (Table 1, entry 10), but a higher reaction temperature of 100 °C was found to be detrimental (Table 1, entry 11). Further examination of the stoichiometry indicated that increasing the amount of **2a** from 1.2 to 1.5 equiv. resulted in almost exclusive formation of **3aa** in an excellent assay yield of 97% with 92% isolated yield (Table 1, entry 12). The phosphine ligand bound to palladium also proved to be critical. Variation of the bidentate phosphine ligand to dppe, dppb, dppp, dppf and xantphos all led to substantial decreases in the reaction conversion (ESI, Table S2†). Control experiments confirmed the dependency on both the phosphine ligand and the palladium source in this transformation (Table 1, entries 13 and 14). Increasing the CO pressure to 8.6 atm resulted in only 4% AY (Table 1, entry 15), which suggested a higher CO pressure could potentially saturate the metal catalyst and deactivate it.

With the optimized reaction conditions in hand, we then evaluated the substrate scope of azaarylmethylamines and the results are summarized in Table 2. 2-Pyridylmethylamines (**1b–1f**) bearing thiomorpholine, methylpiperazine, dimethylamine, pyrrolidine and piperidine underwent smooth C–H carbonylative arylation to afford the expected α-amino ketone products (**3ba–3fa**) in 64–87% yield. Substrate **1g** bearing a sterically hindered *ortho*-substituent on the pyridine ring was also reactive, delivering the desired product **3ga** in 52% yield. The more acidic 4-pyridylmethylamines (**1h–1k**) containing different amino groups reacted efficiently to give the desired products (**3ah–3ak**) in 61–88% yield. In the case of 3-pyridylmethylamine, however, the reaction failed to yield any desired product, with recovery of most of the starting materials. It is interesting to note that in the that direct coupling with the 3-pyridylmethylamine (in the absence of CO), the reaction was successful with KN(SiMe_3_)_2_ as base.^21*c*^ Use of KN(SiMe_3_)_2_ instead of LiN(SiMe_3_)_2_ under CO at 110 °C, however, did not lead to ketone product.^21*c*^ It is likely that the higher p*K*_a_ of this pronucleophile, in combination with the less reactive base LiN(SiMe_3_)_2_ disfavors formation of sufficient quantities of the nucleophile and prevents the reaction from proceeding.^20^ This result shows the crucial dependency on the main group metal in such multistep reactions. To further extend the scope of this carbonylative arylation, other azaarylmethylamines were tested. Notably, when 4-(benzo[*d*]thiazol-2-ylmethyl)morpholine **1l** was employed, the product (**3la**) was obtained in 50% yield as a mixture of the ketone and enol isomers (the ratio was ketone : enol = 3 : 1). However, for other kinds of azaarylmethylamines like 2-(morpholinomethyl)benzo[*d*]oxazole, 4-((1-methyl-1*H*-imidazol-2-yl)methyl)morpholine, 4-benzylmorpholine or even 2-(ethoxymethyl)pyridine, the reaction failed to yield the desired products (See ESI, Table S3† for substrates that were unsuccessful with this catalyst system). When 2-quinolinylmethyl amines **1m** and **1n** were employed, the corresponding products **3ma** and **3na** were obtained in 74% and 72% yields, respectively. Moreover, 2-pyrimidylmethylamine **1o** also reacted smoothly, affording product **3oa** in 93% yield.

**Table 2:** Pd-catalyzed benzylic C–H carbonylative arylation of azaarylmethylamines **1** with **2a**^a^^,^^b^

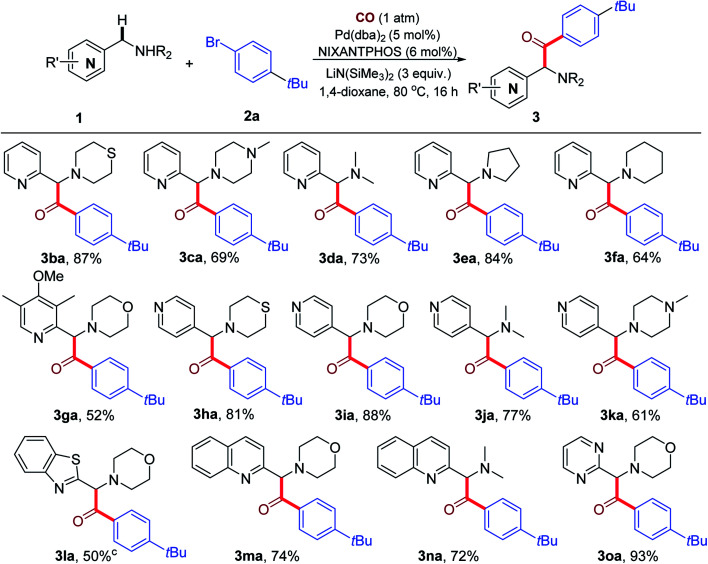

a Reaction conditions: **1** (0.2 mmol), **2a** (0.3 mmol), Pd(dba)_2_ (5 mol%), NIXANTPHOS (6 mol%), LiN(SiMe_3_)_2_ (3.0 equiv.), 1,4-dioxane (2.0 mL), 80 °C, 16 h, under CO atmosphere (1 atm).

b Isolated yields.

c A mixture of ketone and enol forms were obtained (ketone : enol = 3 : 1).

We next investigated the reaction of various aryl bromides with **1a** under 1 atm CO (Table 3). A variety of *para*-substituted aryl bromides bearing electron-donating groups (**2b–2f**, **2j** and **2k**) and electron-withdrawing groups (**2g–2i**) were all effective reaction partners, providing the corresponding products (**3ab–3ak**) in moderate to high yields (57–90%). Moreover, the reaction could be successfully extended to *meta*-substituted aryl bromides (**2l–2n**), delivering the desired products (**3al–3an**) in 61–84% yields. Lower yields were generally observed with electron-poor aryl bromides, possibly due to the partial decomposition of these aryl bromides in the presence of the base. Notably, 1-bromo-4-chlorobenzene (**2h**) and 1-bromo-3-chlorobenzene (**2m**) led to the formation of products **3ah** and **3am** in 77% and 71% yields, respectively, with the chloro group remaining intact during the reaction process. The Pd(NIXANTPHOS)-based catalysts is known to oxidatively add aryl chlorides at room temperature (see ESI, Table S2†).^21^ In the presence of CO, however, aryl chlorides were not reactive. These observations suggest to us that the palladium catalyst bears a CO ligand that tempers its ability to oxidatively add the stronger C–Cl bond of aryl chlorides.

**Table 3:** Pd-catalyzed benzylic C–H carbonylative arylation of **1a** with aryl bromides^a^^,^^b^

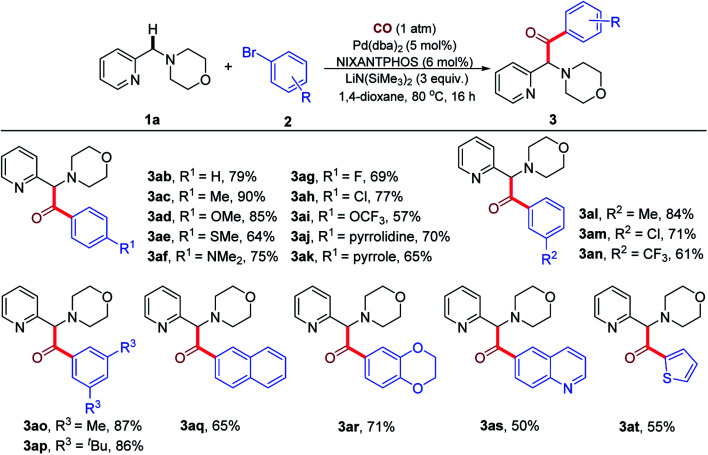

a Reaction conditions: **1a** (0.2 mmol), **2** (0.3 mmol), Pd(dba)_2_ (5 mol%), NIXANTPHOS (6 mol%), LiN(SiMe_3_)_2_ (3.0 equiv.), 1,4-dioxane (2.0 mL), 80 °C, 16 h, under CO atmosphere (1 atm).

b Isolated yields.

Multi-substituted aryl bromides (**2o–2r**) also readily engaged in the transformation to afford the corresponding products (**3ao–3ar**) in 65–87% yields. The generality of the current catalytic system was further demonstrated by the success of heteroaryl bromides **2s** and **2t** to generate **3as** and **3at** in 50% and 55% yields, respectively. It is noteworthy that the products of these reactions are rich in heterocycles.

**Scheme 3:**
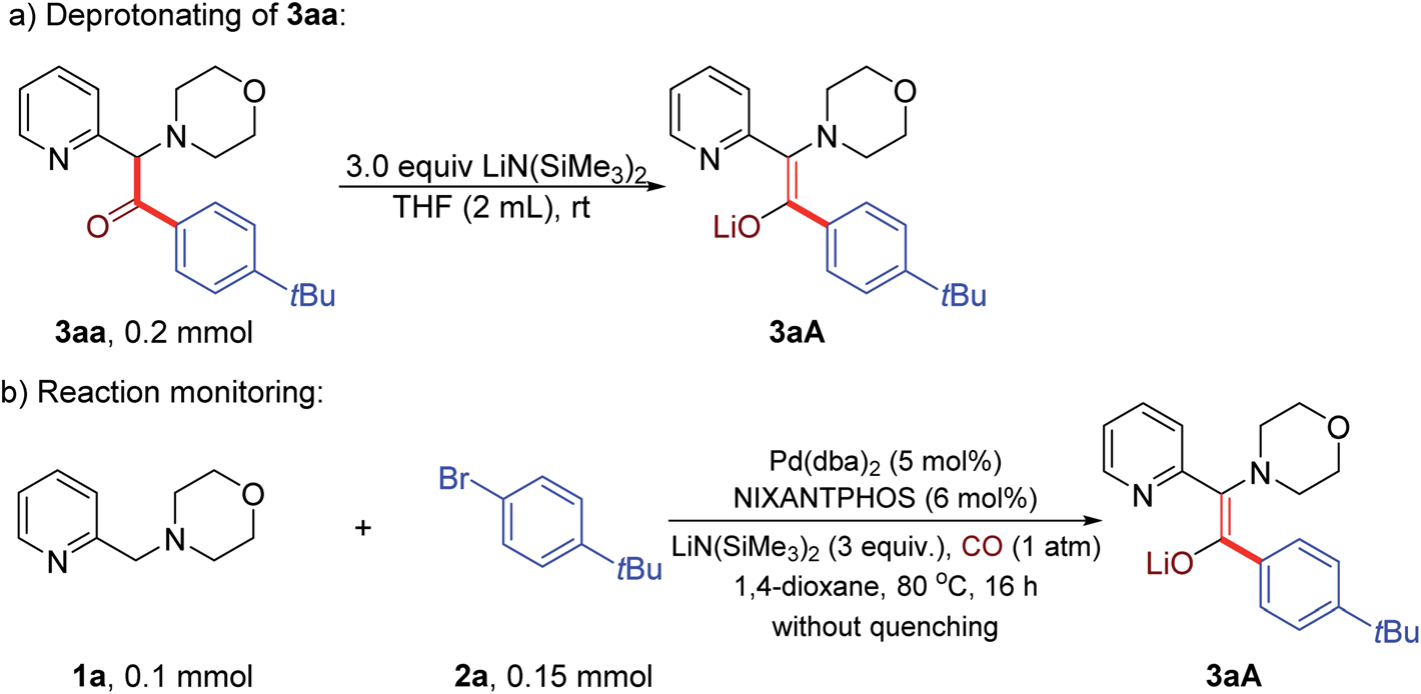
Detection of product precursor (a) Independent synthesis of enolate **3aA**, (b) Determination of the reaction product before workup.

Preliminary studies were conducted to gain insight into the reaction product. We hypothesized that the product generated under the reaction conditions before workup was not the ketone, but the enolate that is protonated upon aqueous workup. Thus, we first prepared the enolate **3aA** by deprotonation of ketone **3aa** with LiN(SiMe_3_)_2_ (Scheme 3a). Next, the carbonylative α-arylation reaction of **1a**, **2a** and CO was conducted and the product characterized by NMR spectroscopy before quenching with water (Scheme 3b). This product was found to be identical to the independently synthesized enolate **3aA**, confirming our hypothesis that the enolate is the product of the reaction (Scheme 3). This result also helps to explain why 3 equiv. of LiN(SiMe_3_)_2_ are optimal. An equivalent is needed to consume the starting material **1** and a second to deprotonate the ketone. The role of the third is to deprotonate the catalyst backbone N–H and to react with any advantageous water.

**Scheme 4:**
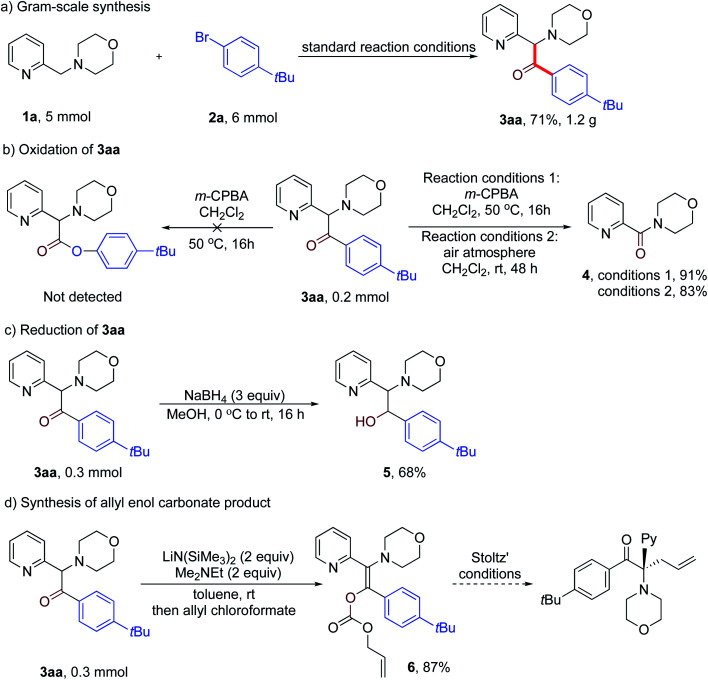
Synthetic applications (a) scale-up of reaction, (b) product oxidation, (c and d) further transformations of the product.

To showcase the synthetic utility of our method, we first conducted a gram-scale reaction of **1a**, **2a** and CO under the standard reaction conditions. The desired product **3aa** was obtained in 71% yield (1.2 g) (Scheme 4a). Next, we attempted the transformation of the product ketones into other useful derivatives. Although Baeyer–Villiger oxidation of **3aa** with *m*-CPBA in CH_2_Cl_2_ failed to give the desired ester, an unexpected oxidative cleavage reaction occurred to give product **4** in 91% yield (Scheme 4b). Notably, further study revealed that this oxidative cleavage reaction could take place under air oxidation to give a high yield of **4** (83%). Moreover, reduction of **3aa** with NaBH_4_ in MeOH at room temperature for 16 h resulted in the formation of amino alcohol product **5** in 68% yield (Scheme 4c). Finally, treatment of **3aa** with LiN(SiMe_3_)_2_ and Me_2_NEt followed by addition of allyl chloroformate gave the allyl enol carbonate product **6** in 87% isolated yield as a single diastereomer (Scheme 4d). As reported by the Stoltz group,^22^ this enol carbonate product can undergo palladium-catalyzed enantioselective decarboxylative allylic alkylation to afford a chiral α-quaternary ketone.

**Scheme 5:**
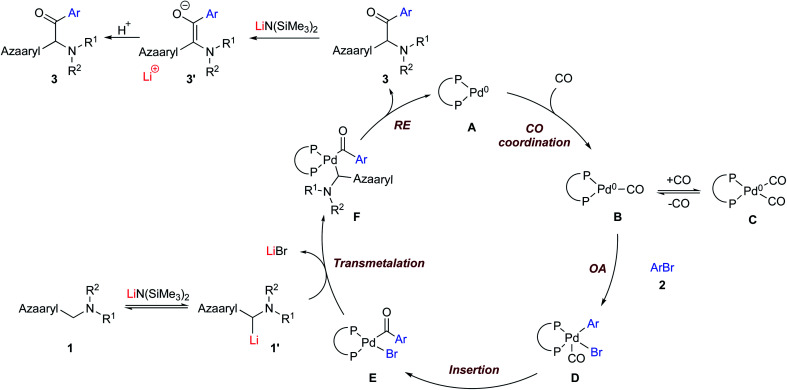
Plausible mechanism.

Based on the aforementioned results in DCCP chemistry (no CO)^21^ and previous reports on carbonylative arylation,^19,23^ a plausible mechanism is proposed in Scheme 5. The catalytic cycle starts with complexation of NIXANTPHOS (see Scheme 2b for structure) and Pd(dba)_2_ to yield the (NIXANTPHOS)Pd(0) species **A**.^21*a*^ We previously demonstrated that the NIXANTPHOS N–H (p*K*_a_ 21 in DMSO) will be deprotonated by silyl amide bases to afford bimetallic adducts that have exhibited cooperativity between the Pd and main group cation.^21*m*^ In the absence of CO, the resulting heterobimetallic complex will oxidatively add aryl chlorides at room temperature through a mechanism involving cooperativity between the main group metal and the palladium.^21*m*^ In the presence of CO we also expect the ligand N–H to be deprotonated by LiN(SiMe_3_)_2_. The oxidative addition does not appear to be accelerated and the carbonylation reaction does not work with aryl chlorides. Under CO atmosphere, the Pd(0) species is proposed to undergo CO coordination to generate Pd(0) carbonyl (**B**) and dicarbonyl **C** complexes.^23*b*^ This proposal is consistent with inhibition at high CO pressures. Upon dissociation of CO from **C** to generate the 16 electron mono-carbonyl adduct **B**, oxidative addition of the aryl bromide **2** takes place to produce (NIXANTPHOS)Pd(CO)(Ar)Br-complex **D**. Intermediate **D** likely undergoes CO insertion into the Pd–Ar bond to furnish the acyl–Pd(ii) complex **E**. Intermediate **E** reacts with the deprotonated pronucleophile **1′** in a transmetallation step to deliver the reductive elimination precursor **F**. Reductive elimination of **F** gives the ketone product **3** and Pd(0) species **A** to close the catalytic cycle. In the presence of LiN(SiMe_3_)_2_ the ketone **3** is rapidly deprotonated, furnishing the enolate **3′**. Quenching the reaction with H_2_O results in the formation of the observed ketone product **3**. The direct coupling product likely forms when transmetallation takes place before CO insertion or if CO insertion is reversible. At this stage, we cannot rule out the possibility of an adduct between Pd(0) and the enolate, as described by Skrydstrup and coworkers.^19*e*^ Further investigations into the reaction mechanism will be presented in due course.

## Conclusion

In conclusion, we have developed the carbonylative arylation of weakly acidic benzylic C(sp^3^)–H bonds of azaarylmethylamines with aryl bromides and CO using a Pd catalyst. This work is unique in that it employs pronucleophiles with high p*K*_a_ values, suggesting a wide variety of previously overlooked substrates may be viable coupling partners in carbonylation reactions. The reaction is operative under 1 atm of CO and does not require high pressure equipment. This one-pot cascade process is applicable to the coupling of a wide range of azaarylmethylamines and aryl bromides, enabling facile access to useful α-amino aryl-azaarylmethyl-ketones in moderate to high yields with good functional group tolerance. This work provides an attractive and complementary approach to prepare heteroatom-rich α-amino ketones.

## Data availability

The datasets supporting this article have been uploaded as part of the ESI.

## Author contributions

HZ performed the optimization of the reaction with help from BH. The substrate scope and product characterization was performed by HZ and BH. The first draft was written by HZ and all authors contributed to revising the draft. The project was conceived by PJW and the research directed by PJW and LX.

## Conflicts of interest

The authors declare no competing financial interest.

## Supplementary Material

SC-012-D1SC02078A-s001
