# Supplementary material for: Palladium-catalyzed benzylic C(sp3)–H carbonylative arylation of azaarylmethyl amines with aryl bromides
Source: Chem Sci. 2021 Jul 8;12(32):10862–70. doi: 10.1039/d1sc02078a (PMC8372623; doi:10.1039/d1sc02078a)
Supplement: SC-012-D1SC02078A-s001 [file SC-012-D1SC02078A-s001.pdf]

Electronic Supplementary Material (ESI) for Chemical Science.
This journal is © The Royal Society of Chemistry 2021

Supporting Information for

Palladium-Catalyzed Benzylic C(sp^3)–H Carbonylative Arylation of Azaarylmethyl Amines with Aryl Bromides Under CO at Atmospheric Pressure

Haoqiang Zhao,[a,b,‡] Bowen Hu,[a,‡] Lijin Xu[b,]* and Patrick J. Walsh[a,]*

[a.]Roy and Diana Vagelos Laboratories, Penn/Merck Laboratory for High-Throughput Experimentation, Department of Chemistry, University of Pennsylvania, 231 South 34th Street, Philadelphia, Pennsylvania 19104-6323, United States. E-mail: pwalsh@sas.upenn.edu
[b.]Department of Chemistry, Renmin University of China, Beijing 100872, China. E-mail: 20050062@ruc.edu.cn

Contents:

1. General information: S2
2. Optimization of the reaction conditions S2
3. General Procedure for DCCC of Azaarylmethyl Amines S5
4. The general procedure for synthetic applications S6
5. Reaction studies S9
6. Characterization data for products S11
7. References: S19
8. Copies of ^{1}H and ^{13}C{^{1}H} NMR Spectra S20

1. General information:

Unless otherwise noted, all experiments were carried out in air and all commercially available chemicals, including organic solvents, were used as received from Aldrich, Acros or Strem without further purification. ^{1}H NMR and ^{13}C{^{1}H} NMR spectra were recorded on a Bruker Model Advance DMX 400 Spectrometer (^{1}H 400 MHz and ^{13}C 101 MHz, respectively) or Bruker Model Advance DMX 500 Spectrometer (^{1}H 500 MHz and ^{13}C 125 MHz, respectively). Chemical shifts (δ) are given in ppm and are referenced to residual solvent peaks. Melting points were measured on X-4 melting point apparatus and are uncorrected. High resolution mass spectra (HRMS) were performed on a VG Autospec-3000 spectrometer. Column chromatography was performed with silica gel (200-300 mesh). Azaarylmethyl Amines were prepared according to the previous reports.[1,2]

2. Optimization of the reaction conditions

a) HTE (High Throughput Experimentation) micro-scale (0.01 mmol) screen

Table S1. Base and solvent screening.

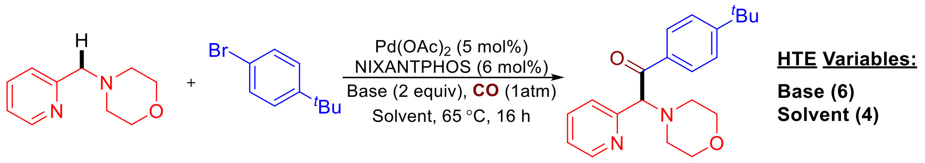

1a, 1 equiv **2a,** 1.2 equiv **3aa**

6 Base: Li*Ot*Bu, Na*Ot*Bu, K*Ot*Bu, LiN(SiMe$_3$)$_2$, NaN(SiMe$_3$)$_2$, KN(SiMe$_3$)$_2$.

4 Solvent: Toluene, 1,4-dioxane, CMPE (cyclopentyl methyl ether), THF.

2:1 ratio relative to **1a** for base and 1.2:1 ratio for aryl bromide **2a**.

Entry	Base	Solvent	AY (%)
1	LiO*t*Bu	Toluene	0
2	NaO*t*Bu	Toluene	0
3	KO*t*Bu	Toluene	0
4	LiN(SiMe$_3$)$_2$	Toluene	9
5	NaN(SiMe$_3$)$_2$	Toluene	4
6	KN(SiMe$_3$)$_2$	Toluene	2
7	LiO*t*Bu	1,4-dioxane	0
8	NaO*t*Bu	1,4-dioxane	0
9	KO*t*Bu	1,4-dioxane	0
10	LiN(SiMe$_3$)$_2$	1,4-dioxane	22
11	NaN(SiMe$_3$)$_2$	1,4-dioxane	6
12	KN(SiMe$_3$)$_2$	1,4-dioxane	3
13	LiO*t*Bu	CPME	0
14	NaO*t*Bu	CPME	0
15	KO*t*Bu	CPME	0
16	LiN(SiMe$_3$)$_2$	CPME	4

17	$NaN(SiMe_3)_2$	CPME	0
18	$KN(SiMe_3)_2$	CPME	0
19	Li*Ot*Bu	THF	0
20	Na*Ot*Bu	THF	0
21	K*Ot*Bu	THF	0
22	$LiN(SiMe_3)_2$	THF	7
23	$NaN(SiMe_3)_2$	THF	0
24	$KN(SiMe_3)_2$	THF	0

The lead hit from the screening was the combination of $Pd(OAc)_2$ (5 mol %), NIXANTPHOS (6 mol %), $LiN(SiMe_3)_2$ (2 equiv), 1,4-dioxane as solvent under 1 atm CO at 65 °C for 16 h giving 22% assay yield of the desired carbonylation product **3aa**. A scale-up reaction on a 0.1 mmol scale using General Procedure for the Pd-Catalyzed Deprotonative Carbonylation of **1a** proved successful with 27% assay yield of **3aa** determined by ^{1}H NMR spectroscopy of the crude reaction mixture.

b) Lab scale (0.1 mmol) reaction conditions optimization

Table S2. Optimization of reaction conditions.[a]

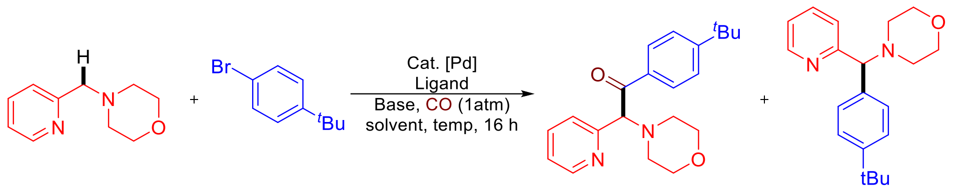

Entry	Pd source	Ligand	Solvent	Temp (°C)	1a:2a:base	Assay yield (%)[b]	
						3aa	3aa'
1	$Pd(OAc)_2$	NIXANTPHOS	Toluene	65	1:1.2:2	18	5
2	$Pd(OAc)_2$	NIXANTPHOS	DMSO	65	1:1.2:2	0	0
3	$Pd(OAc)_2$	NIXANTPHOS	THF	65	1:1.2:2	22	4
4	$Pd(OAc)_2$	NIXANTPHOS	CPME	65	1:1.2:2	21	9
5	$Pd(OAc)_2$	NIXANTPHOS	DME	65	1:1.2:2	10	6
6	$Pd(OAc)_2$	NIXANTPHOS	1,4-dioxane	65	1:1.2:2	27	8
7	$Pd_2(dba)_3$	NIXANTPHOS	1,4-dioxane	65	1:1.2:2	31	11
8	$Pd(PPh_3)_4$	NIXANTPHOS	1,4-dioxane	65	1:1.2:2	22	trace
9	$[PdCl(allyl)]_2$	NIXANTPHOS	1,4-dioxane	65	1:1.2:2	7	5
10	Pd G3 dimer	NIXANTPHOS	1,4-dioxane	65	1:1.2:2	32	trace
11	$Pd(dba)_2$	NIXANTPHOS	1,4-dioxane	65	1:1.2:2	43	10
12	Pd G4 dimer	NIXANTPHOS	1,4-dioxane	65	1:1.2:2	37	14
13	$Ni(acac)_2$	NIXANTPHOS	1,4-dioxane	65	1:1.2:2	0	0
14	$Ni(COD)_2$	NIXANTPHOS	1,4-dioxane	65	1:1.2:2	0	0
15	$NiBr_2$	NIXANTPHOS	1,4-dioxane	65	1:1.2:2	0	0
16[c]	$Pd(dba)_2$	NIXANTPHOS	1,4-dioxane	65	1:1.2:2	0	0
17[d]	$Pd(dba)_2$	NIXANTPHOS	1,4-dioxane	65	1:1.2:2	0	0
18[e]	$Pd(dba)_2$	NIXANTPHOS	1,4-dioxane	65	1:1.2:2	10	0
19[f]	$Pd(dba)_2$	NIXANTPHOS	1,4-dioxane	65	1:1.2:2	7	0
20[g]	$Pd(dba)_2$	NIXANTPHOS	1,4-dioxane	65	1:1.2:2	0	0
21	$Pd(dba)_2$	NIXANTPHOS	1,4-dioxane	65	1:1.2:3	89	7

22	$Pd(dba)_2$	dppf	1,4-dioxane	65	1:1.2:3	5	21
23	$Pd(dba)_2$	dppp	1,4-dioxane	65	1:1.2:3	7	19
24	$Pd(dba)_2$	dppb	1,4-dioxane	65	1:1.2:3	22	10
25	$Pd(dba)_2$	dppe	1,4-dioxane	65	1:1.2:3	4	0
26	$Pd(dba)_2$	Xantphos	1,4-dioxane	65	1:1.2:3	28	6
27[h]	$Pd(dba)_2$	NIXANTPHOS	1,4-dioxane	65	1:1.2:3	81	5
28[i]	$Pd(dba)_2$	NIXANTPHOS	1,4-dioxane	65	1:1.2:3	72	3
29	$Pd(dba)_2$	NIXANTPHOS	1,4-dioxane	80	1:1.2:3	93	4
30	$Pd(dba)_2$	NIXANTPHOS	1,4-dioxane	100	1:1.2:3	89	trace
31	$Pd(dba)_2$	NIXANTPHOS	1,4-dioxane	rt	1:1.2:3	0	0
**32**	**$Pd(dba)_2$**	NIXANTPHOS	**1,4-dioxane**	**80**	**1:1.5:3**	**97 (92)[j]**	**trace**
33[k]	$Pd(dba)_2$	NIXANTPHOS	1,4-dioxane	80	1:1.5:3	0	0
34[l]	$Pd(dba)_2$	NIXANTPHOS	1,4-dioxane	80	1:1.5:3	65	4
35[m]	$Pd(dba)_2$	NIXANTPHOS	1,4-dioxane	80	1:1.5:3	93	trace
36[n]	$Pd(dba)_2$	NIXANTPHOS	1,4-dioxane	80	1:1.5:3	4	0
37	$Pd(dba)_2$	/	1,4-dioxane	80	1:1.5:3	0	0
38	/	NIXANTPHOS	1,4-dioxane	80	1:1.5:3	0	0

[a]**1a** (0.1 mmol, 1equiv), Pd source (5 mol%), ligand (6 mol%), solvent (0.1M). [b]Assay yields (AY) were determined by ^1H NMR analysis of unpurified reaction mixtures with internal standard CH_2Br_2. [c]12-crown-4 (2 equiv) was used. [d]TMEDA (2 equiv) was used. [e]$NaN(SiMe_3)_2$ (3 equiv) was employed instead of $LiN(SiMe_3)_2$. [f]$KN(SiMe_3)_2$ (3 equiv) was employed instead of $LiN(SiMe_3)_2$. [g]LDA (lithium diisopropylamide) (3 equiv) was employed instead of $LiN(SiMe_3)_2$. [h]1,4-Dioxane (0.2 mL) was employed. [i]1,4-Dioxane (0.05 mL) was employed. [j]Isolated yield. [k]1-Chloro-4-tertbutylbenzene was employed. [l]$Pd(dba)_2$ (2.5 mol%), NIXANTPHOS (3.0 mol%) was used. [m]Reaction time 8 h. [n]CO (8.6 atm) was imployed. Pd G3 dimer: Buchwald G3 precatalysts; Pd G4 dimer: Buchwald G4 precatalysts; acac: acetylacetonate; COD: 1,5-cyclooctadiene; dba: dibenzylideneacetone.

Table S3. Substrates that failed under the reaction conditions.[a]

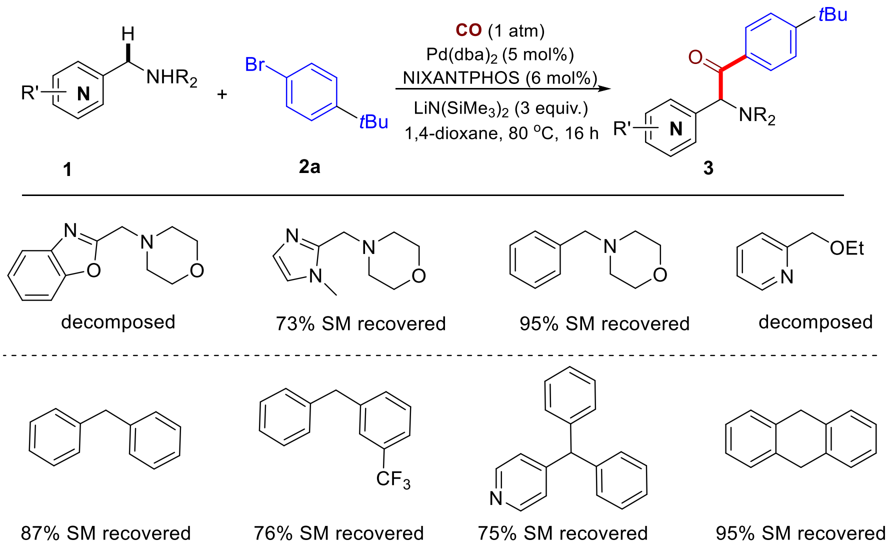

[a]Reaction conditions: **1** (0.2 mmol), **2a** (0.3 mmol), $Pd(dba)_2$ (5 mol%), NIXANTPHOS (6 mol%), $LiN(SiMe_3)_2$ (3.0 equiv), 1,4-dioxane (2.0 mL), 80 °C, 16 h, under CO atmosphere (1 atm).

3. General Procedure for DCCC of Azaarylmethyl Amines

(a) General procedure under 1 atm CO.

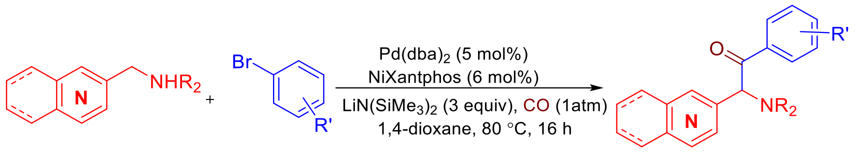

An oven-dried 8 mL vial equipped with a stir bar was charged with Pd(dba)$_2$ (5 mol %) and NIXANTPHOS (6 mol %) under a nitrogen atmosphere in glove box. Next, 0.1M of 1,4-dioxane was taken up by syringe and added to the vail. The resulting solution stirred for 15 min at room temperature, during which time the mixture became red. This solution was used as the stock solution for this procedure. To an oven-dried 10 mL Schlenk tube with stir bar was added LiN(SiMe$_3$)$_2$ (100.5 mg, 0.6 mmol, 3 equiv). A pipette was used to take 2 mL of the Pd/NIXANTPHOS stock solution and add it to the Schlenk tube. The resulting (dark green) solution was then stirred for 10 min at room temperature. Azaarylmethyl amine **1** (0.2 mmol, 1 equiv) and aryl bromide **2** (0.3 mmol, 1.5 equiv) were added to the reaction mixture, sequentially. The Schlenk tube was capped with rubber stopper and removed from the glove box. The reaction mixture was then degassed with CO by using Schlenk line, and connected with a CO balloon, placed in an 80 °C oil bath and stirred for 16 h. After this time, the flask was rem and stirred for 16 h at 80 °C. After this time, the tube was removed from the oil bath, allowed to cool to room temperature, uncapped carefully in a fumehood and the reaction quenched with two drops of H_2O. After quench the color of the reaction mixture changed from brown to red. It was next diluted with 3.0 mL of ethyl acetate and filtered over a pad of $MgSO_4$ and Celite. The pad was rinsed with additional ethyl acetate (5.0 mL) and the resulting solution evaporated under vacuum to remove the volatile materials. The residue was purified by column chromatography on silica gel using a mixture of ethyl acetate and hexanes to give the pureified product.

(b) General procedure at high CO pressure.

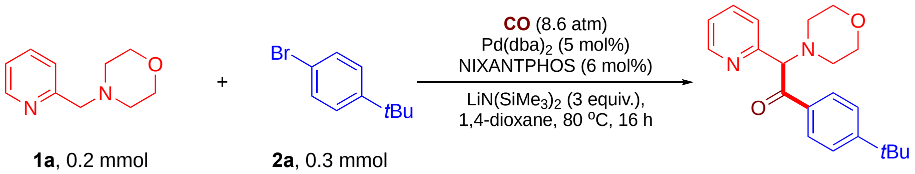

1a, 0.2 mmol **2a**, 0.3 mmol

3aa, 4%

An oven-dried 8 mL vial equipped with a stir bar was sequentially added Pd(dba)$_2$ (5.8 mg, 0.01 mmol, 5 mol %) and NIXANTPHOS (6.6 mg, 0.012 mmol, 6 mol %) under a nitrogen atmosphere inside a glove box. Next, 2 mL of 1,4-dioxane was taken up by syringe and added to the flask at room temperature. The reaction mixture was stirred for 15 min at room temperature, until the mixture became red. Then LiN(SiMe$_3$)$_2$ (100.5 mg, 0.6 mmol, 3 equiv) was added and the reaction mixture was stirred for 10 min at room temperature. Azaarylmethyl amine **1** (0.2 mmol, 1 equiv) and aryl bromide **2** (0.3 mmol, 1.5 equiv) were added to the reaction mixture, sequentially. The solution was then transferred to a 30 mL Parr Instruments 5000 Multiple Reactor system vessel. The reactor was then sealed, removed from the glovebox. The reaction vessel was then pressurized with CO at 8.6 atm. Reaction was run for 16 hours at

80 °C. After this time, reactor was cooled room temperature. The CO pressure was slowly released in a fume hood. Then the reactor was uncapped, and the reaction mixture was quenched with two drops of H_2O. The color of the reaction mixture changed from brown to red. It was next diluted with 3.0 mL of ethyl acetate and filtered over a pad of $MgSO_4$ and Celite. The pad was rinsed with additional ethyl acetate (5.0 mL) and the resulting solution evaporated under vacuum to remove the volatile materials. The assay yield was determined based on ^1H NMR analysis by integration (4% AY).

4. The general procedure for synthetic applications

a) Gram-scale synthesis

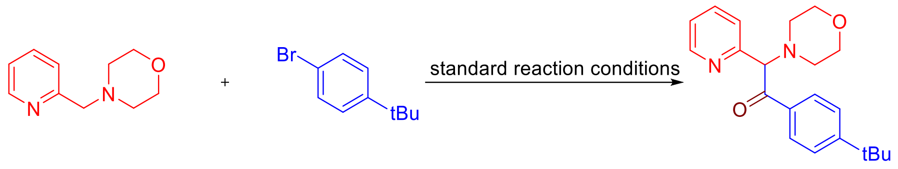

1a, 5 mmol **2a**, 7.5 mmol **3aa**, 71%

To an oven-dried 100 mL Schlenk flask with a stir bar were sequentially added $Pd(dba)_2$ (143.8 mg, 0.25 mmol, 5 mol %) and NIXANTPHOS (165.5 mg, 0.3 mmol, 6 mol %) under a nitrogen atmosphere inside a glove box. Next, 50 mL of 1,4-dioxane was taken up by syringe and added to the flask at room temperature. The reaction mixture was stirred for 30 min at room temperature, until the mixture became red. Then added $LiN(SiMe_3)_2$ (2.5 g, 15 mmol, 3 equiv) and stirred for 20 min at room temperature. Pro-nucleophile **1a** (891.0 mg, 5 mmol, 1 equiv) and 4-*tert*-butyl-bromobenzen **2a** (1.6 g, 7.5 mmol, 1.5 equiv) were added to the reaction mixture sequentially. The Schlenk flask was capped, removed from the glove box, the reaction mixture was degassed with CO by using Schlenk line (the Schlenk flask was evacuated by Schlenk line and then refiled with CO gas), then connected with a CO balloon, and placed in an 80 °C oil bath and stirred for 16 h. After this time, the flask was removed from the oil bath, allowed to cool to room temperature, then the cap was carefully removed in the fume hood, exposing the solution to the atmosphere, and the reaction quenched with H_2O (1 mL). The color of the reaction mixture changed from dark brown to red. It was next diluted with 30 mL of ethyl acetate and filtered over a pad of $MgSO_4$ and celite. The pad was rinsed with additional ethyl acetate (50 mL) and evaporated under vacuum to remove the volatile materials. The residue was purified by column chromatography on silica gel using a mixture of ethyl acetate/hexanes (1/2, v/v) to give the pure product **3aa** (1.20 g, 71%) as yellow oil.

b) Oxidation of 3aa

i) Conditions 1. When *m*-CPBA was employed as an oxidant.[3]

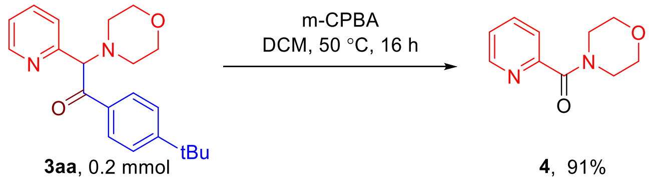

3aa, 0.2 mmol **4**, 91%

A 20 mL reaction vial was charged with a stir bar and solution of 1-(4-(*tert*-butyl)phenyl)-2-morpholino-2-(pyridin-2-yl)ethan-1-one **3aa** (67.7 mg, 0.2 mmol) in 3 mL $CHCl_3$. To the

resulting clear solution was added *m*-CPBA as a solid (138.1 mg, 4 equiv) at room temperature with stirring, resulting in a brown suspension. The reaction mixture was heated to 50 °C in an oil bath and stirred for 16 h at this temperature. The reaction mixture was then allowed to cool to room temperature, quenched with 3 mL a solution of K_2CO_3 (10%w/w) and extracted with CH_2Cl_2 (3x5 mL). The combined organic layers were washed with brine, dried over Na_2SO_4, filtered and concentrated under reduced pressure to remove the volatile materials. The resulting brown crude oil was purified by flash chromatography on silica gel (eluted with hexanes/ethyl acetate = 1/1) to give the product **4** in 91% yield as a light-yellow oil. Characterization of **4** is given below.

ii) Conditions 2. When placing **3aa** directly under air atmosphere.

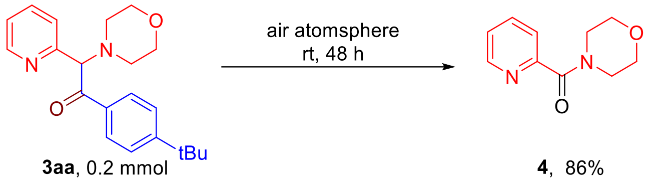

3aa, 0.2 mmol　　**4**, 86%

A 20 mL reaction vial was charged with a stir bar and a solution of 1-(4-(*tert*-butyl)phenyl)-2-morpholino-2-(pyridin-2-yl)ethan-1-one **3aa** (67.7 mg, 0.2 mmol) in 3 mL $CHCl_3$. The open vial was stirred under air atmosphere for 48 h at room temperature. The reaction mixture was concentrated under reduced pressure. The brown crude oil was purified by flash chromatography on silica gel (eluted with hexanes/ethyl acetate = 1/1) to give the product **4** in 86% yield (33.1 mg) as a light-yellow oil.

morpholino(pyridin-2-yl)methanone (4)

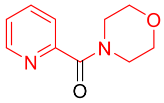

Compound **4** was prepared following the general procedure, purified by column chromatography using EtOAc/hexanes (1:1, v/v), and isolated as a light yellow oil, 35.0 mg, 91%, ^{1}H NMR (400 MHz, $CDCl_3$) δ 8.65 – 8.52 (m, 1H), 7.80 (td, *J* = 7.7, 1.8 Hz, 1H), 7.67 (d, *J* = 7.8 Hz, 1H), 7.42 – 7.30 (m, 1H), 3.80 (s, 4H), 3.67 (hept, *J* = 3.6, 2.7 Hz, 4H). $^{13}C\{^{1}H\}$ NMR (101 MHz, $CDCl_3$) δ 167.5, 153.6, 148.2, 137.2, 124.7, 124.2, 67.0, 66.8, 47.8, 42.8. HRMS (ESI) calcd. for $C_{10}H_{12}N_2O_2$ $[M+H]^+$: 193.0972, found:193.0979.

c) Reduction of 3aa to 1-(4-(*tert*-butyl)phenyl)-2-morpholino-2-(pyridin-2-yl)ethan-1-ol (5)

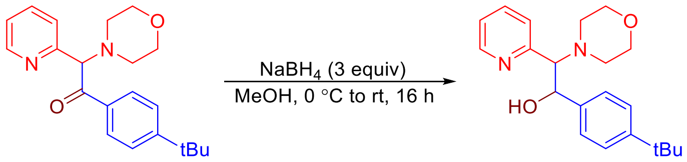

3aa, 0.4 mmol　　**5**, 68%

An 8 mL reaction vial was charged with a stir bar and a solution of 1-(4-(*tert*-butyl)phenyl)-2-morpholino-2-(pyridin-2-yl)ethan-1-one **3aa** (135.4 mg, 0.4 mmol) in 4 mL MeOH. The vial was placed in an ice water bath and stirred. To the clear solution cooled solution was added $NaBH_4$ as a solid (45.4 mg, 3 equiv) at 0 °C, which generated a brown suspension. After the addition (or in 15 min) at 0 °C, the reaction mixture was removed from

the ice water bath, allowed to warm to room temperature and stirred for 16 h. The reaction mixture was then quenched with 1 mL of a solution of NH_4Cl (10%w/w) and extracted with CH_2Cl_2 (3x5 mL). The combined organic layers were dried over Na_2SO_4, filtered and concentrated under reduced pressure to remove the volatile materials. The crude brown oil was purified by flash chromatography on silica gel (eluted with hexanes/ethyl acetate = 1/1) to give the product **5** (68% yield, 92.6 mg) as a light-yellow oil. ^1H NMR (400 MHz, $CDCl_3$) δ 8.54 (ddd, *J* = 5.0, 1.8, 0.9 Hz, 1H), 7.48 (td, *J* = 7.7, 1.9 Hz, 1H), 7.19 – 7.13 (m, 3H), 7.04 – 6.95 (m, 2H), 6.91 (dt, *J* = 7.8, 1.1 Hz, 1H), 5.79 (s, 1H), 5.38 (d, *J* = 3.9 Hz, 1H), 3.71 (ddd, *J* = 6.0, 3.6, 2.6 Hz, 4H), 3.53 (d, *J* = 4.0 Hz, 1H), 2.67 (dt, *J* = 10.1, 4.8 Hz, 2H), 2.56 (ddd, *J* = 11.4, 5.4, 3.6 Hz, 2H), 1.23 (s, 9H). $^{13}C\{^1H\}$ NMR (101 MHz, $CDCl_3$) δ 158.2, 149.6, 148.3, 139.7, 136.2, 125.7, 125.6, 124.7, 122.6, 74.8, 72.8, 67.1, 51.5, 34.3, 31.3. HRMS (ESI) calcd. for $C_{21}H_{28}N_2O_2$ $[M+H]^+$: 341.2224, found:341.2219.

d) Synthesis of (E)-allyl (1-(4-(tert-butyl)phenyl)-2-morpholino-2-(pyridin-2-yl)vinyl) carbonate (6)[4]

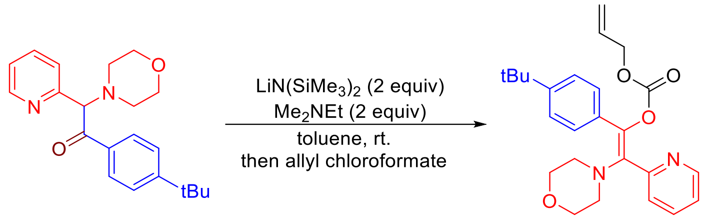

3aa, 0.3 mmol　　**6**, 87%

To an oven-dried Schlenk tube with a stir bar was added $LiN(SiMe_3)_2$ (100.4 mg, 0.6 mmol) followed by toluene (1.0 mL) and *N,N*-dimethylethylamine (43 μL) in drybox. The resulting mixture stirred at 25 °C for 5 min. Next, a solution of ketone **3aa** (0.3 mmol) in toluene (1.0 mL) was then added and the reaction mixture stirred at 25 °C for an additional 30 min generating a yellow solution. The tube was then placed in a room temperature water bath and allyl chloroformate (63.8 μL, 0.6 mmol) was added slowly over 5 min. The reaction was allowed to stir until no starting material remained by TLC (typically less than 1 h). The crude reaction mixture was diluted with Et_2O (5 mL) and then quenched with water. The color of the reaction mixture changed from red to light brown. The layers were separated, and the aqueous layer was extracted with Et_2O (5 mL) twice. The combined organic layers were dried over Na_2SO_4, filtered and concentrated. The crude product was purified by silica gel flash chromatography (eluted with hexanes/ethyl acetate = 2/1) to afford the desired enol carbonate **6** in 87% yield as a yellow oil, 110.3 mg, 87%. ^1H NMR (400 MHz, $CDCl_3$) δ 8.67 – 8.58 (m, 1H), 7.68 (td, *J* = 7.7, 1.9 Hz, 1H), 7.54 (dt, *J* = 7.7, 1.1 Hz, 1H), 7.49 – 7.43 (m, 2H), 7.42 – 7.34 (m, 2H), 7.19 (ddd, *J* = 7.6, 4.8, 1.2 Hz, 1H), 5.91 – 5.70 (m, 1H), 5.27 – 5.13 (m, 2H), 4.52 (dt, *J* = 5.7, 1.5 Hz, 2H), 3.62 (t, *J* = 4.7 Hz, 4H), 2.72 (t, *J* = 4.6 Hz, 4H), 1.32 (s, 9H). $^{13}C\{^1H\}$ NMR (101 MHz, $CDCl_3$) δ 155.5, 154.1, 151.1, 149.5, 139.8, 136.8, 136.2, 132.4, 131.5, 127.8, 125.0, 124.2, 122.7, 118.5, 68.5, 67.4, 50.9, 34.7, 31.3. HRMS (ESI) calcd. for $C_{25}H_{30}N_2O_4$ $[M+H]^+$: 423.2278, found: 423.2283.

5. Reaction studies

a) Detection of the enolate:

i) Deprotonatation of **3aa**:

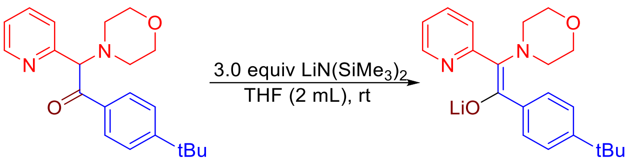

3aa, 0.2 mmol **3aA**

An oven-dried 20 mL vial equipped with a stir bar was charged with **3aa** (67.7 mg, 0.2 mmol) under a nitrogen atmosphere in the glove box. A solution of $LiN(SiMe_3)_2$ (100.5 mg, 0.6 mmol) in 2.0 mL of dry THF was added with stirring at room temperature. After stirring for 3 h at room temperature, the color had changed from colorless to yellow. The resulting solution was evaporated under vacuum to remove the volatile materials. The resulting oil was taken up in 0.5 mL dry d^8-THF. The suspension formed was filtered through dry celite and the filtrate was carefully transferred to J-Young NMR tube that was then sealed. NMR data was then collected and the ^{1}H NMR and ^{13}C{^{1}H} NMR spectrum are shown below.

ii) Reaction monitoring:

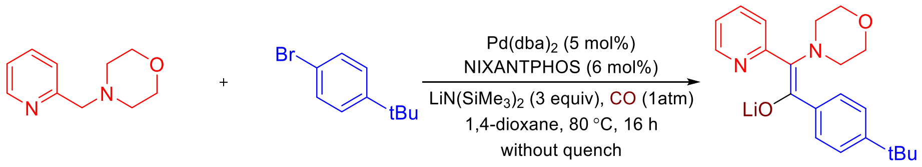

1a, 0.1 mmol **2a**, 0.15 mmol **3aA**

An oven-dried 8 mL vial equipped with a stir bar under a nitrogen atmosphere in glove box was charged with $Pd(dba)_2$ (5 mol %), NIXANTPHOS (6 mol %) and 0.1 M of 1,4-dioxane was taken up by syringe and added to the vail. The resulting solution was stirred for 15 min at room temperature during which time the mixture became red. This solution was used as the stock solution. To an oven-dried 10 mL Schlenk tube in the glove box with stir bar was added $LiN(SiMe_3)_2$ (50.3 mg, 0.3 mmol, 3 equiv). Next, 1 mL of the stock solution was added by pipette and the resulting solution stirred for 10 min at room temperature. 2-Pyridylmethylmorpholine **1a** (17.8 mg, 0.1 mmol, 1 equiv) and aryl bromide **2a** (32 mg, 0.15 mmol, 1.5 equiv) were sequentially added to the reaction mixture. The Schlenk tube was capped, removed from the glove box and the reaction mixture was degassed with CO by using Schlenk line, and connected with a CO balloon stirred for 16 h at 80 °C. After this time, the tube was removed from the oil bath, allowed to cool to room temperature, connected to a Schlenk line and evaporated under reduced pressure. While under vacuum, the tube was brought back into the glove box, the cap was carefully removed, and 0.5 mL dry d^8-THF was added to the crude reaction mixture. The suspension was filtered through dry celite and the filtrate was carefully transferred to J-Young tube and NMR spectra acquired.

Supplementary Figure S1: ^{1}H NMR comparison of standard carbonylation product before aqueous workup, deprotonated **3aa**, and product **3aa** in d^8-THF. These spectra support the contention that the product formed in the carbonylation reaction before workup is the enolate.

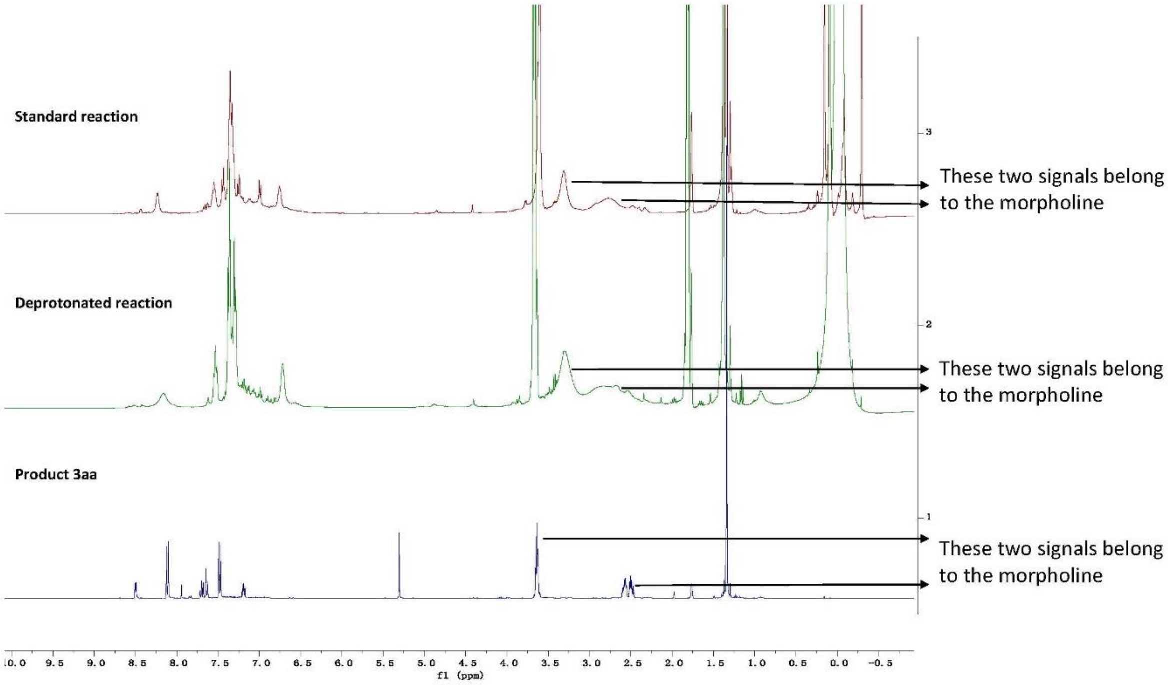

Supplementary Figure S2: $^{13}C\{^1H\}$ NMR comparison of standard carbonylation product before aqueous workup, deprotonated **3aa**, and product **3aa** in *d*^{8}-THF. These spectra support the contention that the product formed in the carbonylation reaction before workup is the enolate.

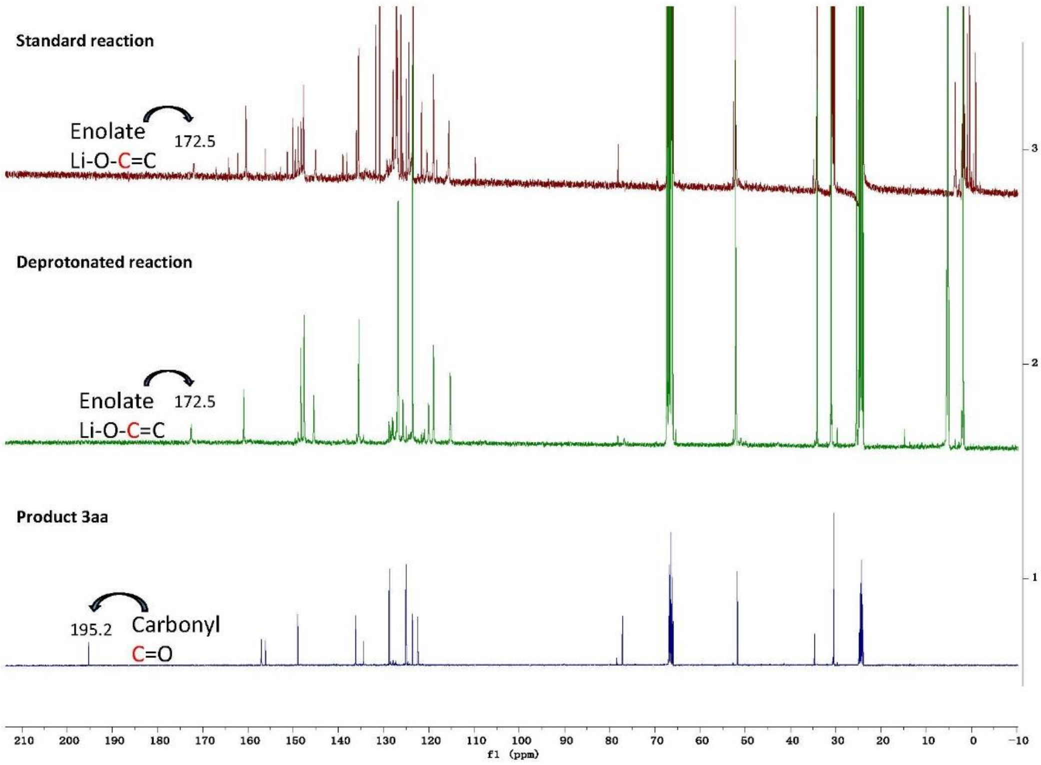

6. Characterization data for products

1-(4-(*tert*-butyl)phenyl)-2-morpholino-2-(pyridin-2-yl)ethan-1-one (3aa)

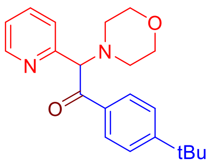

Compound **3aa** was prepared following the general procedure, purified by column chromatography using EtOAc/hexanes (1:2, v/v), and isolated as a yellow oil, 62.3 mg, 92%, ^{1}H NMR (400 MHz, $CDCl_3$) δ 8.57 (ddd, *J* = 4.9, 1.8, 0.9 Hz, 1H), 8.10 – 8.02 (m, 2H), 7.65 (td, *J* = 7.7, 1.9 Hz, 1H), 7.58 (dt, *J* = 7.8, 1.1 Hz, 1H), 7.45 – 7.39 (m, 2H), 7.17 (ddd, *J* = 7.4, 4.9, 1.3 Hz, 1H), 5.25 (s, 1H), 3.81 – 3.68 (m, 4H), 2.62 (ddd, *J* = 9.9, 5.9, 3.4 Hz, 2H), 2.46 (tdd, *J* = 9.2, 4.5, 2.2 Hz, 2H), 1.30 (s, 9H). $^{13}C\{^{1}H\}$ NMR (101 MHz, $CDCl_3$) δ 196.2, 157.1, 156.0, 149.5, 136.9, 133.8, 129.0, 125.5, 123.9, 123.0, 77.7, 66.9, 52.1, 35.1, 31.0. HRMS (ESI) calcd. for $C_{21}H_{26}N_2O_2$ $[M+H]^+$: 339.2067, found: 339.2061.

1-(4-(*tert*-butyl)phenyl)-2-(pyridin-2-yl)-2-thiomorpholinoethan-1-one (3ba)

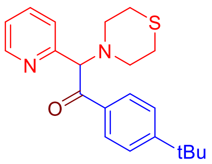

Compound **3ba** was prepared following the general procedure, purified by column chromatography using EtOAc/hexanes (1:2, v/v), and isolated as a brown oil, 61.6 mg, 87%, ^{1}H NMR (400 MHz, $CDCl_3$) δ 8.55 (ddd, *J* = 4.9, 1.8, 0.9 Hz, 1H), 8.03 – 7.97 (m, 2H), 7.65 (td, *J* = 7.7, 1.8 Hz, 1H), 7.51 (dt, *J* = 7.9, 1.1 Hz, 1H), 7.43 – 7.37 (m, 2H), 7.17 (ddd, *J* = 7.5, 4.9, 1.2 Hz, 1H), 5.37 (s, 1H), 2.91 – 2.82 (m, 4H), 2.69 (t, *J* = 4.6 Hz, 4H), 1.29 (s, 9H). $^{13}C\{^{1}H\}$ NMR (101 MHz, $CDCl_3$) δ 197.0, 157.0, 156.2, 149.5, 136.7, 134.0, 128.9, 125.5, 124.0, 122.8, 77.0, 53.4, 35.1, 31.0, 28.0. HRMS (ESI) calcd. for $C_{21}H_{26}N_2OS$ $[M+H]^+$: 355.1839, found: 355.1835.

1-(4-(*tert*-butyl)phenyl)-2-(4-methylpiperazin-1-yl)-2-(pyridin-2-yl)ethan-1-one (3ca)

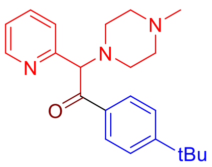

Compound **3ca** was prepared following the general procedure, purified by column chromatography using DCM/MeOH (10:1, v/v), and isolated as a brown oil, 48.5 mg, 69%, ^{1}H NMR (400 MHz, $CDCl_3$) δ 8.53 (dd, *J* = 4.9, 1.6 Hz, 1H), 8.07 – 7.95 (m, 2H), 7.62 (td, *J* = 7.7, 1.8 Hz, 1H), 7.52 (d, *J* = 7.8 Hz, 1H), 7.38 (d, *J* = 8.4 Hz, 2H), 7.14 (ddd, *J* = 7.5, 4.8, 1.2 Hz, 1H), 5.27 (s, 1H), 2.70 (s, 5H), 2.61 (dd, *J* = 10.4, 5.0 Hz, 3H), 2.40 (s, 3H), 1.26 (s, 9H). $^{13}C\{^{1}H\}$ NMR (101 MHz, $CDCl_3$) δ 196.1, 157.2, 155.8, 149.6, 136.9, 133.6, 129.0, 125.5, 123.9, 123.0, 76.8, 54.6, 50.3, 45.2, 35.1, 31.0. HRMS (ESI) calcd. for $C_{22}H_{29}N_3O$ $[M+H]^+$: 352.2383, found: 352.2377.

1-(4-(*tert*-butyl)phenyl)-2-(dimethylamino)-2-(pyridin-2-yl)ethan-1-one (3da)

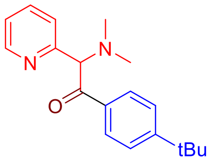

Compound **3da** was prepared following the general procedure, purified by column chromatography using EtOAc/hexanes (1:1, v/v), and isolated as a yellow oil, 43.3 mg, 73%, ^{1}H NMR (400 MHz, $CDCl_3$) δ 8.56 (ddd, *J* = 4.9, 1.9, 1.0 Hz, 1H), 8.12 – 8.01 (m, 2H), 7.65 (td, *J* = 7.7, 1.8 Hz, 1H), 7.57 (dt, *J* = 8.0, 1.2 Hz, 1H), 7.43 – 7.39 (m, 2H), 7.17 (ddd, *J* = 7.4, 4.9, 1.4 Hz, 1H), 5.15 (s, 1H), 2.31 (s, 6H), 1.29 (s, 9H). $^{13}C\{^{1}H\}$ NMR (101 MHz, $CDCl_3$) δ 196.8, 157.0, 156.9, 149.4, 136.9, 133.7, 129.0, 125.5, 123.7, 122.9, 78.4, 43.9, 35.1, 31.0. HRMS (ESI) calcd. for $C_{19}H_{24}N_2O$ $[M+H]^+$: 297.1961, found: 297.1965.

1-(4-(*tert*-butyl)phenyl)-2-(pyridin-2-yl)-2-(pyrrolidin-1-yl)ethan-1-one (3ea)

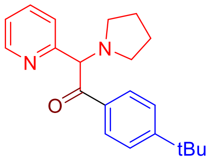

Compound **3ea** was prepared following the general procedure, purified by column chromatography using EtOAc/hexanes (1:2, v/v), and isolated as a yellow oil, 54.2 mg, 84%, ^{1}H NMR (400 MHz, $CDCl_3$) δ 8.55 (dt, *J* = 4.9, 1.3 Hz, 1H), 8.15 – 8.03 (m, 2H), 7.69 – 7.54 (m, 2H), 7.44 – 7.36 (m, 2H), 7.16 (ddd, *J* = 6.8, 4.9, 1.6 Hz, 1H), 5.20 (s, 1H), 2.73 (dt, *J* = 8.4, 6.4 Hz, 2H), 2.40 (dq, *J* = 8.4, 5.0, 4.0 Hz, 2H), 1.87 – 1.76 (m, 4H), 1.29 (s, 9H). ^{13}C{^{1}H} NMR (101 MHz, $CDCl_3$) δ 196.1, 157.4, 156.9, 149.2, 136.9, 133.4, 129.1, 125.4, 123.5, 122.8, 77.7, 52.6, 35.1, 31.0, 23.3. HRMS (ESI) calcd. for $C_{21}H_{26}N_2O$ [M+H]$^+$: 323.2118, found: 323.2112.

1-(4-(*tert*-butyl)phenyl)-2-(piperidin-1-yl)-2-(pyridin-2-yl)ethan-1-one (3fa)

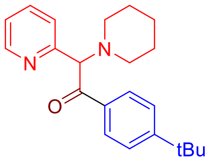

Compound **3fa** was prepared following the general procedure, purified by column chromatography using EtOAc/hexanes (1:2, v/v), and isolated as a yellow oil, 43.1 mg, 64%, ^{1}H NMR (400 MHz, $CDCl_3$) δ 8.54 (dt, *J* = 4.8, 1.4 Hz, 1H), 8.13 – 8.03 (m, 2H), 7.67 – 7.59 (m, 2H), 7.43 – 7.39 (m, 2H), 7.15 (ddd, *J* = 6.8, 4.9, 1.9 Hz, 1H), 5.23 (s, 1H), 2.52 (dt, *J* = 10.8, 5.3 Hz, 2H), 2.42 (dq, *J* = 11.0, 5.5, 4.8 Hz, 2H), 1.60 (q, *J* = 4.9 Hz, 4H), 1.44 (q, *J* = 5.9 Hz, 2H), 1.30 (s, 9H). ^{13}C{^{1}H} NMR (101 MHz, $CDCl_3$) δ 197.4, 157.0, 156.8, 149.2, 136.6, 134.2, 129.0, 125.4, 123.9, 122.6, 78.2, 52.9, 35.1, 31.0, 26.0, 24.4. HRMS (ESI) calcd. for $C_{22}H_{28}N_2O$ [M+H]$^+$: 337.2274, found: 337.2269.

1-(4-(*tert*-butyl)phenyl)-2-(4-methoxy-3,5-dimethylpyridin-2-yl)-2-morpholinoethan-1-one (3ga)

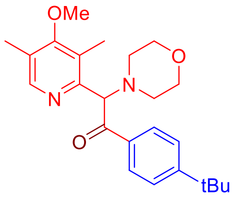

Compound **3ga** was prepared following the general procedure, purified by column chromatography using EtOAc/hexanes (1:2, v/v), and isolated as a yellow oil, 41.2 mg, 52%, ^{1}H NMR (400 MHz, $CDCl_3$) δ 8.15 (s, 1H), 7.90 – 7.82 (m, 2H), 7.37 – 7.31 (m, 2H), 5.34 (s, 1H), 3.75 (s, 3H), 3.68 (dt, *J* = 6.2, 3.4 Hz, 4H), 2.70 (q, *J* = 4.3 Hz, 4H), 2.46 (s, 3H), 2.19 (s, 3H), 1.27 (s, 9H). ^{13}C{^{1}H} NMR (101 MHz, $CDCl_3$) δ 196.5, 164.5, 156.2, 154.1, 149.3, 134.1, 128.5, 127.1, 125.6, 125.3, 74.8, 67.5, 59.9, 50.9, 35.0, 31.0, 13.3, 11.0. HRMS (ESI) calcd. for $C_{24}H_{32}N_2O_3$ [M+H]$^+$: 397.2486, found: 397.2480.

1-(4-(*tert*-butyl)phenyl)-2-(pyridin-4-yl)-2-thiomorpholinoethan-1-one (3ha)

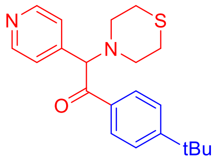

Compound **3ha** was prepared following the general procedure, purified by column chromatography using EtOAc/hexanes (1:2, v/v), and isolated as a brown oil, 57.4 mg, 81%, ^{1}H NMR (400 MHz, $CDCl_3$) δ 8.59 – 8.50 (m, 2H), 7.99 – 7.90 (m, 2H), 7.46 – 7.40 (m, 2H), 7.36 – 7.30 (m, 2H), 5.07 (s, 1H), 2.86 (dt, *J* = 11.6, 5.1 Hz, 2H), 2.78 (dt, *J* = 11.7, 4.8 Hz, 2H), 2.67 (t, *J* = 5.0 Hz, 4H), 1.30 (s, 9H). ^{13}C{^{1}H} NMR (101 MHz, $CDCl_3$) δ 196.5, 157.6, 150.1, 144.6, 133.5, 128.7, 125.7, 124.4, 74.6, 53.3, 35.2, 31.0, 28.1. HRMS (ESI) calcd. for $C_{21}H_{26}N_2OS$ [M+H]$^+$: 355.1839, found: 355.1835.

1-(4-(*tert*-butyl)phenyl)-2-morpholino-2-(pyridin-4-yl)ethan-1-one (3ia)

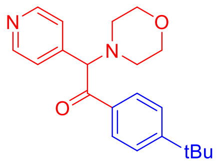

Compound **3ia** was prepared following the general procedure, purified by column chromatography using EtOAc/hexanes (1:2, v/v), and isolated as a yellow oil, 59.6 mg, 88%, ^{1}H NMR (400 MHz, $CDCl_3$) δ 8.63 (s, 2H), 8.05 – 7.92 (m, 2H), 7.51 – 7.38 (m, 4H), 4.92 (s, 1H), 3.73 (ddd, *J* = 5.9, 3.8, 2.5 Hz, 4H), 2.59 – 2.44 (m, 4H), 1.29 (s, 9H). ^{13}C{^{1}H} NMR (101 MHz, $CDCl_3$) δ 195.8, 157.7, 150.1, 144.3, 133.4, 128.7, 128.3, 125.7, 75.2, 66.9, 52.0, 35.2, 31.0. HRMS (ESI) calcd. for $C_{21}H_{26}N_2OS$ $[M+H]^+$: 339.2067, found: 339.2061.

1-(4-(*tert*-butyl)phenyl)-2-(dimethylamino)-2-(pyridin-4-yl)ethan-1-one (3ja)

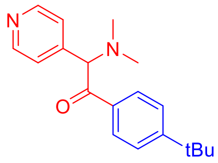

Compound **3ja** was prepared following the general procedure, purified by column chromatography using EtOAc/hexanes (1:1, v/v), and isolated as a yellow oil, 45.6 mg, 77%, ^{1}H NMR (400 MHz, $CDCl_3$) δ 8.60 – 8.51 (m, 2H), 8.01 – 7.93 (m, 2H), 7.44 – 7.36 (m, 4H), 4.83 (s, 1H), 2.30 (s, 6H), 1.30 (s, 9H). ^{13}C{^{1}H} NMR (101 MHz, $CDCl_3$) δ 196.4, 157.5, 150.1, 145.1, 133.3, 128.7, 125.7, 124.3, 75.7, 43.8, 35.2, 31.0. HRMS (ESI) calcd. for $C_{19}H_{24}N_2O$ $[M+H]^+$: 297.1961, found: 297.1965.

1-(4-(*tert*-butyl)phenyl)-2-(4-methylpiperazin-1-yl)-2-(pyridin-2-yl)ethan-1-one (3ka)

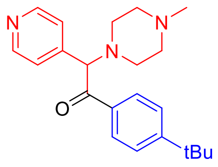

Compound **3ka** was prepared following the general procedure, purified by column chromatography using DCM/MeOH (10:1, v/v), and isolated as a brown oil, 42.9 mg, 61%, ^{1}H NMR (400 MHz, $CDCl_3$) δ 8.57 – 8.47 (m, 2H), 8.01 – 7.89 (m, 2H), 7.46 – 7.33 (m, 4H), 4.94 (s, 1H), 2.81 – 2.43 (m, 8H), 2.37 (s, 3H), 1.28 (s, 9H). ^{13}C{^{1}H} NMR (101 MHz, $CDCl_3$) δ 195.8, 157.7, 150.2, 144.5, 133.3, 128.7, 125.7, 124.3, 74.6, 54.7, 50.6, 45.3, 35.2, 31.0. HRMS (ESI) calcd. for $C_{22}H_{29}N_3O$ $[M+H]^+$: 352.2383, found: 352.2377.

2-(benzo[d]thiazol-2-yl)-1-(4-(tert-butyl)phenyl)-2-morpholinoethan-1-one : (*E*)-2-(benzo[d]thiazol-2-yl)-1-(4-(tert-butyl)phenyl)-2-morpholinoethen-1-ol = 3:1 (3la)

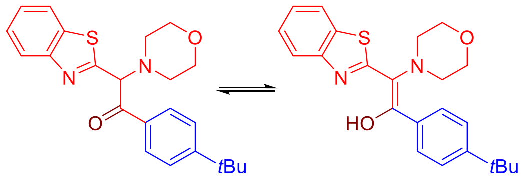

Compound **3la** was prepared following the general procedure, purified by column chromatography using EtOAc/hexanes (1:4, v/v), and isolated as a white semi solid, 19 mg, 50%, ^{1}H NMR (400 MHz, C_6D_6) 8.19 (d, J = 8.0 Hz, 2H, keto-phenacylCH), 8.00 (d, J = 8.0 Hz, 1H, keto-benzothiazoleC(7)H), 7.65 (d, J = 8.0 Hz, 2H, enol-phenacylCH), 7.64 (d, J = 8.0 Hz, 1H, enol-benzothiazoleC(7)H)), 7.51 (d, J = 8.0 Hz, 1H, enol-benzothiazoleC(4)H), 7.40 (d, J = 8.0 Hz, 1H, keto-benzothiazoleC(4)H), 7.34 (d, J = 8.0 Hz, 2H, keto-phenacylCH), 7.16 (d, 2H, keto-phenacylCH), 7.13 (t, J = 8.0 Hz, 1H, enol-benzothiazoleC(5)H), 7.07 (t, J = 8.0 Hz, 1H, keto-benzothiazoleC(5)H), 7.00 (t, J = 8.0 Hz, 1H, enol-benzothiazoleC(6)H), 6.96 (t, J = 8.0 Hz, 1H, keto-benzothiazoleC(6)H), 5.68 (s, 1H, keto-CHCOAr), 3.61 – 3.48 (m, 4H, keto-morpholineCH, 4H, enol-morpholineCH), 2.79 – 2.52 (m, 4H, keto-morpholineCH, 4H, enol-morpholineCH), 1.21 (s, 9H, enol-tertbutylCH), 1.07 (s, 9H, keto-tertbutylCH). ^{13}C{^{1}H} NMR (101 MHz, C_6D_6) δ 193.6, 176.1, 167.4, 162.5, 156.9, 153.0, 152.8, 152.4, 136.3, 134.0, 133.3, 133.0, 129.1, 128.3, 126.1, 125.7, 125.5, 125.1, 125.0, 124.0, 123.4, 121.6, 121.2, 120.9, 119.4, 72.4, 67.6, 66.8, 52.0, 51.5, 34.6, 31.0, 30.6,

(several resonances is missing due to overlapping peaks). HRMS (ESI) calcd. For $C_{23}H_{26}N_2O_2S$ $[M+H]^+$: 395.1793, found: 395.1777.

1-(4-(*tert*-butyl)phenyl)-2-morpholino-2-(quinolin-2-yl)ethan-1-one (3ma)

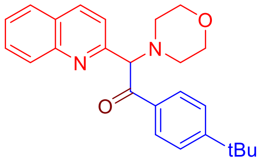

Compound **3ma** was prepared following the general procedure, purified by column chromatography using EtOAc/hexanes (1:1, v/v), and isolated as a brown oil, 57.5 mg, 74%, ^{1}H NMR (400 MHz, $CDCl_3$) δ 8.19 – 8.14 (m, 2H), 8.14 – 8.09 (m, 2H), 7.78 – 7.73 (m, 2H), 7.68 (ddd, *J* = 8.5, 6.9, 1.5 Hz, 1H), 7.50 (ddd, *J* = 8.1, 6.9, 1.2 Hz, 1H), 7.43 – 7.38 (m, 2H), 5.46 (s, 1H), 3.83 – 3.71 (m, 4H), 2.70 (ddd, *J* = 10.0, 5.9, 3.4 Hz, 2H), 2.48 – 2.38 (m, 2H), 1.26 (s, 9H). ^{13}C{^{1}H} NMR (101 MHz, $CDCl_3$) δ 196.0, 157.2, 156.5, 147.9, 136.9, 133.9, 129.6, 129.4, 129.1, 127.6, 127.6, 126.9, 125.5, 120.9, 78.4, 66.9, 52.2, 35.1, 31.0. HRMS (ESI) calcd. for $C_{25}H_{28}N_2O_2$ $[M+H]^+$: 389.2224, found: 389.2230.

1-(4-(*tert*-butyl)phenyl)-2-(dimethylamino)-2-(quinolin-2-yl)ethan-1-one (3na)

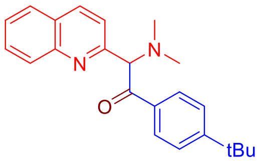

Compound **3na** was prepared following the general procedure, purified by column chromatography using EtOAc/hexanes (1:1, v/v), and isolated as a yellow oil, 49.9 mg, 72%, ^{1}H NMR (400 MHz, $CDCl_3$) δ 8.21 – 8.14 (m, 2H), 8.14 – 8.07 (m, 2H), 7.77 – 7.65 (m, 3H), 7.49 (ddd, *J* = 8.1, 6.9, 1.3 Hz, 1H), 7.42 – 7.35 (m, 2H), 5.33 (s, 1H), 2.34 (s, 6H), 1.26 (s, 9H). ^{13}C{^{1}H} NMR (101 MHz, $CDCl_3$) δ 196.6, 157.4, 157.0, 147.8, 136.9, 133.8, 129.4, 129.4, 129.2, 127.6, 127.6, 126.7, 125.4, 120.8, 79.4, 44.1, 35.1, 31.0. HRMS (ESI) calcd. for $C_{23}H_{26}N_2O$ $[M+H]^+$: 347.2118, found: 347.2113.

1-(4-(*tert*-butyl)phenyl)-2-morpholino-2-(pyrimidin-2-yl)ethan-1-one (3oa)

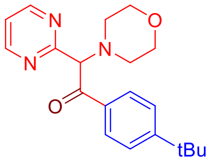

Compound **3oa** was prepared following the general procedure, purified by column chromatography using EtOAc/hexanes (1:1, v/v), and isolated as a yellow oil, 63.1 mg, 93%, ^{1}H NMR (400 MHz, $CDCl_3$) δ 8.71 (d, *J* = 4.9 Hz, 2H), 7.97 (d, *J* = 8.6 Hz, 2H), 7.42 – 7.34 (m, 2H), 7.16 (t, *J* = 4.9 Hz, 1H), 5.42 (s, 1H), 3.75 (t, *J* = 4.6 Hz, 4H), 2.68 (q, *J* = 4.0 Hz, 4H), 1.27 (s, 9H). ^{13}C{^{1}H} NMR (101 MHz, $CDCl_3$) δ 194.6, 165.6, 157.4, 156.9, 133.6, 128.8, 125.4, 119.7, 77.3, 67.1, 51.5, 35.1, 31.0. HRMS (ESI) calcd. for $C_{20}H_{25}N_3O_2$ $[M+H]^+$: 340.2020, found: 340.2016.

2-morpholino-1-phenyl-2-(pyridin-2-yl)ethan-1-one (3ab)

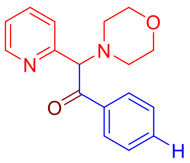

Compound **3ab** was prepared following the general procedure, purified by column chromatography using EtOAc/hexanes (1:2, v/v), and isolated as a yellow oil, 44.6 mg, 79%, ^{1}H NMR (400 MHz, $CDCl_3$) δ 8.54 (dt, *J* = 4.9, 1.4 Hz, 1H), 8.13 – 8.03 (m, 2H), 7.63 (td, *J* = 7.7, 1.8 Hz, 1H), 7.55 (d, *J* = 7.9 Hz, 1H), 7.51 – 7.44 (m, 1H), 7.38 (t, *J* = 7.8 Hz, 2H), 7.15 (ddd, *J* = 7.4, 4.9, 1.3 Hz, 1H), 5.26 (s, 1H), 3.81 – 3.68 (m, 4H), 2.61 (ddd, *J* = 10.1, 5.9, 3.4 Hz, 2H), 2.48 (ddd, *J* = 10.7, 6.0, 3.3 Hz, 2H). ^{13}C{^{1}H} NMR (101 MHz, $CDCl_3$) δ 196.7, 155.7, 149.5, 136.9, 136.4, 133.3, 129.0, 128.5, 124.0, 123.0, 77.7, 66.9, 51.9. HRMS (ESI) calcd. for $C_{17}H_{18}N_2O_2$ $[M+H]^+$: 283.1441, found: 283.1437.

2-morpholino-2-(pyridin-2-yl)-1-(p-tolyl)ethan-1-one (3ac)

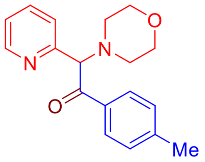

Compound **3ac** was prepared following the general procedure, purified by column chromatography using EtOAc/hexanes (1:2, v/v), and isolated as a yellow oil, 53.4 mg, 90%, ^{1}H NMR (500 MHz, $CDCl_3$) δ 8.54 (dt, *J* = 4.9, 1.4 Hz, 1H), 8.05 – 7.96 (m, 2H), 7.63 (td, *J* = 7.7, 1.8 Hz, 1H), 7.57 (d, *J* = 7.8 Hz, 1H), 7.22 – 7.12 (m, 3H), 5.24 (s, 1H), 3.79 – 3.69 (m, 4H), 2.67 – 2.58 (m, 2H), 2.48 (ddd, *J* = 10.8, 6.0, 3.4 Hz, 2H), 2.34 (s, 3H). $^{13}C\{^{1}H\}$ NMR (126 MHz, $CDCl_3$) δ 196.1, 155.8, 149.5, 144.2, 136.8, 133.9, 129.2, 129.1, 123.9, 123.0, 77.6, 66.9, 52.0, 21.6. HRMS (ESI) calcd. for $C_{18}H_{20}N_2O_2$ $[M+H]^+$: 297.1598, found: 297.1603.

1-(4-methoxyphenyl)-2-morpholino-2-(pyridin-2-yl)ethan-1-one (3ad)

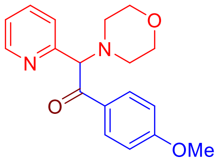

Compound **3ad** was prepared following the general procedure, purified by column chromatography using EtOAc/hexanes (1:1, v/v), and isolated as a yellow oil, 53.1 mg, 85%, ^{1}H NMR (400 MHz, $CDCl_3$) δ 8.65 – 8.40 (m, 1H), 8.22 – 8.00 (m, 2H), 7.68 – 7.51 (m, 2H), 7.15 (ddd, *J* = 7.0, 5.0, 1.4 Hz, 1H), 6.94 – 6.77 (m, 2H), 5.18 (s, 1H), 3.80 (s, 3H), 3.79 – 3.67 (m, 4H), 2.68 – 2.54 (m, 2H), 2.44 (ddd, *J* = 10.7, 6.0, 3.5 Hz, 2H). $^{13}C\{^{1}H\}$ NMR (101 MHz, $CDCl_3$) δ 195.0, 163.7, 156.1, 149.4, 136.8, 131.4, 129.4, 123.8, 122.9, 113.7, 77.7, 66.9, 55.4, 52.1. HRMS (ESI) calcd. for $C_{18}H_{20}N_2O_3$ $[M+H]^+$: 313.1547, found: 313.1552.

1-(4-(methylthio)phenyl)-2-morpholino-2-(pyridin-2-yl)ethan-1-one (3ae)

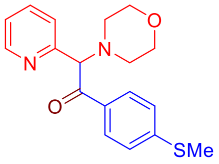

Compound **3ae** was prepared following the general procedure, purified by column chromatography using EtOAc/hexanes (1:1, v/v), and isolated as a brown oil, 42.0 mg, 64%, ^{1}H NMR (400 MHz, $CDCl_3$) δ 8.54 (ddd, *J* = 5.0, 1.8, 0.9 Hz, 1H), 8.11 – 7.96 (m, 2H), 7.64 (td, *J* = 7.7, 1.8 Hz, 1H), 7.55 (dt, *J* = 7.9, 1.1 Hz, 1H), 7.23 – 7.09 (m, 3H), 5.18 (s, 1H), 3.81 – 3.67 (m, 4H), 2.65 – 2.55 (m, 2H), 2.50 – 2.40 (m, 5H). $^{13}C\{^{1}H\}$ NMR (101 MHz, $CDCl_3$) δ 195.6, 155.8, 149.5, 146.4, 136.9, 132.5, 129.4, 124.8, 123.9, 123.0, 77.8, 66.9, 52.0, 14.6. HRMS (ESI) calcd. for $C_{18}H_{20}N_2O_2S$ $[M+H]^+$: 329.1318, found: 329.1316.

1-(4-(dimethylamino)phenyl)-2-morpholino-2-(pyridin-2-yl)ethan-1-one (3af)

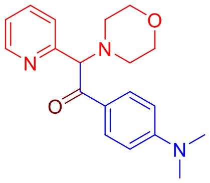

Compound **3af** was prepared following the general procedure, purified by column chromatography using EtOAc/hexanes (1:1, v/v), and isolated as a yellow oil, 48.8 mg, 75%, ^{1}H NMR (400 MHz, $CDCl_3$) δ 8.52 (dt, *J* = 4.8, 1.4 Hz, 1H), 8.13 – 7.97 (m, 2H), 7.68 – 7.55 (m, 2H), 7.12 (td, *J* = 5.1, 3.2 Hz, 1H), 6.66 – 6.50 (m, 2H), 5.15 (s, 1H), 3.82 – 3.67 (m, 4H), 3.00 (s, 6H), 2.61 (ddd, *J* = 10.2, 5.9, 3.2 Hz, 2H), 2.41 (ddd, *J* = 11.1, 6.0, 3.2 Hz, 2H). $^{13}C\{^{1}H\}$ NMR (101 MHz, $CDCl_3$) δ 194.1, 156.8, 153.5, 149.2, 136.7, 131.4, 124.4, 123.7, 122.7, 110.6, 77.3, 66.9, 52.2, 39.9. HRMS (ESI) calcd. for $C_{19}H_{23}N_3O_2$ $[M+H]^+$: 326.1863, found: 326.1869.

1-(4-fluorophenyl)-2-morpholino-2-(pyridin-2-yl)ethan-1-one (3ag)

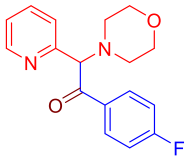

Compound **3ag** was prepared following the general procedure, purified by column chromatography using EtOAc/hexanes (1:2, v/v), and isolated as a yellow oil, 41.4 mg, 69%, ^{1}H NMR (400 MHz, $CDCl_3$) δ 8.55 (ddd, J = 4.9, 1.9, 1.0 Hz, 1H), 8.27 – 8.05 (m, 2H), 7.65 (td, J = 7.7, 1.8 Hz, 1H), 7.54 (dt, J = 7.9, 1.1 Hz, 1H), 7.17 (ddd, J = 7.5, 4.9, 1.3 Hz, 1H), 7.12 – 7.02 (m, 2H), 5.18 (s, 1H), 3.83 – 3.67 (m, 4H), 2.67 – 2.53 (m, 2H), 2.46 (dddd, J = 11.1, 6.6, 3.7, 1.0 Hz, 2H). ^{13}C{^{1}H} NMR (101 MHz, $CDCl_3$) δ 195.1, 167.1 (d, J^{1}_{C-F} = 256.5 Hz), 155.6, 149.6, 136.9, 132.7 (d, J^{4}_{C-F} = 3.0 Hz), 131.9 (d, J^{3}_{C-F} = 9.1 Hz), 123.9, 123.1, 115.7 (d, J^{2}_{C-F} = 22.2 Hz), 78.1, 66.9, 52.0. ^{19}F NMR (376 MHz, $CDCl_3$) δ -104.5. HRMS (ESI) calcd. for $C_{17}H_{17}FN_2O_2$ [M+H]$^+$: 301.1347, found: 301.1342.

1-(4-chlorophenyl)-2-morpholino-2-(pyridin-2-yl)ethan-1-one (3ah)

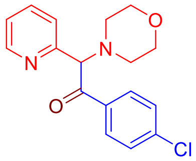

Compound **3ah** was prepared following the general procedure, purified by column chromatography using EtOAc/hexanes (1:2, v/v), and isolated as a yellow oil, 48.8 mg, 77%, ^{1}H NMR (500 MHz, $CDCl_3$) δ 8.55 (dd, J = 4.8, 2.0 Hz, 1H), 8.15 – 7.98 (m, 2H), 7.65 (td, J = 7.7, 2.1 Hz, 1H), 7.53 (d, J = 7.9 Hz, 1H), 7.36 (dd, J = 8.9, 2.1 Hz, 2H), 7.18 (ddd, J = 7.5, 4.9, 1.4 Hz, 1H), 5.18 (s, 1H), 3.82 – 3.71 (m, 4H), 2.68 – 2.55 (m, 2H), 2.48 (dt, J = 11.1, 4.3 Hz, 2H). ^{13}C{^{1}H} NMR (126 MHz, $CDCl_3$) δ 195.5, 155.4, 149.6, 139.8, 136.9, 134.6, 130.5, 128.8, 124.0, 123.1, 78.1, 66.9, 51.9. HRMS (ESI) calcd. for $C_{17}H_{17}ClN_2O_2$ [M+H]$^+$: 317.1051, found: 317.1057.

2-morpholino-2-(pyridin-2-yl)-1-(4-(trifluoromethoxy)phenyl)ethan-1-one (3ai)

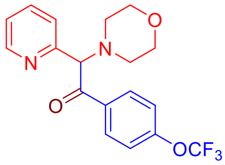

Compound **3ai** was prepared following the general procedure, purified by column chromatography using EtOAc/hexanes (1:2, v/v), and isolated as a brown oil, 41.8 mg, 57%, ^{1}H NMR (400 MHz, $CDCl_3$) 8.56 (ddd, J = 4.9, 1.8, 0.9 Hz, 1H), 8.21 – 8.17 (m, 2H), 7.67 (td, J = 7.7, 1.8 Hz, 1H), 7.54 (dt, J = 7.9, 1.1 Hz, 1H), 7.24 – 7.17 (m, 3H), 5.19 (s, 1H), 3.77 – 3.72 (m, 4H), 2.64 – 2.56 (m, 2H), 2.48 (dtd, J = 10.8, 4.0, 1.9 Hz, 2H). ^{13}C{^{1}H} NMR (101 MHz, $CDCl_3$) δ 195.2, 155.3, 149.6, 137.0, 134.4, 131.2, 129.6, 124.1 (q, J^{1}_{C-F} = 262.6 Hz), 124.0, 123.2, 120.1, 78.2, 66.9, 51.9. ^{19}F NMR (376 MHz, $CDCl_3$) δ -57.58. HRMS (ESI) calcd. for $C_{18}H_{17}F_3N_2O_3$ [M+H]$^+$: 367.1264, found: 367.1260.

2-morpholino-2-(pyridin-2-yl)-1-(4-(pyrrolidin-1-yl)phenyl)ethan-1-one (3aj)

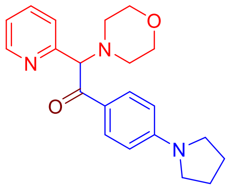

Compound **3aj** was prepared following the general procedure, purified by column chromatography using EtOAc/hexanes (1:1, v/v), and isolated as a yellow oil, 49.2 mg, 70%, ^{1}H NMR (500 MHz, $CDCl_3$) δ 8.52 (d, J = 4.9 Hz, 1H), 8.05 (d, J = 8.6 Hz, 2H), 7.61 (d, J = 8.4 Hz, 2H), 7.12 (s, 1H), 6.44 (d, J = 8.6 Hz, 2H), 5.16 (s, 1H), 3.82 – 3.64 (m, 4H), 3.30 (d, J = 6.2 Hz, 4H), 2.62 (t, J = 9.3 Hz, 2H), 2.42 (t, J = 8.3 Hz, 2H), 1.97 (q, J = 3.6 Hz, 4H). ^{13}C{^{1}H} NMR (126 MHz, $CDCl_3$) δ 193.9, 156.8, 151.1, 149.2, 136.8, 131.5, 123.9, 123.7, 122.7, 110.7, 77.2, 66.9, 52.2, 47.5, 25.4. HRMS (ESI) calcd. for $C_{21}H_{25}N_3O_2$ [M+H]$^+$: 352.2020, found: 352.2023.

1-(4-(1H-pyrrol-1-yl)phenyl)-2-morpholino-2-(pyridin-2-yl)ethan-1-one (3ak)

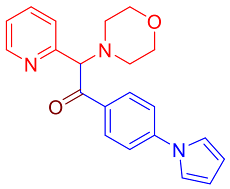

Compound **3ak** was prepared following the general procedure, purified by column chromatography using EtOAc/hexanes (1:2, v/v), and isolated as a brown oil, 45.2 mg, 65%, ^{1}H NMR (400 MHz, $CDCl_3$) δ 8.57 (ddd, J = 4.9, 1.9, 0.9 Hz, 1H), 8.27 – 8.16 (m, 2H), 7.67 (td, J = 7.7, 1.9 Hz, 1H), 7.58 (dt, J = 7.9, 1.1 Hz, 1H), 7.43 – 7.37 (m, 2H), 7.19 (ddd, J = 7.4, 4.9, 1.3 Hz, 1H), 7.14 – 7.10 (m, 2H), 6.36 (t, J = 2.2 Hz, 2H), 5.23 (s, 1H), 3.81 – 3.72 (m, 4H), 2.68 – 2.57 (m, 2H), 2.54 – 2.45 (m, 2H). ^{13}C{^{1}H} NMR (101 MHz, $CDCl_3$) δ 195.3, 155.7, 149.6, 144.1, 137.0, 133.0, 131.0, 123.9, 123.1, 119.2, 118.9, 111.7, 78.0, 66.9, 52.0. HRMS (ESI) calcd. for $C_{21}H_{21}N_3O_2$ [M+H]$^+$: 348.1707, found: 348.1701.

2-morpholino-2-(pyridin-2-yl)-1-(m-tolyl)ethan-1-one (3al)

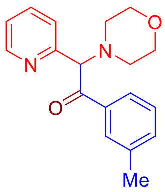

Compound **3al** was prepared following the general procedure, purified by column chromatography using EtOAc/hexanes (1:2, v/v), and isolated as a yellow oil, 49.8 mg, 84%, ^{1}H NMR (400 MHz, $CDCl_3$) δ 8.56 (ddd, J = 4.9, 1.7, 0.9 Hz, 1H), 8.04 – 7.78 (m, 2H), 7.65 (td, J = 7.7, 1.9 Hz, 1H), 7.57 (dt, J = 7.9, 1.2 Hz, 1H), 7.30 (td, J = 7.5, 3.6 Hz, 2H), 7.17 (ddd, J = 7.5, 4.9, 1.3 Hz, 1H), 5.27 (s, 1H), 3.84 – 3.68 (m, 4H), 2.62 (ddd, J = 10.0, 5.8, 3.5 Hz, 2H), 2.55 – 2.44 (m, 2H), 2.36 (s, 3H). ^{13}C{^{1}H} NMR (101 MHz, $CDCl_3$) δ 196.9, 155.8, 149.5, 138.4, 136.9, 136.5, 134.1, 129.3, 128.4, 126.3, 123.9, 123.0, 77.6, 66.9, 52.0, 21.3. HRMS (ESI) calcd. for $C_{18}H_{20}N_2O_2$ [M+H]$^+$: 297.1598, found: 297.1603.

1-(3-chlorophenyl)-2-morpholino-2-(pyridin-2-yl)ethan-1-one (3am)

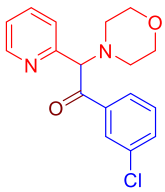

Compound **3am** was prepared following the general procedure, purified by column chromatography using EtOAc/hexanes (1:2, v/v), and isolated as a yellow oil, 48.8 mg, 77%, ^{1}H NMR (400 MHz, $CDCl_3$) δ 8.54 (dt, J = 5.0, 1.3 Hz, 1H), 8.14 – 7.87 (m, 2H), 7.65 (td, J = 7.7, 1.8 Hz, 1H), 7.52 (dd, J = 7.9, 1.2 Hz, 1H), 7.44 (ddd, J = 8.0, 2.2, 1.0 Hz, 1H), 7.31 (t, J = 7.9 Hz, 1H), 7.17 (ddd, J = 7.5, 4.9, 1.2 Hz, 1H), 5.18 (s, 1H), 3.73 (dt, J = 6.0, 3.1 Hz, 4H), 2.63 – 2.53 (m, 2H), 2.53 – 2.43 (m, 2H). ^{13}C{^{1}H} NMR (101 MHz, $CDCl_3$) δ 195.6, 155.2, 149.7, 137.8, 137.0, 134.9, 133.2, 129.8, 129.0, 127.1, 124.0, 123.2, 77.9, 66.9, 51.8. HRMS (ESI) calcd. for $C_{17}H_{17}ClN_2O_2$ [M+H]$^+$: 317.1051, found: 317.1057.

2-morpholino-2-(pyridin-2-yl)-1-(3-(trifluoromethyl)phenyl)ethan-1-one (3an)

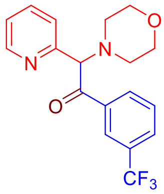

Compound **3an** was prepared following the general procedure, purified by column chromatography using EtOAc/hexanes (1:2, v/v), and isolated as a yellow oil, 42.7 mg, 61%, ^{1}H NMR (400 MHz, $CDCl_3$) 8.56 (ddd, J = 4.9, 1.8, 0.9 Hz, 1H), 8.40 (d, J = 1.9 Hz, 1H), 8.33 (dt, J = 8.1, 1.5 Hz, 1H), 7.77 – 7.72 (m, 1H), 7.67 (td, J = 7.7, 1.8 Hz, 1H), 7.56 – 7.51 (m, 2H), 7.20 (ddd, J = 7.5, 4.9, 1.2 Hz, 1H), 5.22 (s, 1H), 3.75 (ddd, J = 5.9, 3.6, 2.3 Hz, 4H), 2.61 (ddd, J = 9.5, 7.2, 4.0 Hz, 2H), 2.55 – 2.47 (m, 2H). ^{13}C{^{1}H} NMR (101 MHz, $CDCl_3$) δ 195.5, 155.1, 149.7, 137.0, 136.7, 132.3, 131.6 (q, J^2_{C-F} = 33.3 Hz), 129.6 (q, J^3_{C-F} = 4.0 Hz), 129.1, 127.7 (q, J^1_{C-F} = 273.7 Hz), 126.0 (q, J^3_{C-F} = 4.0 Hz), 124.0, 123.2, 78.3, 66.9, 51.8. ^{19}F NMR (376 MHz, $CDCl_3$) δ -62.9. HRMS (ESI) calcd. for $C_{18}H_{17}F_3N_2O_2$ [M+H]$^+$: 351.1315, found: 351.1311.

1-(3,5-dimethylphenyl)-2-morpholino-2-(pyridin-2-yl)ethan-1-one (3ao)

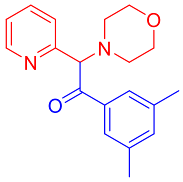

Compound **3ao** was prepared following the general procedure, purified by column chromatography using EtOAc/hexanes (1:2, v/v), and isolated as a yellow oil, 54.0 mg, 87%, ^{1}H NMR (500 MHz, $CDCl_3$) δ 8.57 – 8.52 (m, 1H), 7.69 (s, 2H), 7.64 (td, *J* = 7.7, 1.8 Hz, 1H), 7.57 (d, *J* = 7.9 Hz, 1H), 7.19 – 7.10 (m, 2H), 5.26 (s, 1H), 3.79 – 3.69 (m, 4H), 2.70 – 2.56 (m, 2H), 2.46 (ddt, *J* = 8.3, 5.8, 2.7 Hz, 2H), 2.31 (s, 6H). ^{13}C{^{1}H} NMR (126 MHz, $CDCl_3$) δ 197.1, 155.9, 149.5, 138.2, 136.9, 136.7, 135.1, 126.7, 123.9, 123.0, 77.5, 66.9, 52.0, 21.2. HRMS (ESI) calcd. for $C_{19}H_{22}N_2O_2$ [M+H]$^+$: 311.1754, found: 311.1743.

1-(3,5-di-tert-butylphenyl)-2-morpholino-2-(pyridin-2-yl)ethan-1-one (3ap)

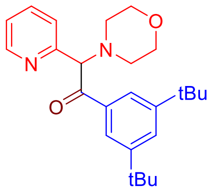

Compound **3ap** was prepared following the general procedure, purified by column chromatography using EtOAc/hexanes (1:2, v/v), and isolated as a yellow oil, 67.9 mg, 86%, ^{1}H NMR (400 MHz, $CDCl_3$) δ 8.57 (ddd, *J* = 4.9, 1.9, 1.0 Hz, 1H), 8.00 (d, *J* = 1.9 Hz, 2H), 7.63 (td, *J* = 7.7, 1.8 Hz, 1H), 7.59 – 7.51 (m, 2H), 7.16 (ddd, *J* = 7.4, 4.9, 1.3 Hz, 1H), 5.25 (s, 1H), 3.75 (dt, *J* = 5.8, 3.7 Hz, 4H), 2.70 – 2.60 (m, 2H), 2.49 (dd, *J* = 6.6, 4.0 Hz, 2H), 1.29 (s, 18H). ^{13}C{^{1}H} NMR (101 MHz, $CDCl_3$) δ 196.7, 156.0, 150.9, 149.4, 136.7, 135.6, 127.3, 123.8, 123.4, 122.8, 78.3, 66.9, 51.9, 34.9, 31.2. HRMS (ESI) calcd. for $C_{25}H_{34}N_2O_2$ [M+H]$^+$: 395.2693, found: 395.2687.

2-morpholino-1-(naphthalen-2-yl)-2-(pyridin-2-yl)ethan-1-one (3aq)

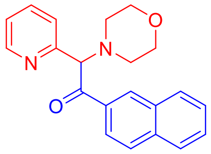

Compound **3aq** was prepared following the general procedure, purified by column chromatography using EtOAc/hexanes (1:2, v/v), and isolated as a brown oil, 42.5 mg, 64%, ^{1}H NMR (400 MHz, $CDCl_3$) δ 8.72 (d, *J* = 1.7 Hz, 1H), 8.56 (dt, *J* = 4.9, 1.4 Hz, 1H), 8.10 (dd, *J* = 8.7, 1.8 Hz, 1H), 7.95 (dd, *J* = 8.5, 1.6 Hz, 1H), 7.86 – 7.75 (m, 2H), 7.69 – 7.59 (m, 2H), 7.59 – 7.49 (m, 2H), 7.16 (ddd, *J* = 6.8, 4.9, 1.8 Hz, 1H), 5.43 (s, 1H), 3.85 – 3.71 (m, 4H), 2.67 (ddd, *J* = 9.9, 5.8, 3.4 Hz, 2H), 2.52 (ddd, *J* = 10.7, 6.0, 3.3 Hz, 2H). ^{13}C{^{1}H} NMR (101 MHz, $CDCl_3$) δ 196.7, 155.8, 149.6, 136.9, 135.6, 133.7, 132.4, 131.1, 129.9, 128.7, 128.4, 127.7, 126.7, 124.4, 123.9, 123.0, 77.8, 67.0, 52.1. HRMS (ESI) calcd. for $C_{21}H_{20}N_2O_2$ [M+H]$^+$: 333.1598, found: 333.1603.

1-(2,3-dihydrobenzo[b][1,4]dioxin-6-yl)-2-morpholino-2-(pyridin-2-yl)ethan-1-one (3ar)

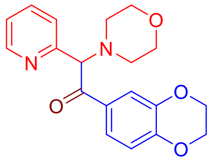

Compound **3ar** was prepared following the general procedure, purified by column chromatography using EtOAc/hexanes (1:2, v/v), and isolated as a yellow oil, 48.3 mg, 71%, ^{1}H NMR (500 MHz, $CDCl_3$) δ 8.55 (dd, *J* = 5.0, 1.9 Hz, 1H), 7.75 – 7.51 (m, 4H), 7.20 – 7.12 (m, 1H), 6.82 (dd, *J* = 9.1, 2.5 Hz, 1H), 5.18 (s, 1H), 4.24 (ddd, *J* = 20.3, 6.0, 3.0 Hz, 4H), 3.83 – 3.62 (m, 4H), 2.69 – 2.32 (m, 4H). ^{13}C{^{1}H} NMR (126 MHz, $CDCl_3$) δ 194.9, 155.8, 149.5, 148.3, 143.3, 136.9, 130.1, 123.9, 123.3, 123.0, 118.5, 117.1, 77.4, 66.9, 64.7, 64.0, 52.0. HRMS (ESI) calcd. for $C_{19}H_{20}N_2O_4$ [M+H]$^+$: 341.1496, found: 341.1501

2-morpholino-2-(pyridin-2-yl)-1-(quinolin-6-yl)ethan-1-one (3as)

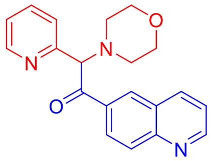

Compound **3as** was prepared following the general procedure, purified by column chromatography using EtOAc/hexanes (1:1, v/v), and isolated as a brown oil, 33.3 mg, 50%, ^{1}H NMR (400 MHz, $CDCl_3$) δ 8.96 (dd, *J* = 4.2, 1.8 Hz, 1H), 8.70 (d, *J* = 1.9 Hz, 1H), 8.55 (dt, *J* = 5.0, 1.3 Hz, 1H), 8.33 (dd, *J* = 8.9, 2.0 Hz, 1H), 8.26 (dd, *J* = 8.3, 1.7 Hz, 1H), 8.07 (d, *J* = 8.9 Hz, 1H), 7.67 – 7.56 (m, 2H), 7.43 (dd, *J* = 8.4, 4.3 Hz, 1H), 7.16 (ddd, *J* = 7.4, 4.9, 1.4 Hz, 1H), 5.39 (s, 1H), 3.76 (dt, *J* = 5.7, 3.7 Hz, 4H), 2.71 – 2.60 (m, 2H), 2.51 (ddd, *J* = 10.8, 5.9, 3.4 Hz, 2H). ^{13}C{^{1}H} NMR (101 MHz, $CDCl_3$) δ 196.2, 155.5, 152.8, 150.1, 149.6, 137.8, 137.0, 134.1, 130.9, 129.9, 128.2, 127.4, 124.0, 123.2, 121.9, 78.0, 66.9, 52.0. HRMS (ESI) calcd. for $C_{20}H_{19}N_3O_2$ $[M+H]^+$: 334.1550, found: 334.1556.

2-morpholino-2-(pyridin-2-yl)-1-(quinolin-6-yl)ethan-1-one (3at)

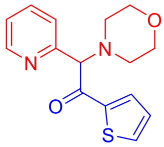

Compound **3at** was prepared following the general procedure, purified by column chromatography using EtOAc/hexanes (1:2, v/v), and isolated as a yellow oil, 31.7 mg, 55%, ^{1}H NMR (400 MHz, $CDCl_3$) δ 8.56 (d, *J* = 4.9 Hz, 1H), 8.03 (d, *J* = 3.8 Hz, 1H), 7.73 – 7.58 (m, 3H), 7.23 – 7.16 (m, 1H), 7.10 (t, *J* = 4.5 Hz, 1H), 5.01 (s, 1H), 3.76 (p, *J* = 3.0 Hz, 4H), 2.63 (dt, *J* = 9.9, 4.5 Hz, 2H), 2.48 (dt, *J* = 10.7, 4.6 Hz, 2H). ^{13}C{^{1}H} NMR (101 MHz, $CDCl_3$) δ 189.7, 155.6, 149.5, 143.0, 136.9, 134.7, 133.8, 128.2, 123.9, 123.1, 79.4, 66.8, 52.0. HRMS (ESI) calcd. for $C_{15}H_{16}N_2O_2S$ $[M+H]^+$: 289.1005, found: 289.1012.

7. References:

1. B. S. Kim; J. Jimenez; F. Gao; P. J. Walsh, Palladium-Catalyzed Benzylic C–H Arylation of Azaarylmethylamines. *Org. Lett.* **2015,** *17*, 5788-5791.
2. G. Gao; Y. Fu; M. Li; B. Wang; B. Zheng; S. Hou; P. J. Walsh, Arylation of Azaarylmethylamines with Aryl Chlorides and a NiBr2/NIXANTPHOS-based Catalyst. *Adv. Synth. Catal.* **2017,** *359*, 2890-2894.
3. N. Trongsiriwat; Y. Pu; Y. Nieves-Quinones; R. A. Shelp; M. C. Kozlowski; P. J. Walsh, Reactions of 2-Aryl-1, 3-Dithianes and [1.1. 1] Propellane. *Angew. Chem., Int. Ed.* **2019,** *58*, 13416-13420.
4. R. Lavernhe; E. J. Alexy; H. Zhang; B. M. Stoltz, Palladium-Catalyzed Enantioselective Decarboxylative Allylic Alkylation of Acyclic α-N-Pyrrolyl/Indolyl Ketones. *Org. Lett.* **2020,** *22*, 4272−4275.

8. Copies of ^1H and $^{13}C\{^1H\}$ NMR Spectra

^1H and $^{13}C\{^1H\}$ NMR spectra of compound **3aa** in $CDCl_3$

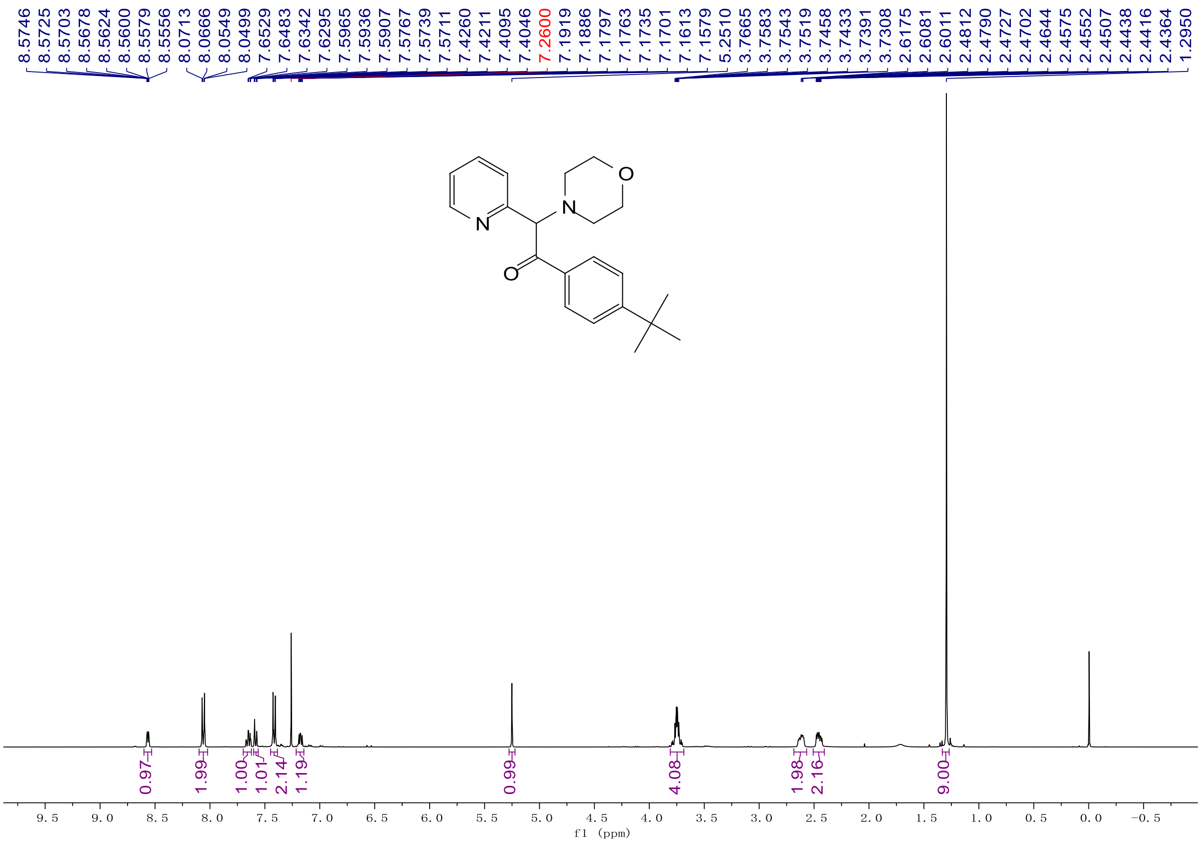

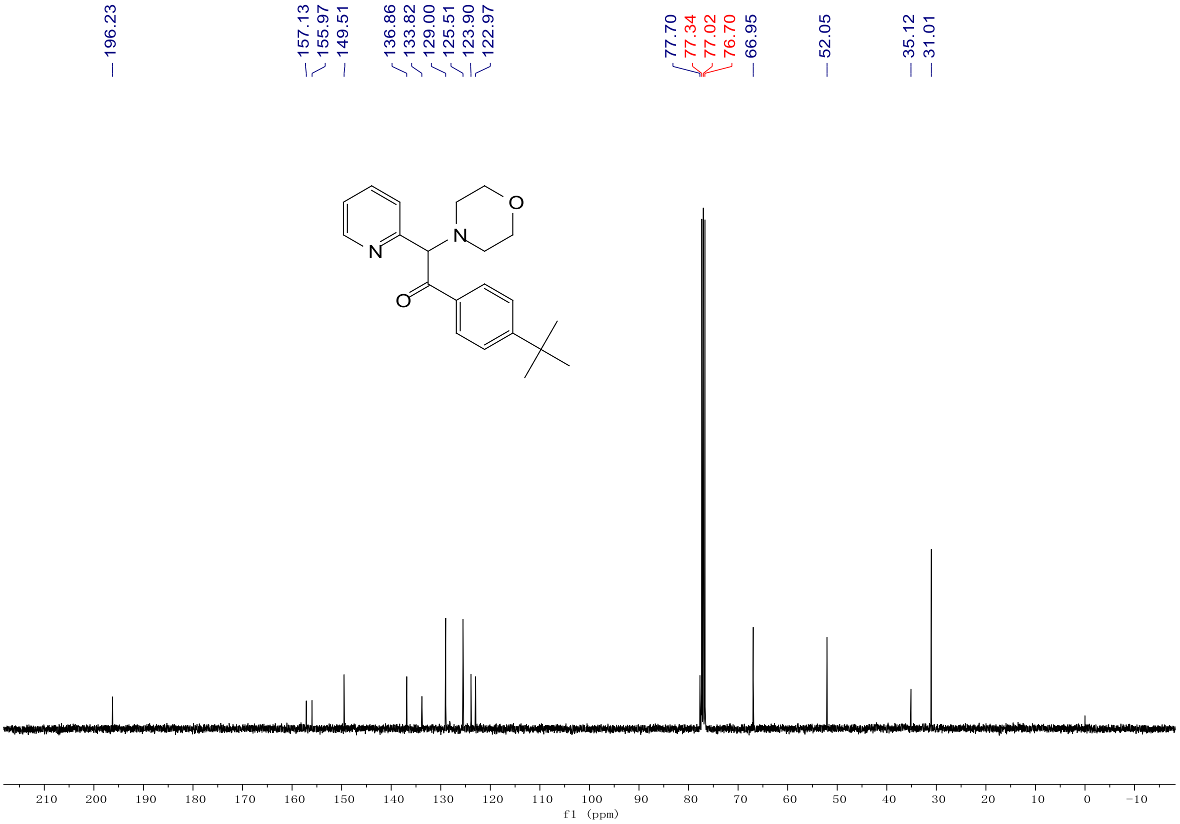

^{1}H and ^{13}C{^{1}H} NMR spectra of compound **3ba** in $CDCl_3$

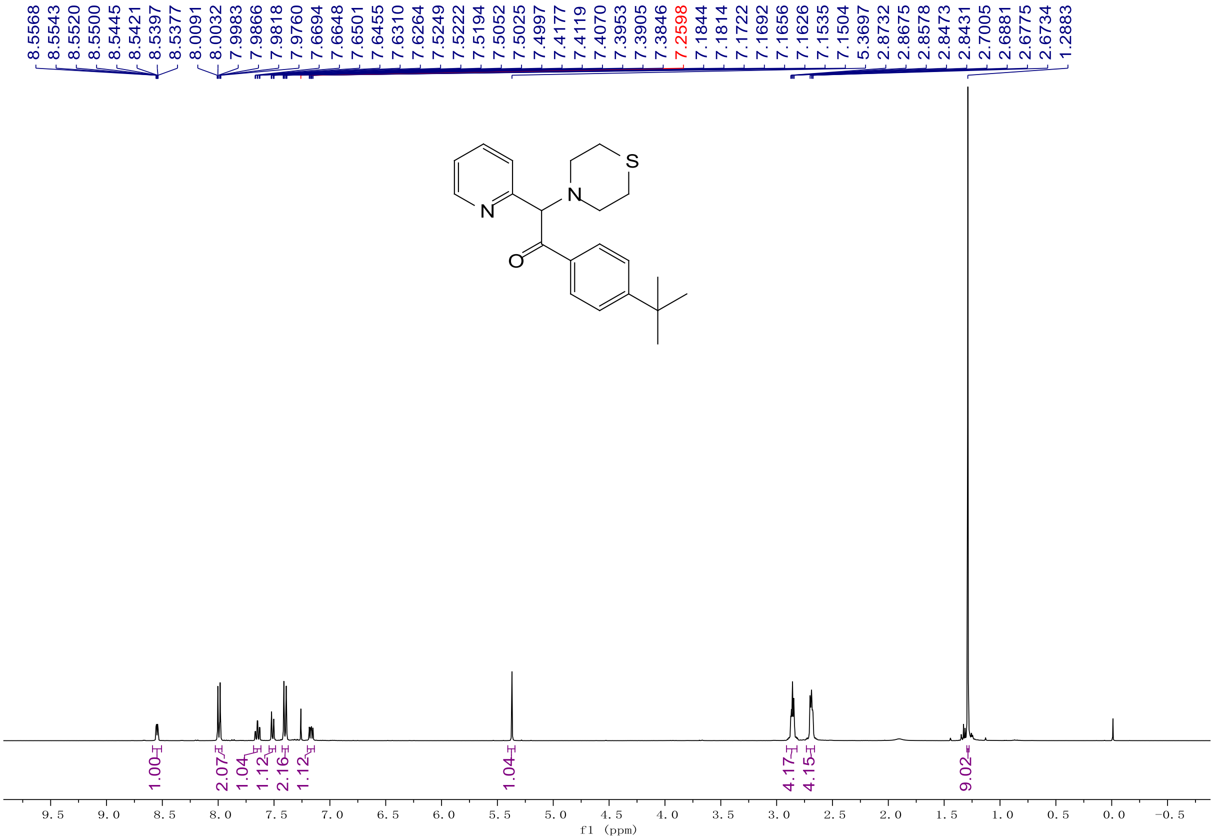

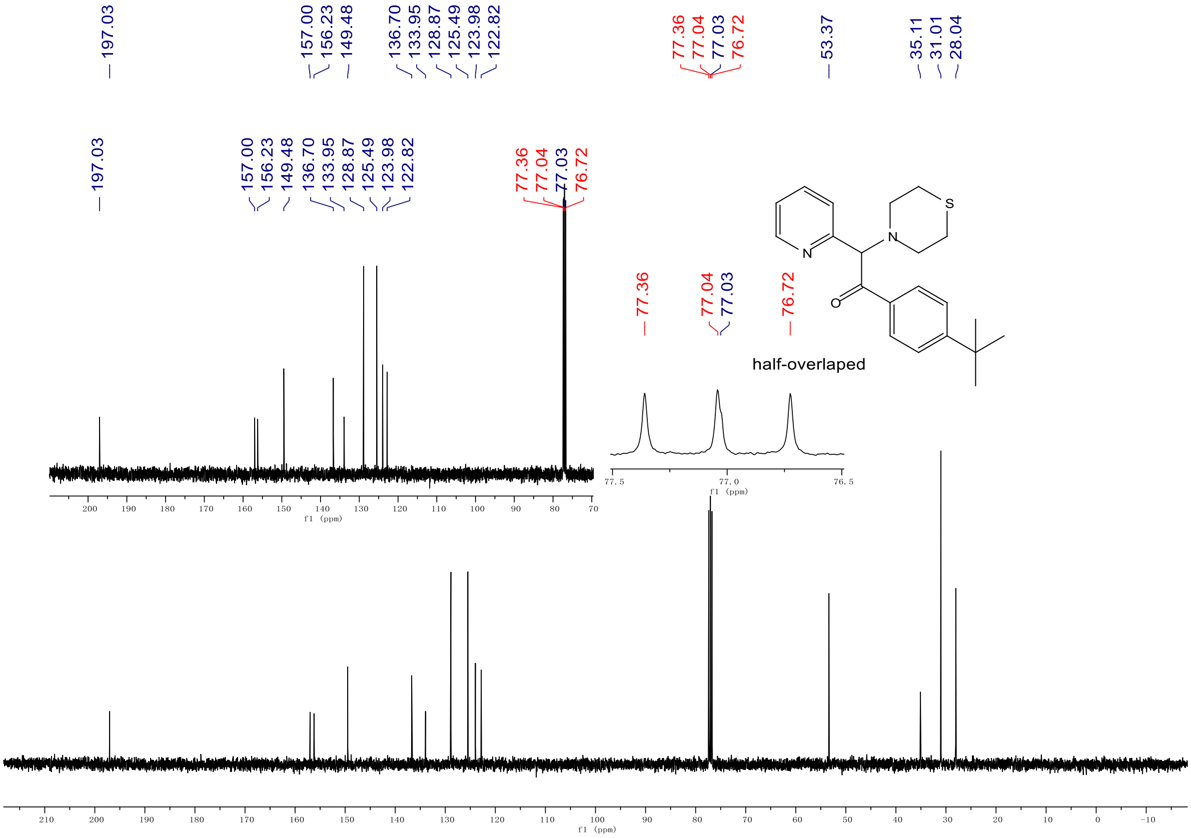

^{1}H and $^{13}C\{^{1}H\}$ NMR spectra of compound **3ca** in $CDCl_3$

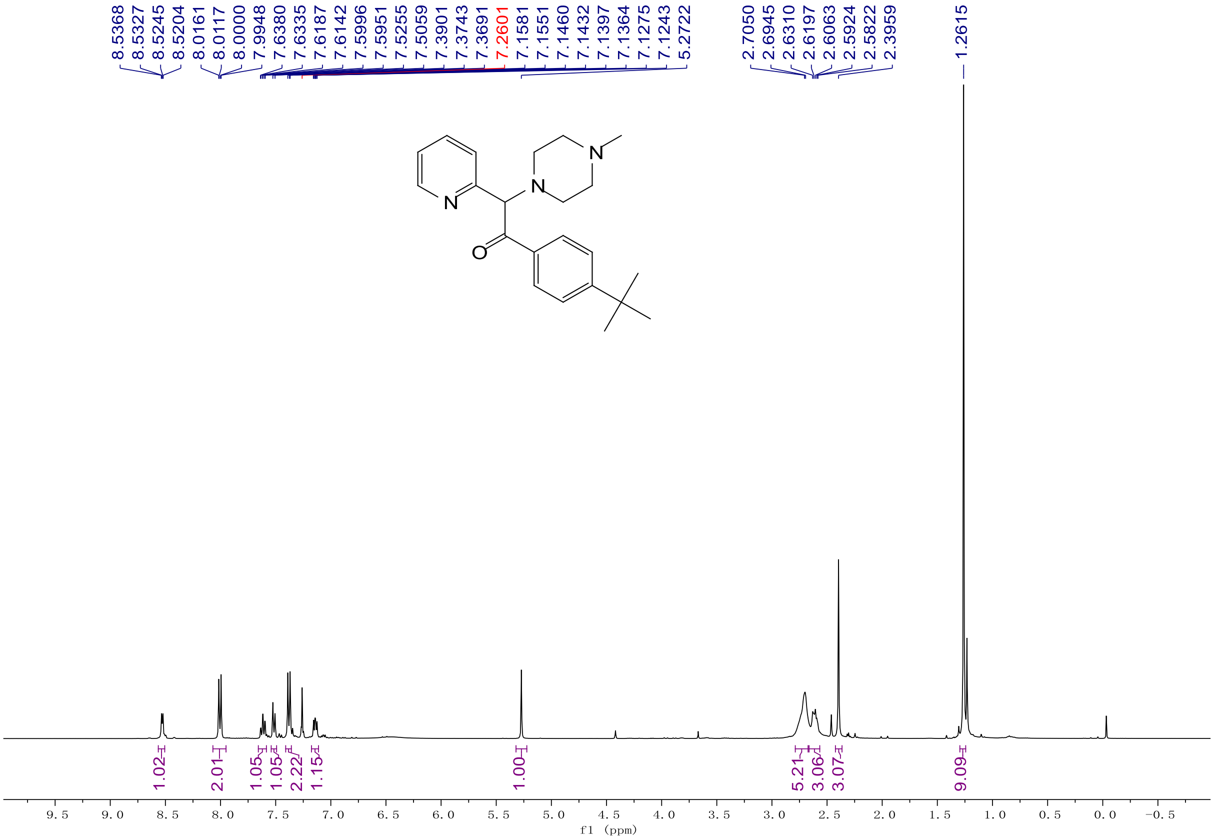

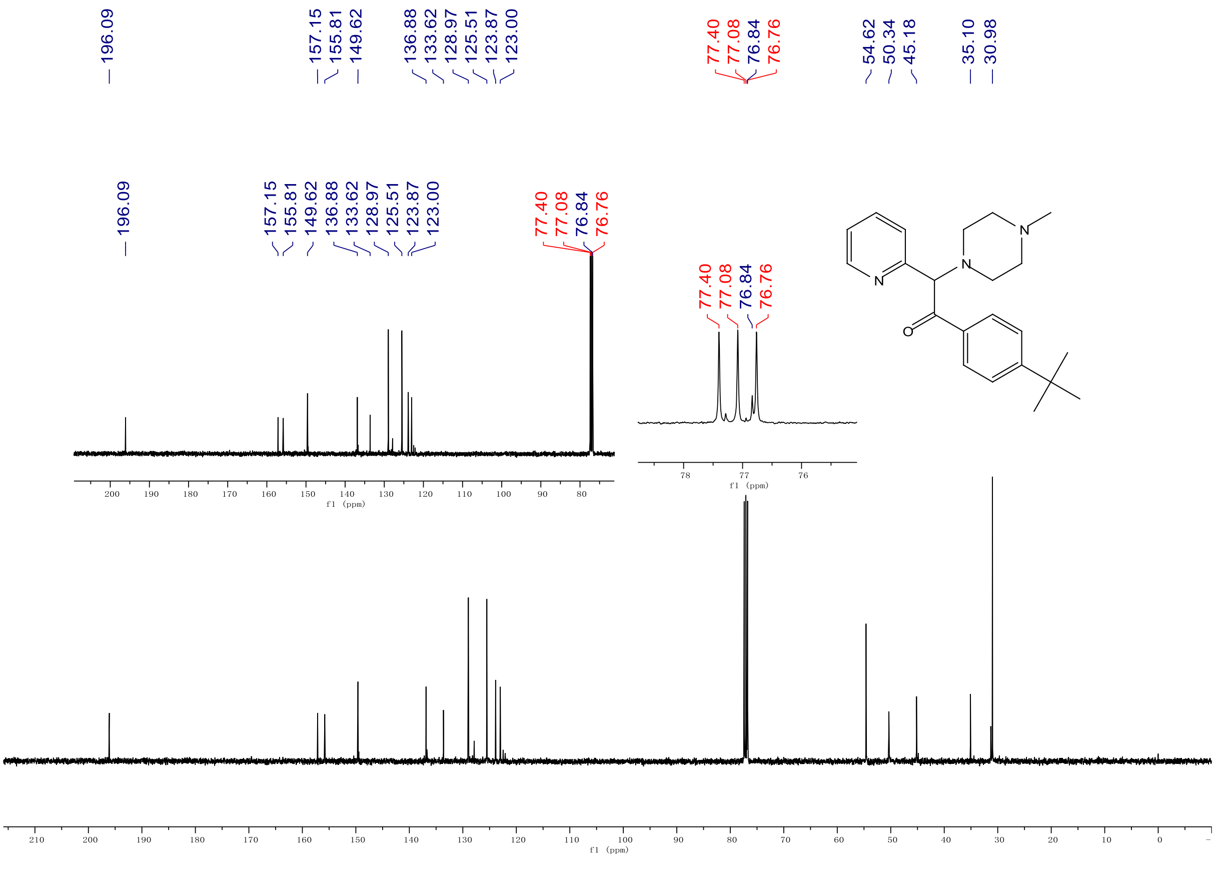

^{1}H and ^{13}C{^{1}H} NMR spectra of compound **3da** in $CDCl_3$

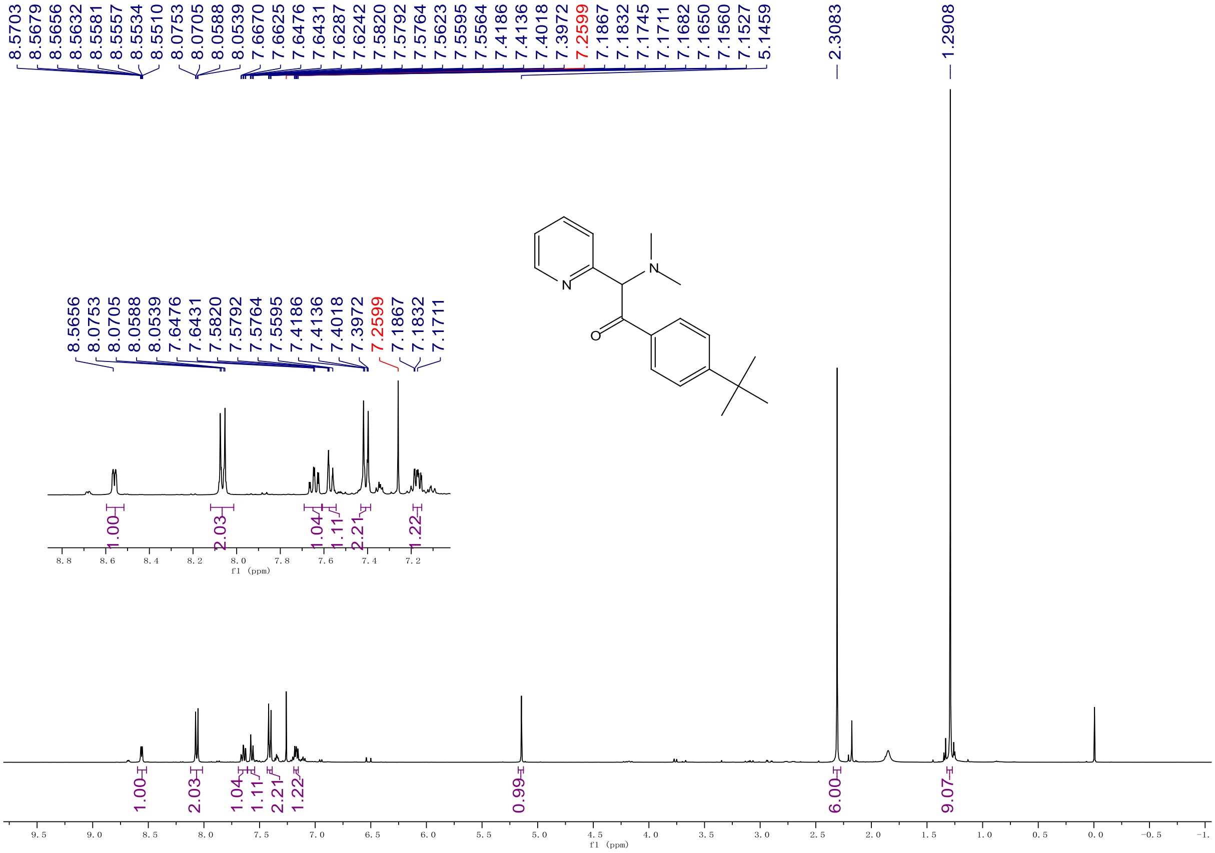

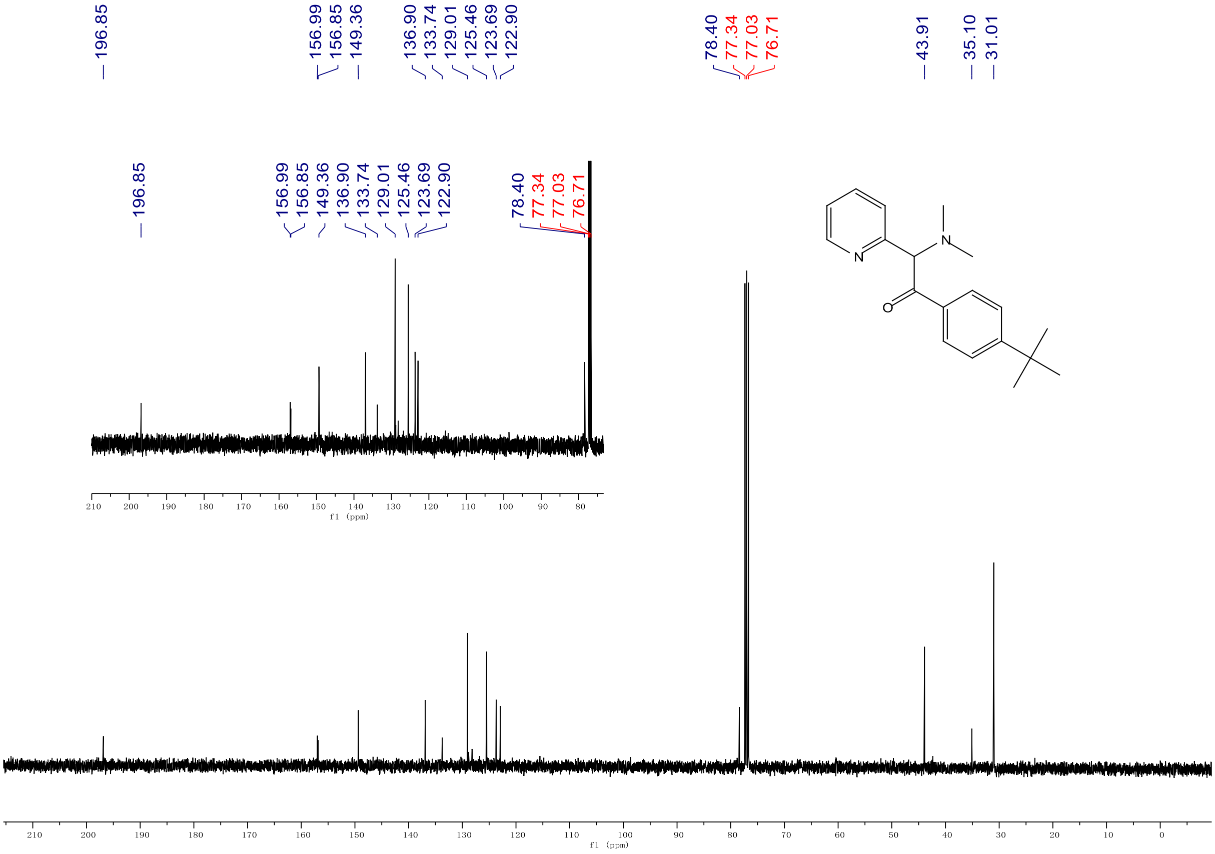

^{1}H and ^{13}C{^{1}H} NMR spectra of compound **3ea** in $CDCl_3$

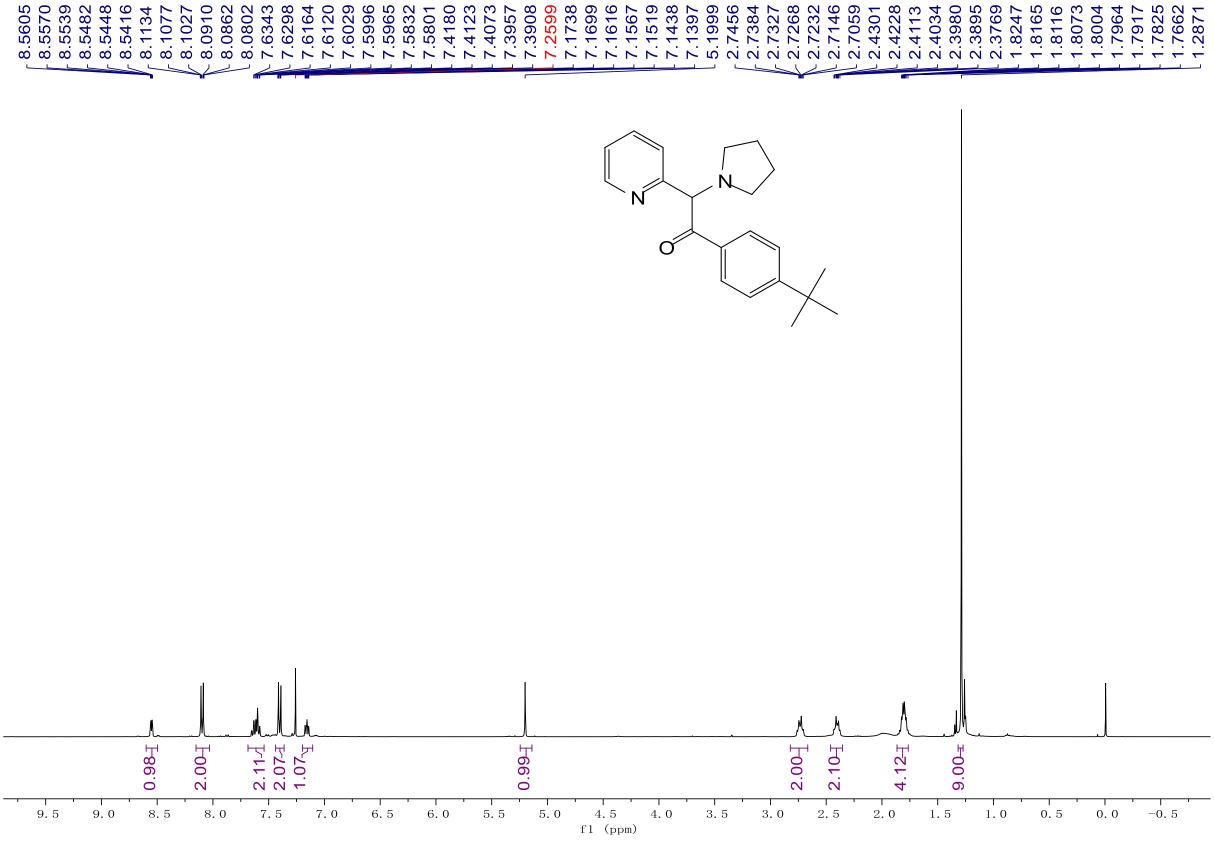

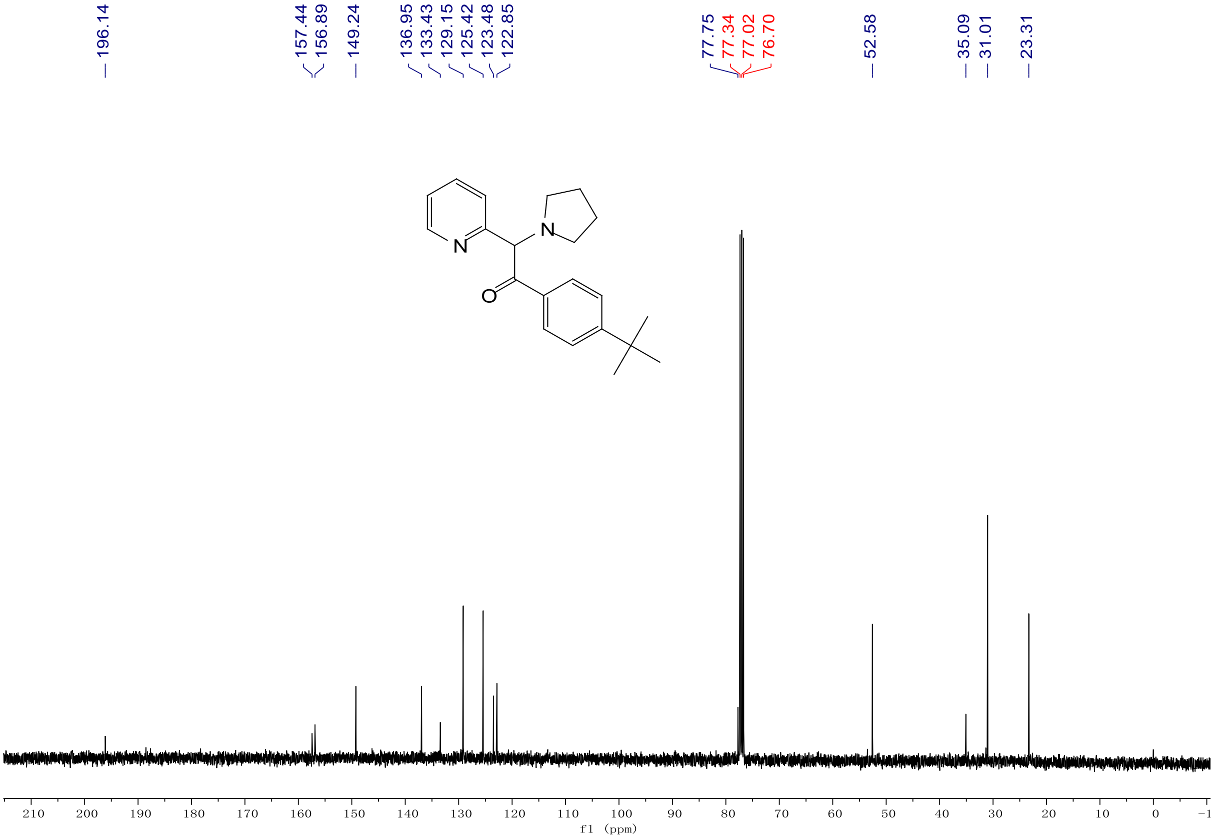

^{1}H and ^{13}C{^{1}H} NMR spectra of compound **3fa** in $CDCl_3$

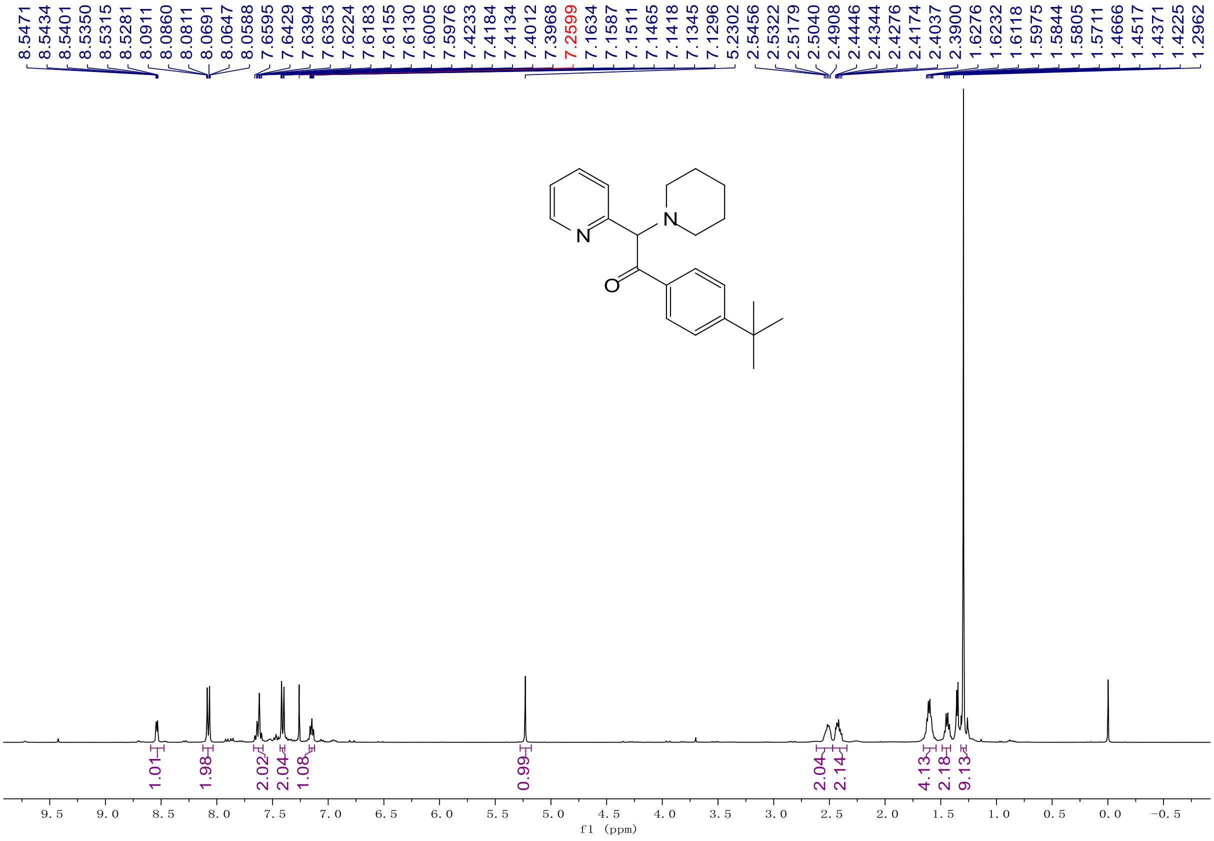

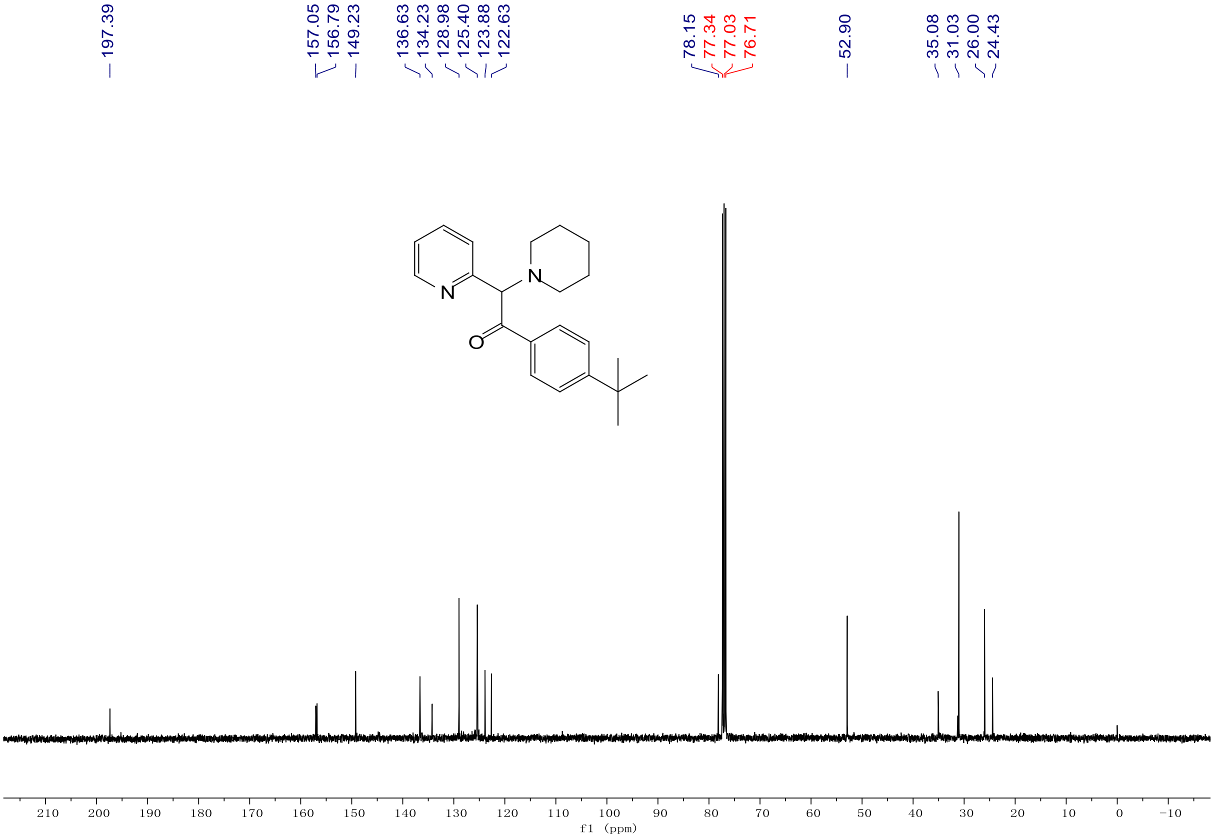

^{1}H and ^{13}C{^{1}H} NMR spectra of compound **3ga** in $CDCl_3$

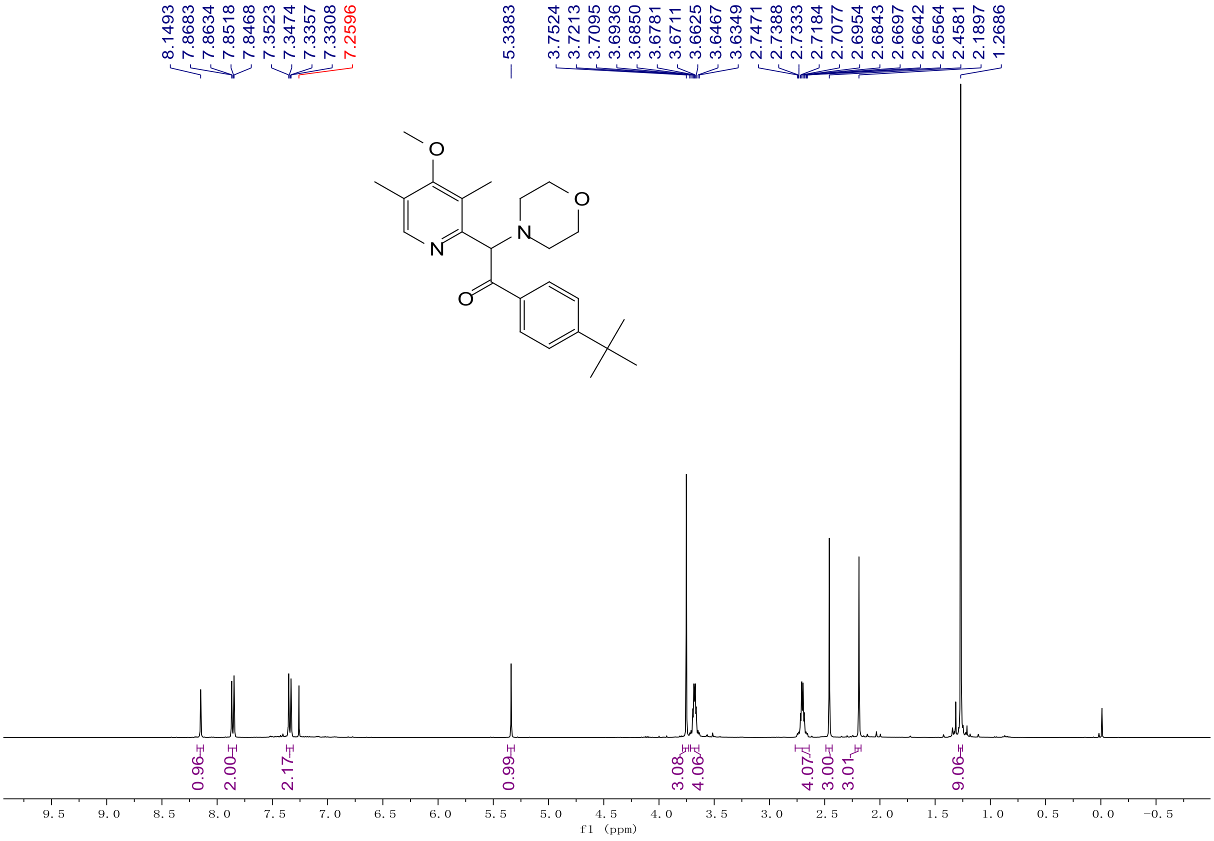

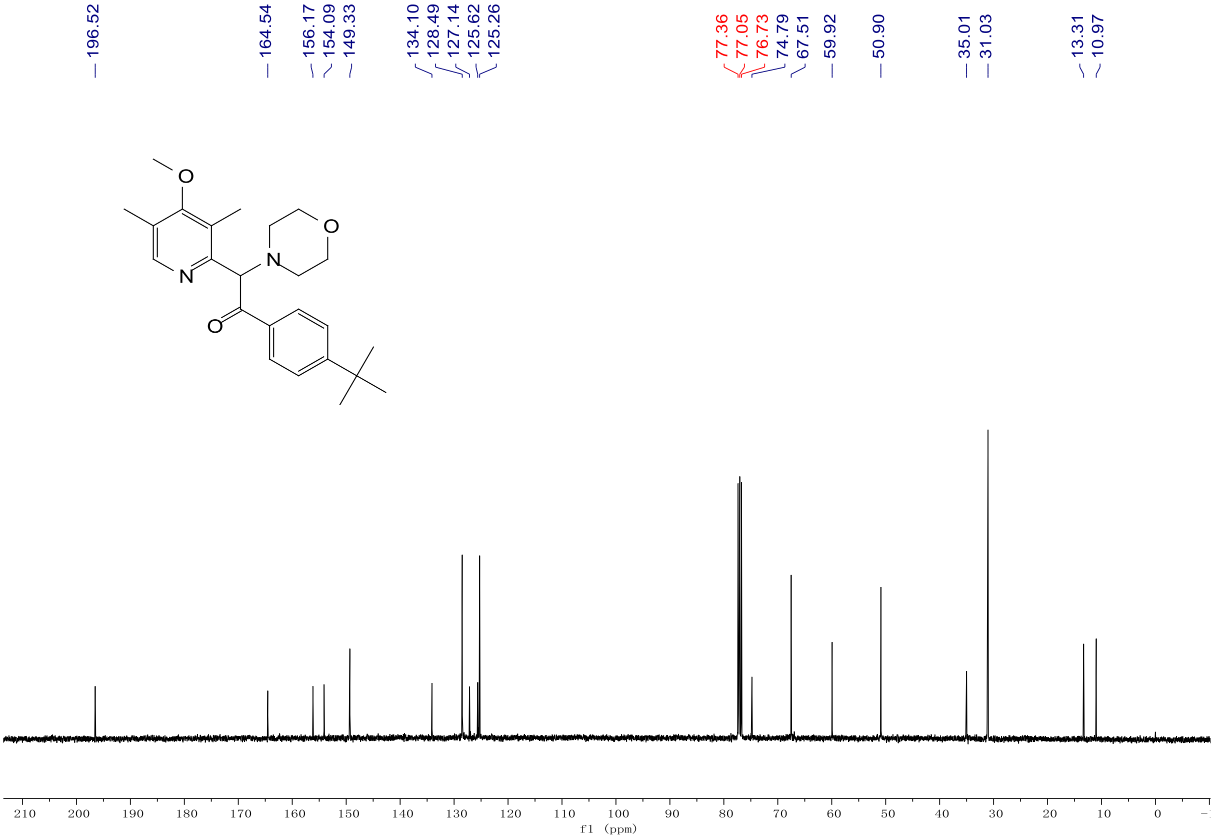

^{1}H and ^{13}C{^{1}H} NMR spectra of compound **3ha** in $CDCl_3$

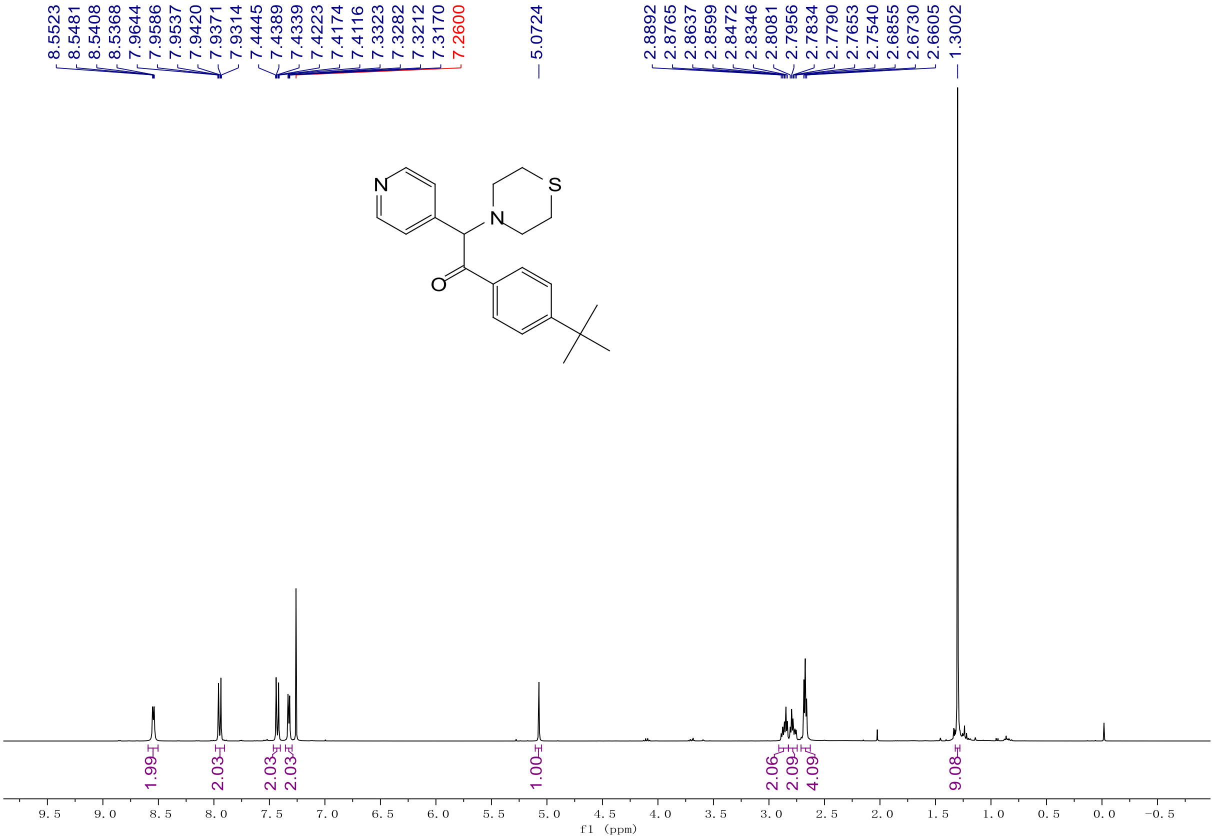

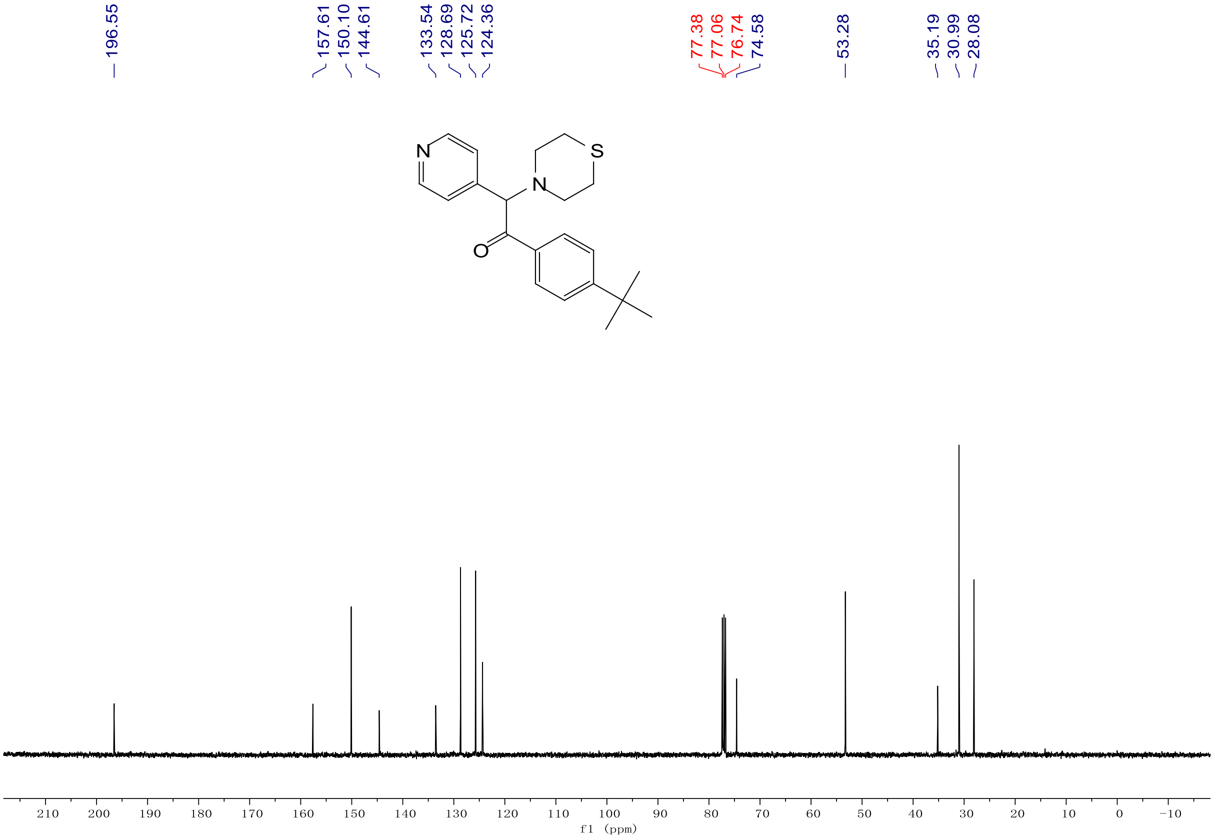

^{1}H and ^{13}C{^{1}H} NMR spectra of compound **3ia** in $CDCl_3$

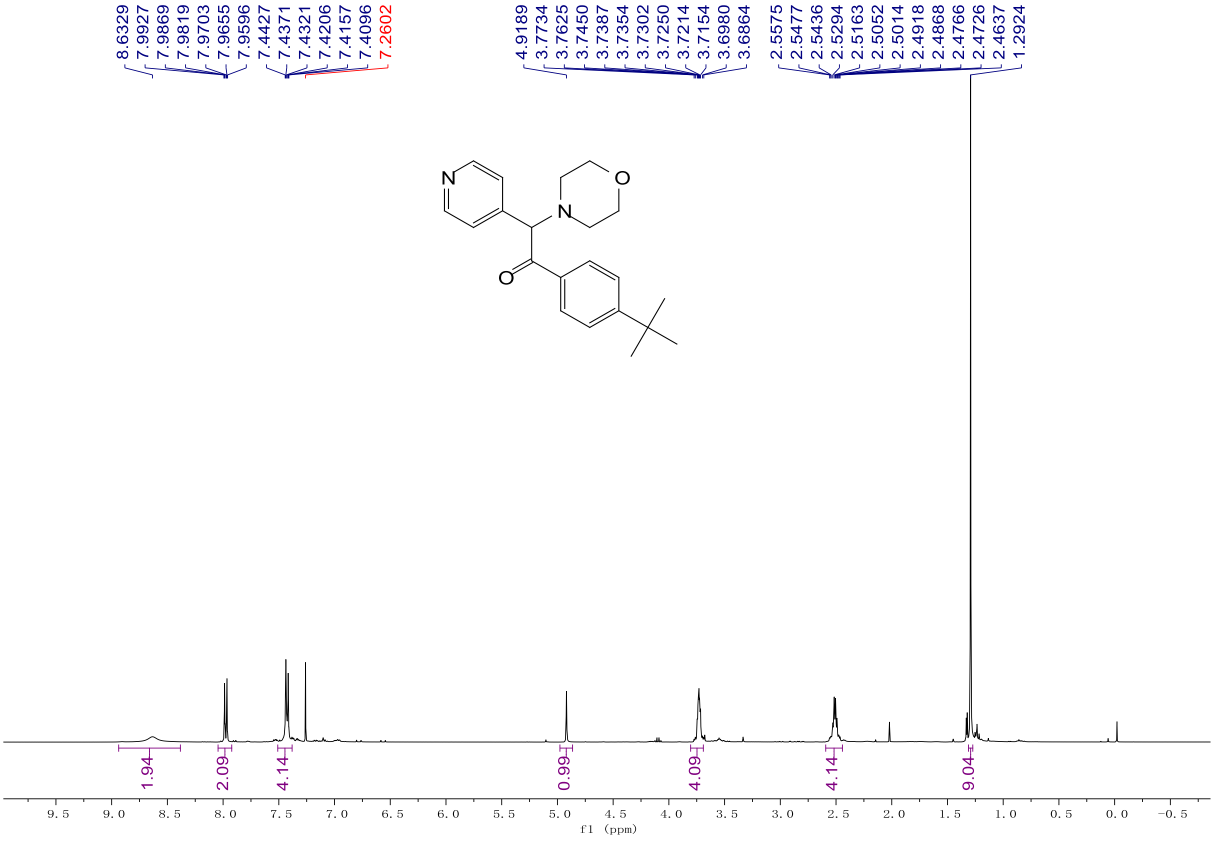

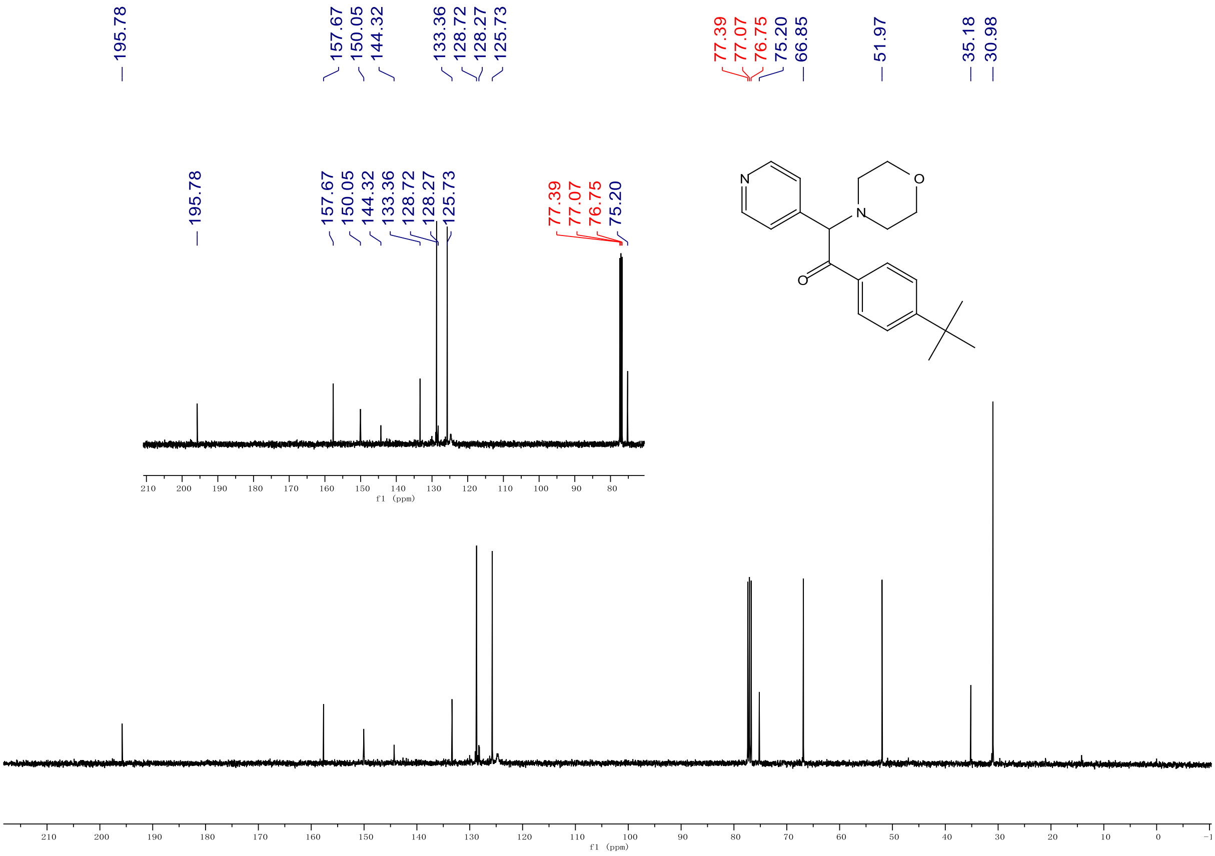

^{1}H and ^{13}C{^{1}H} NMR spectra of compound **3ja** in $CDCl_3$

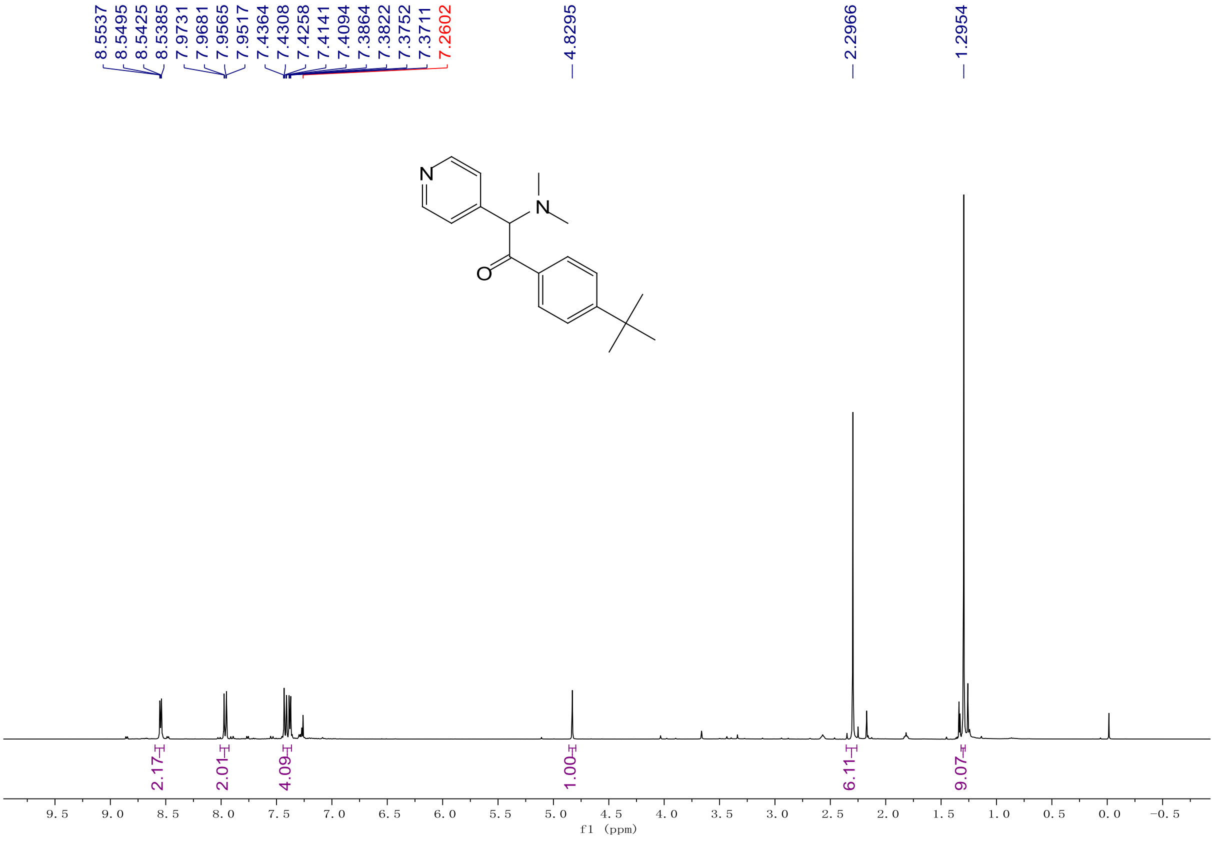

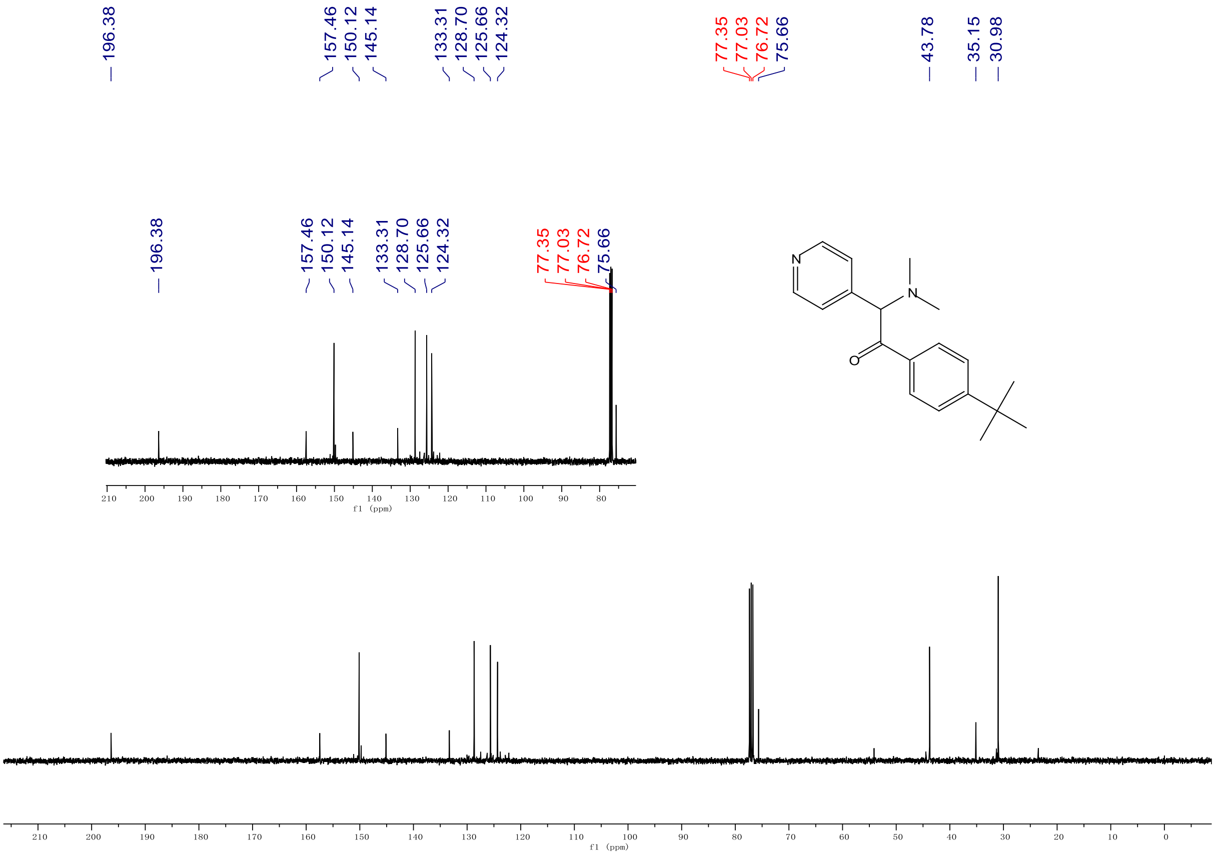

^{1}H and $^{13}C\{^{1}H\}$ NMR spectra of compound **3ka** in $CDCl_3$

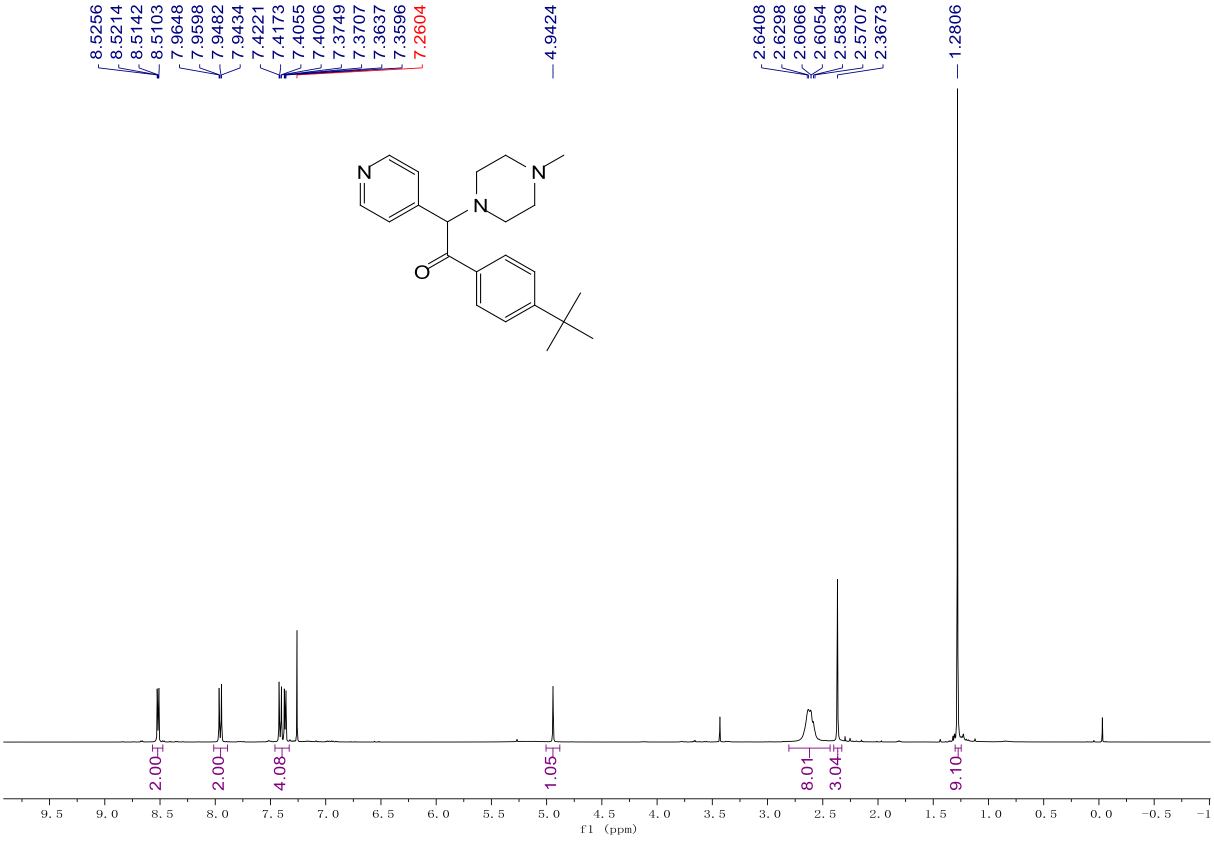

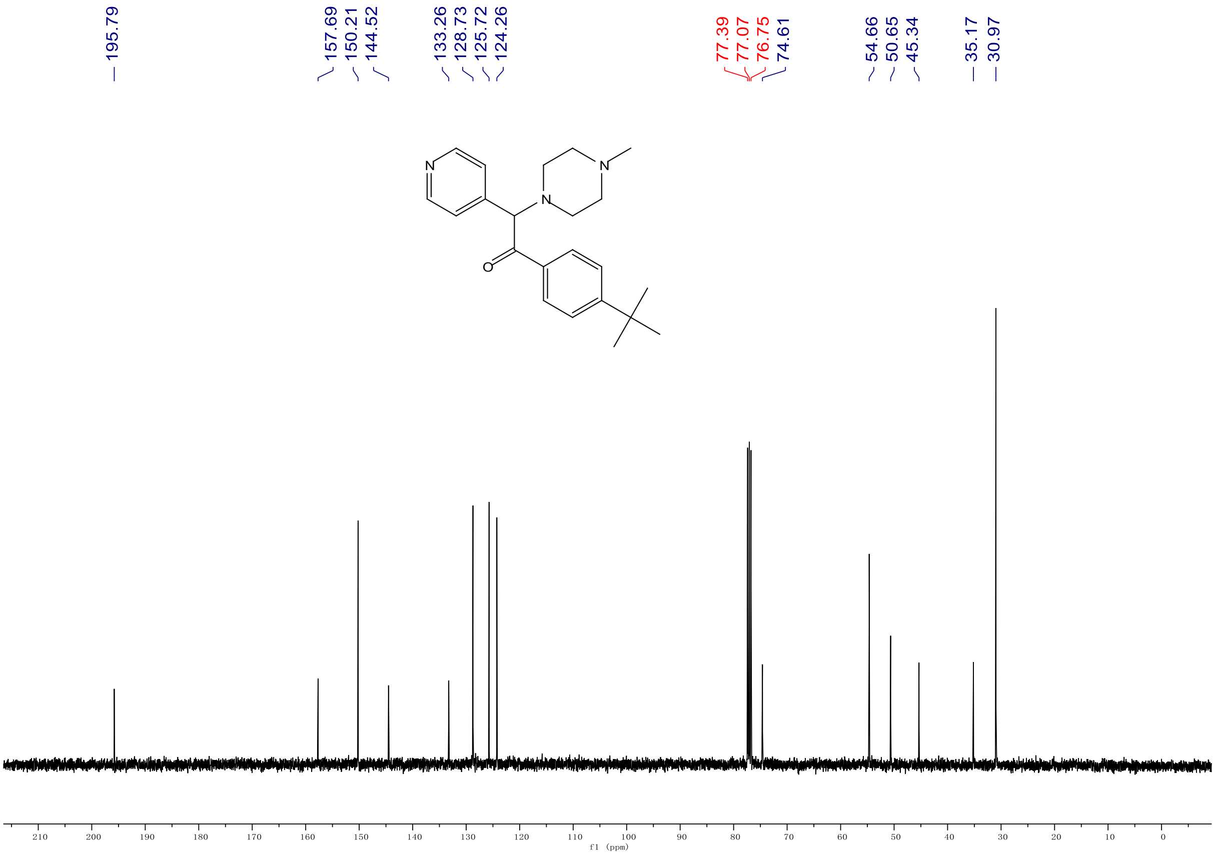

^{1}H and $^{13}C\{^{1}H\}$ NMR spectra of compound **3la** in C_6D_6

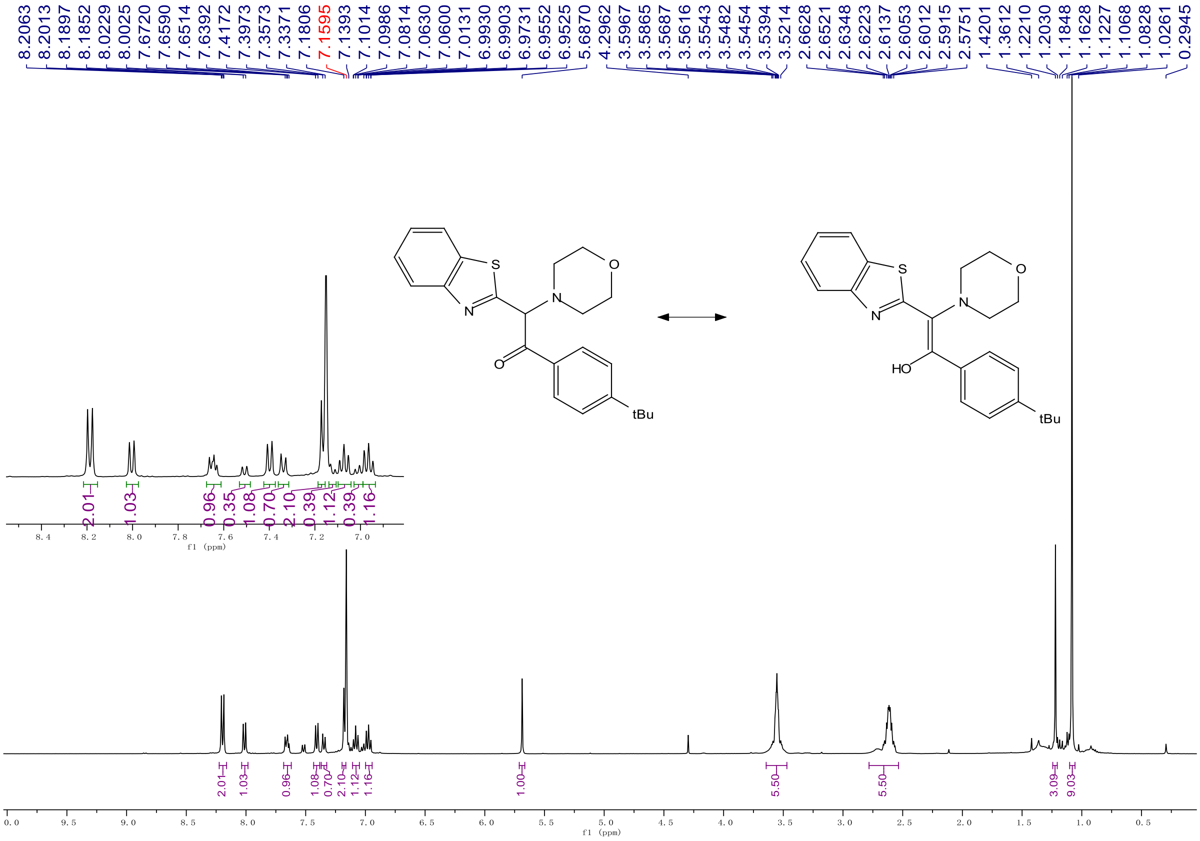

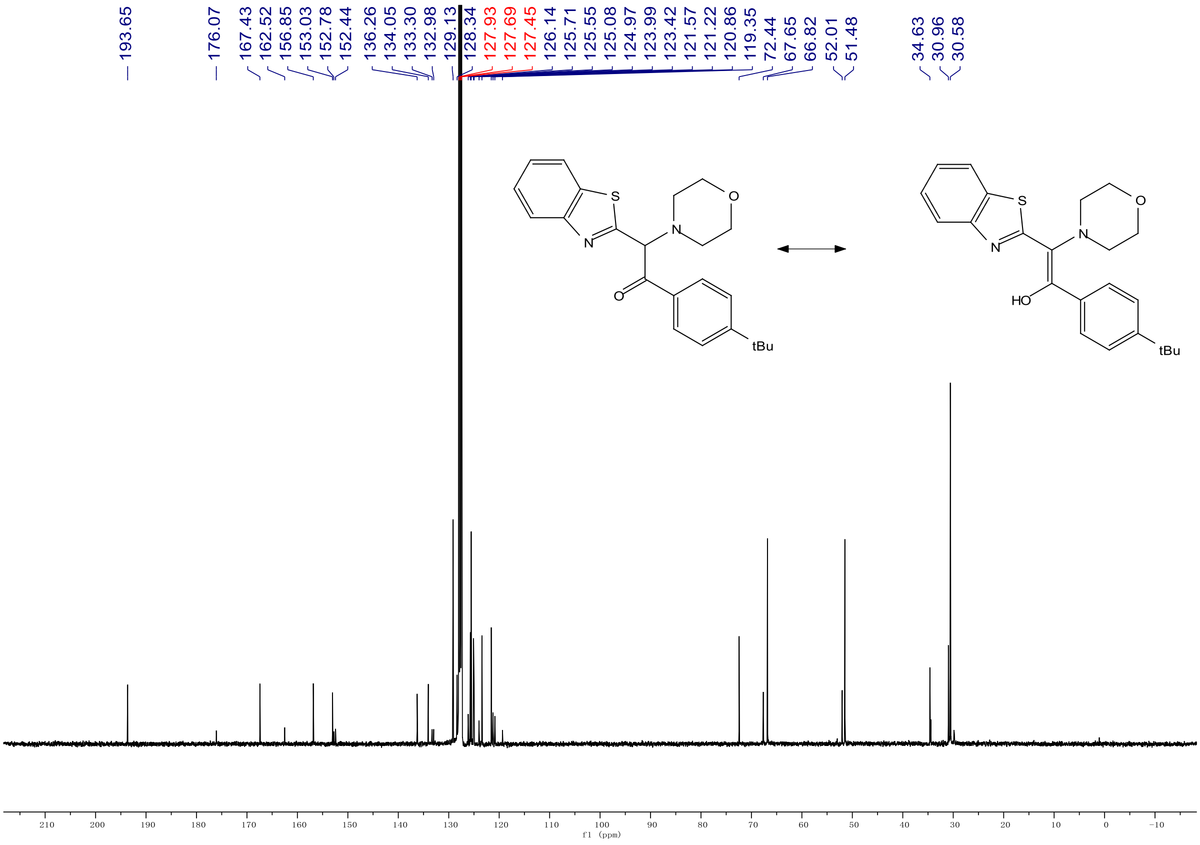

^{1}H and ^{13}C{^{1}H} NMR spectra of compound **3ma** in $CDCl_3$

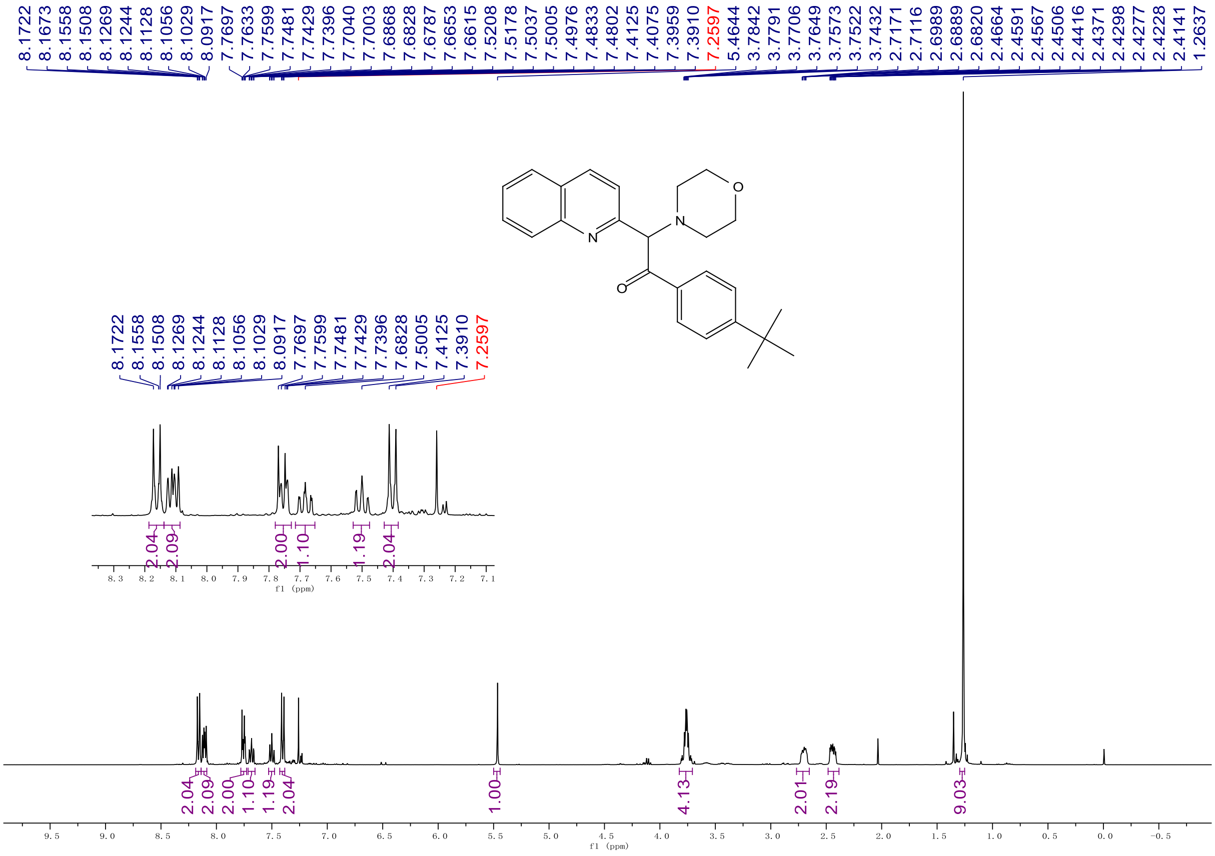

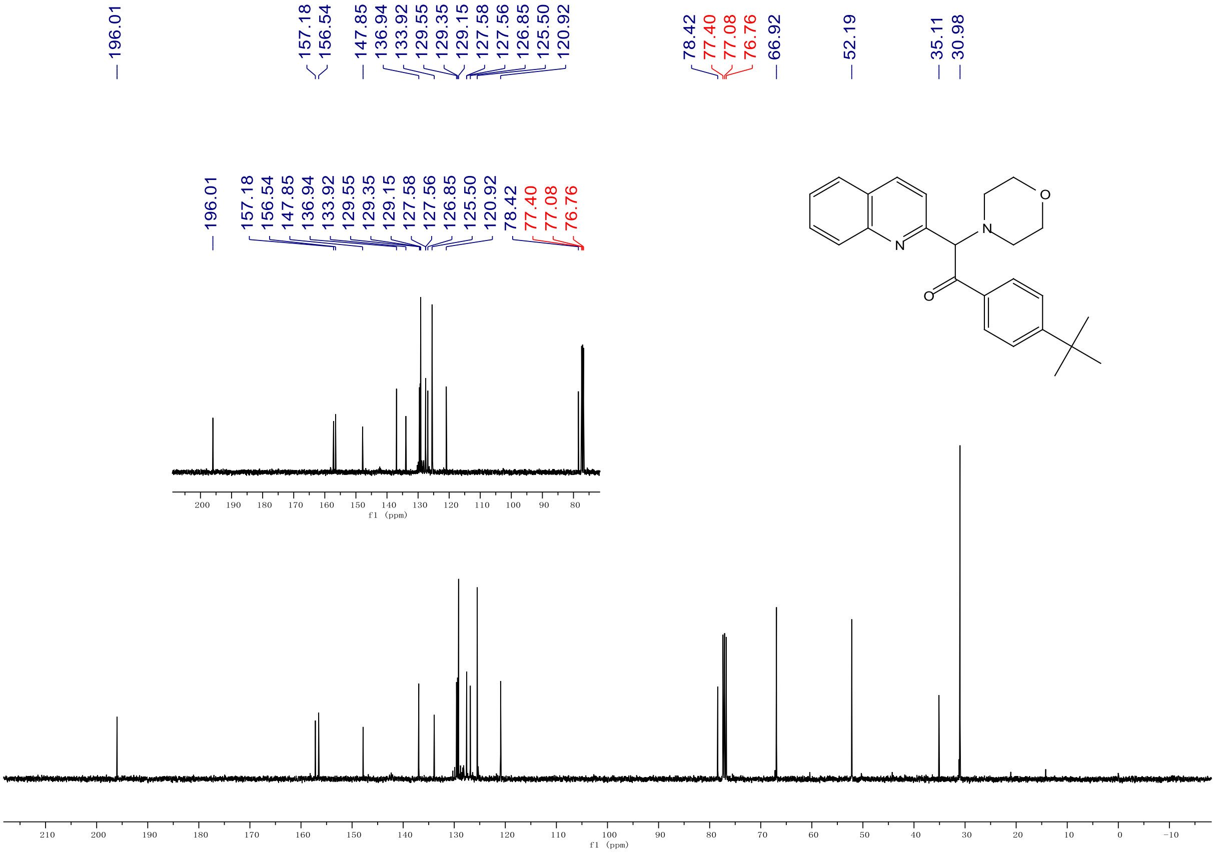

^{1}H and ^{13}C{^{1}H} NMR spectra of compound **3na** in $CDCl_3$

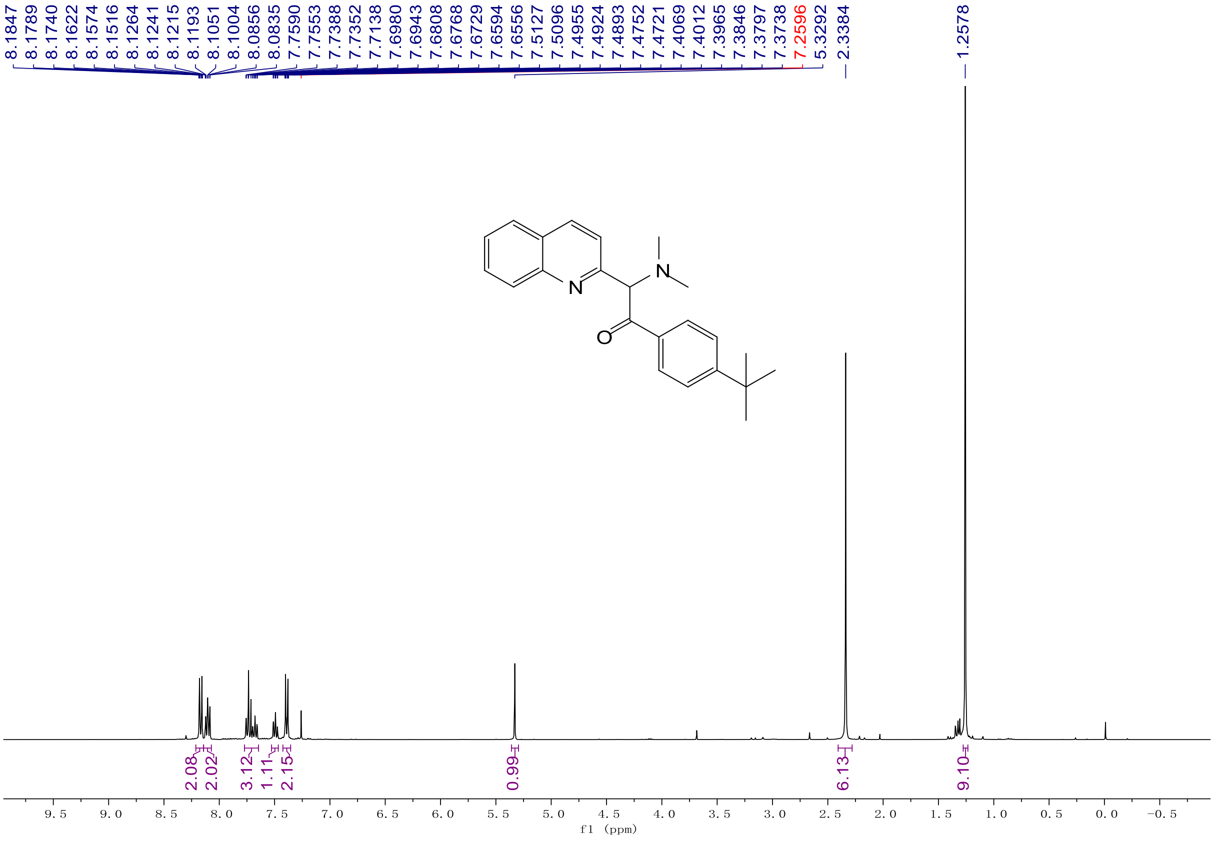

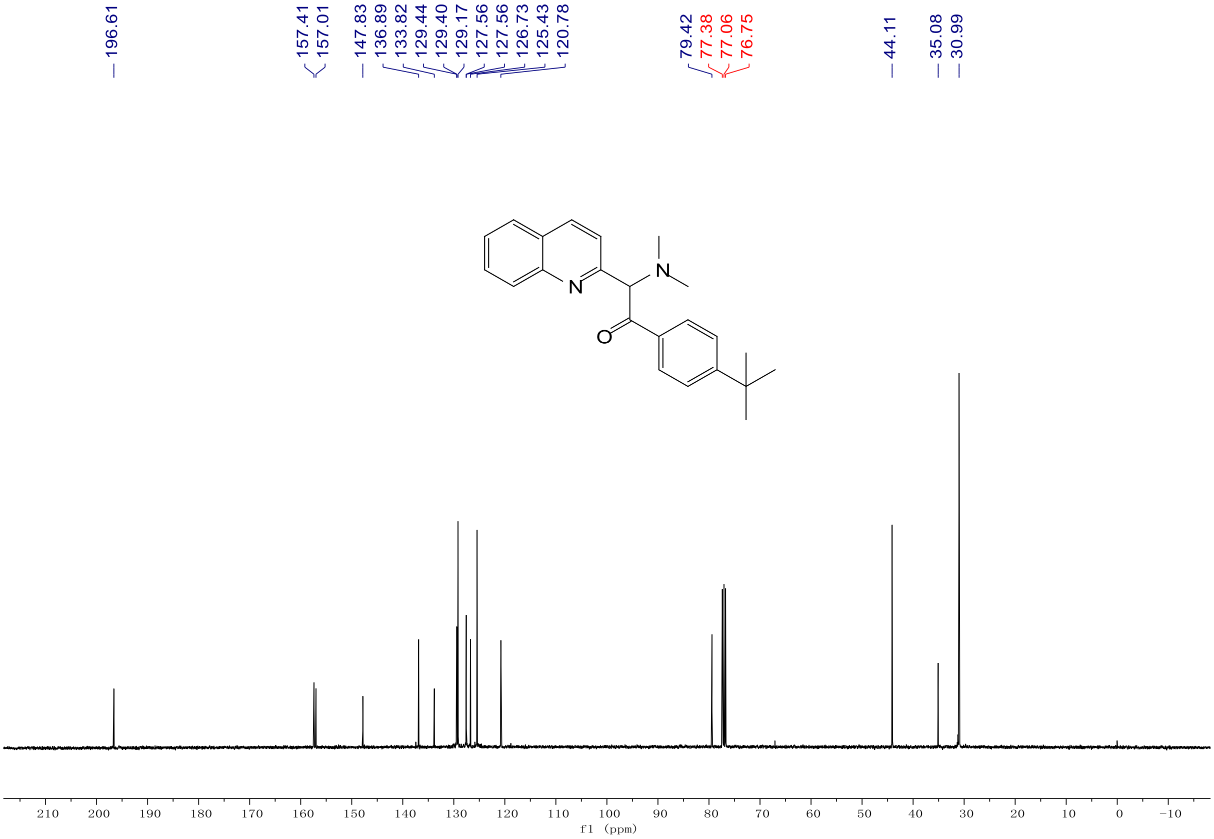

^{1}H and ^{13}C{^{1}H} NMR spectra of compound **3oa** in $CDCl_3$

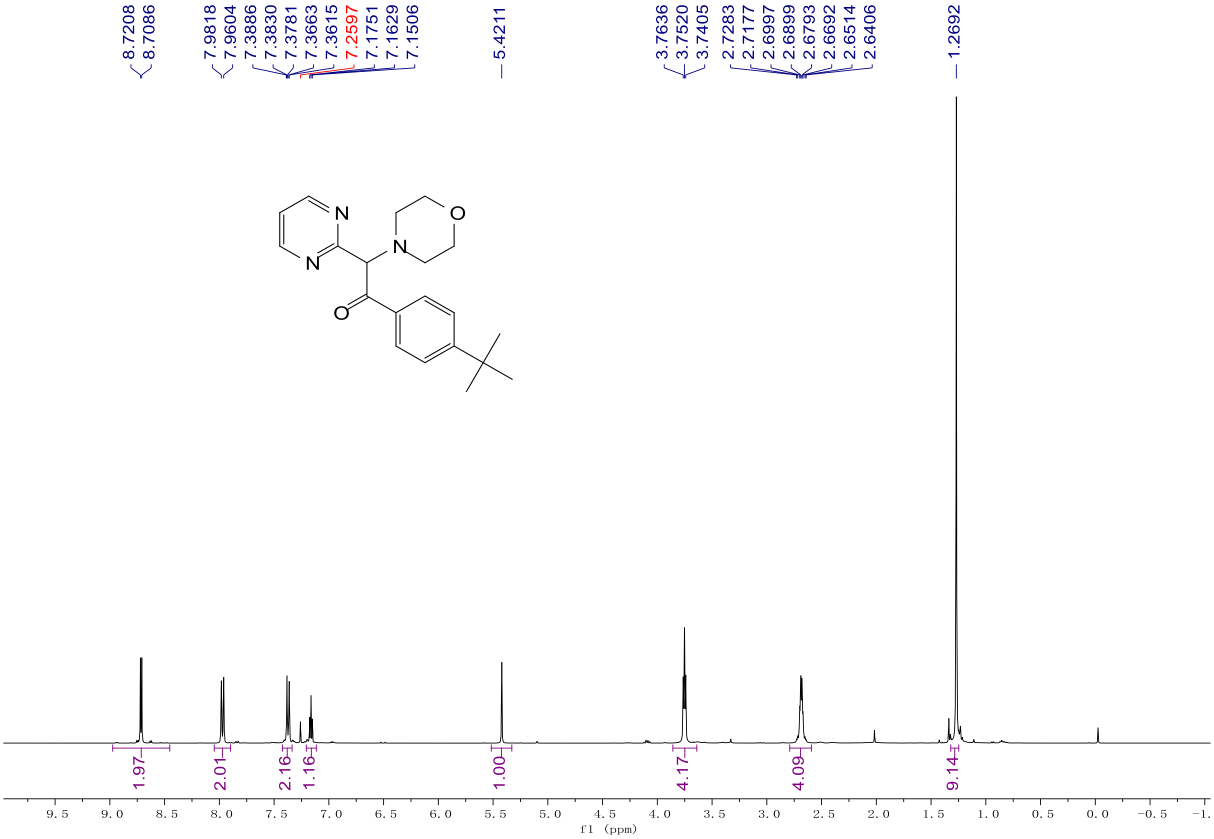

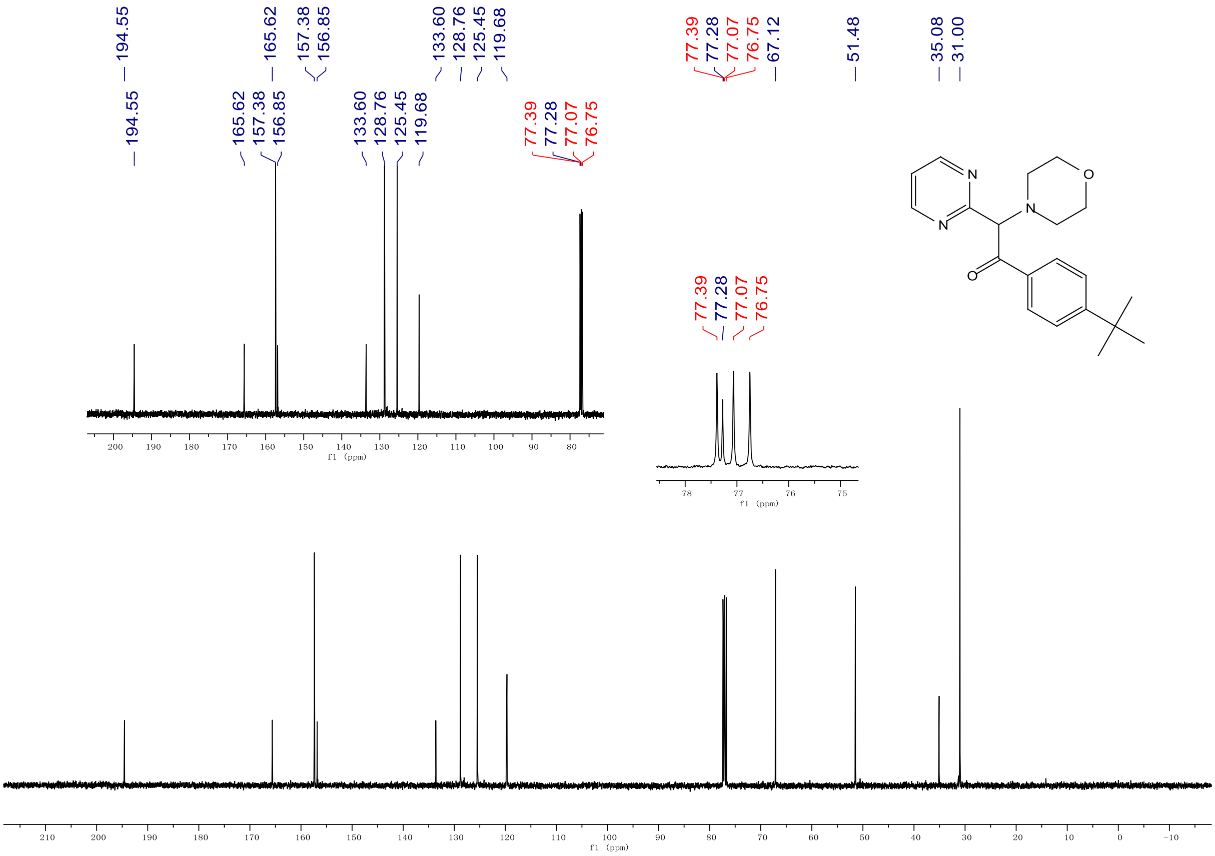

^{1}H and ^{13}C{^{1}H} NMR spectra of compound **3ab** in $CDCl_3$

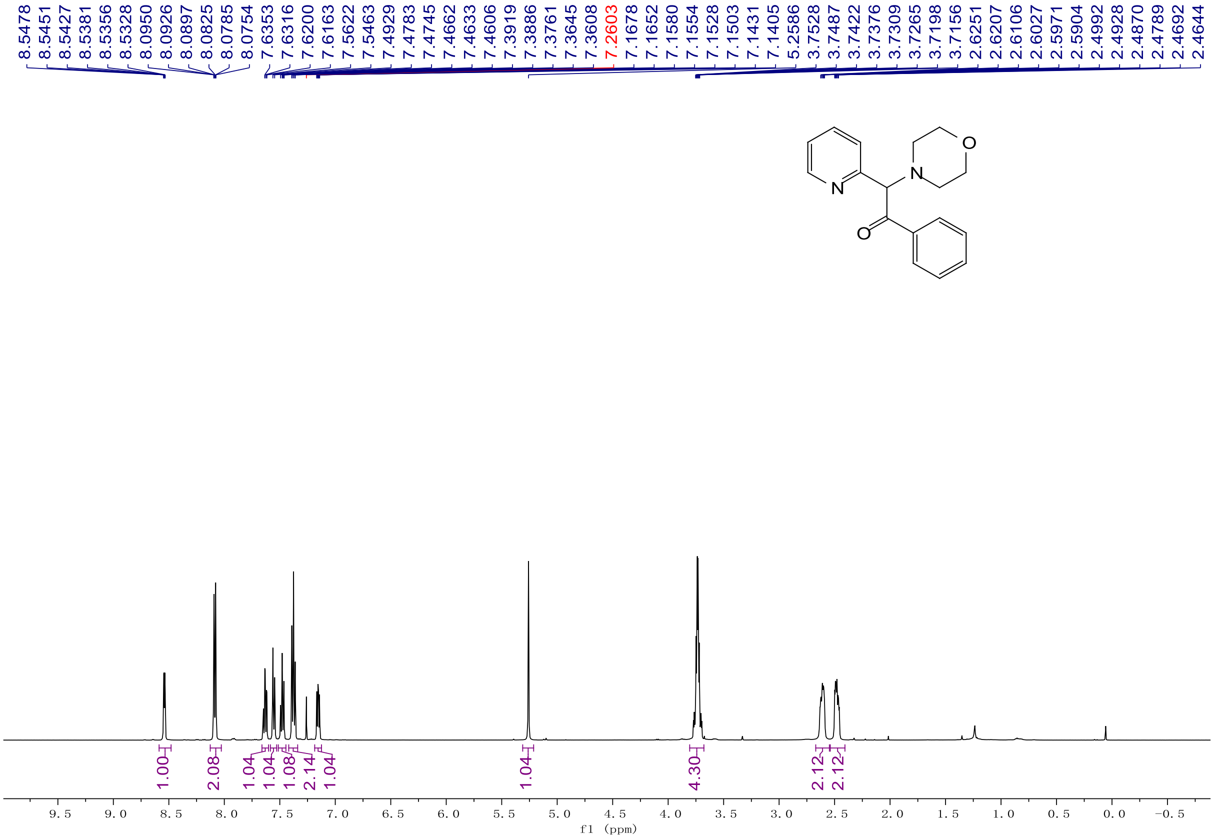

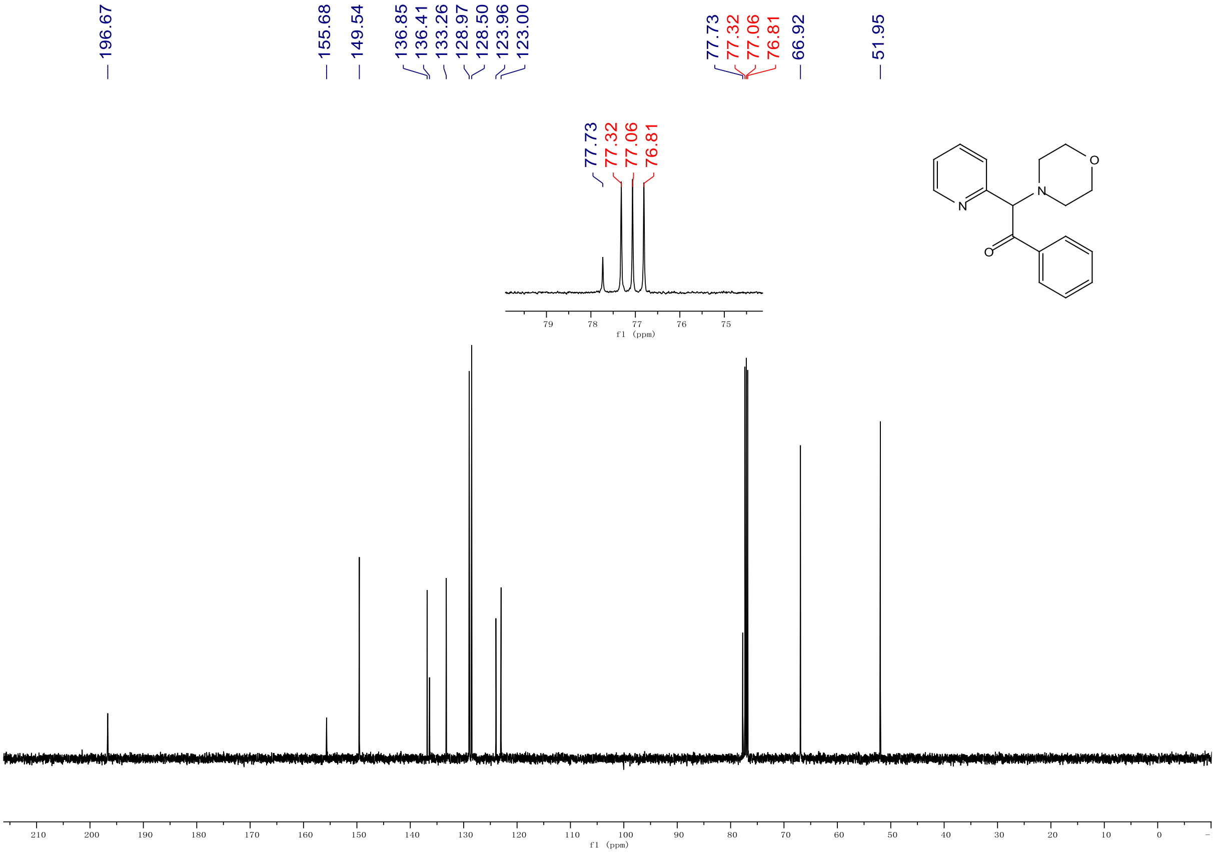

^{1}H and ^{13}C{^{1}H} NMR spectra of compound **3ac** in $CDCl_3$

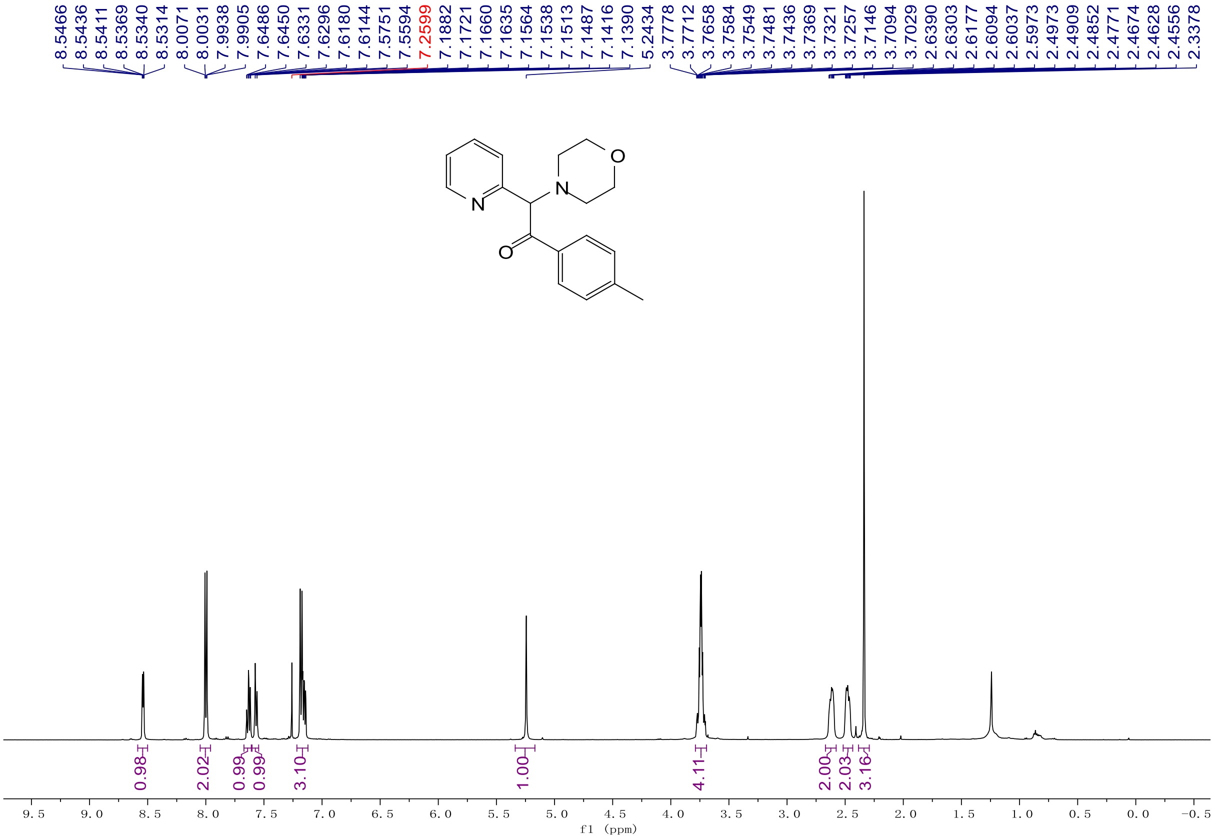

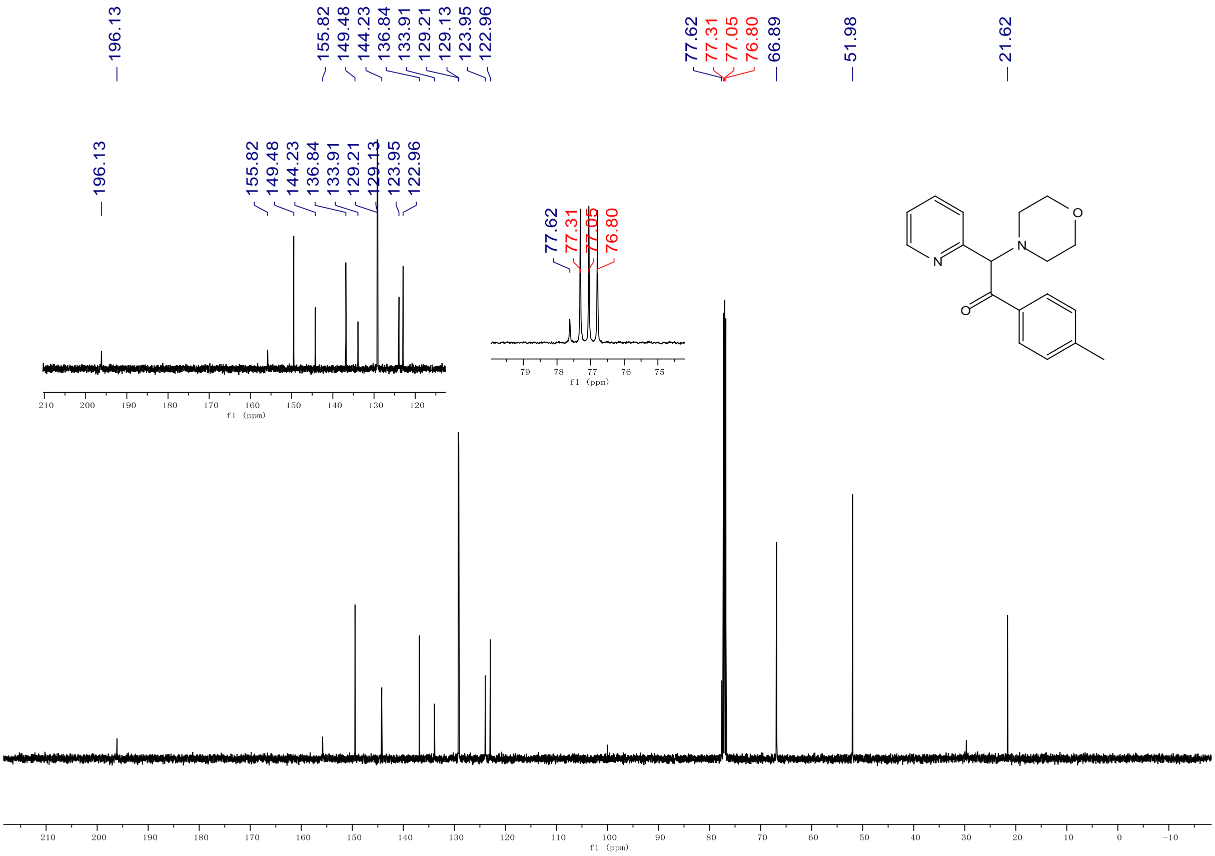

^{1}H and $^{13}C\{^{1}H\}$ NMR spectra of compound **3ad** in $CDCl_3$

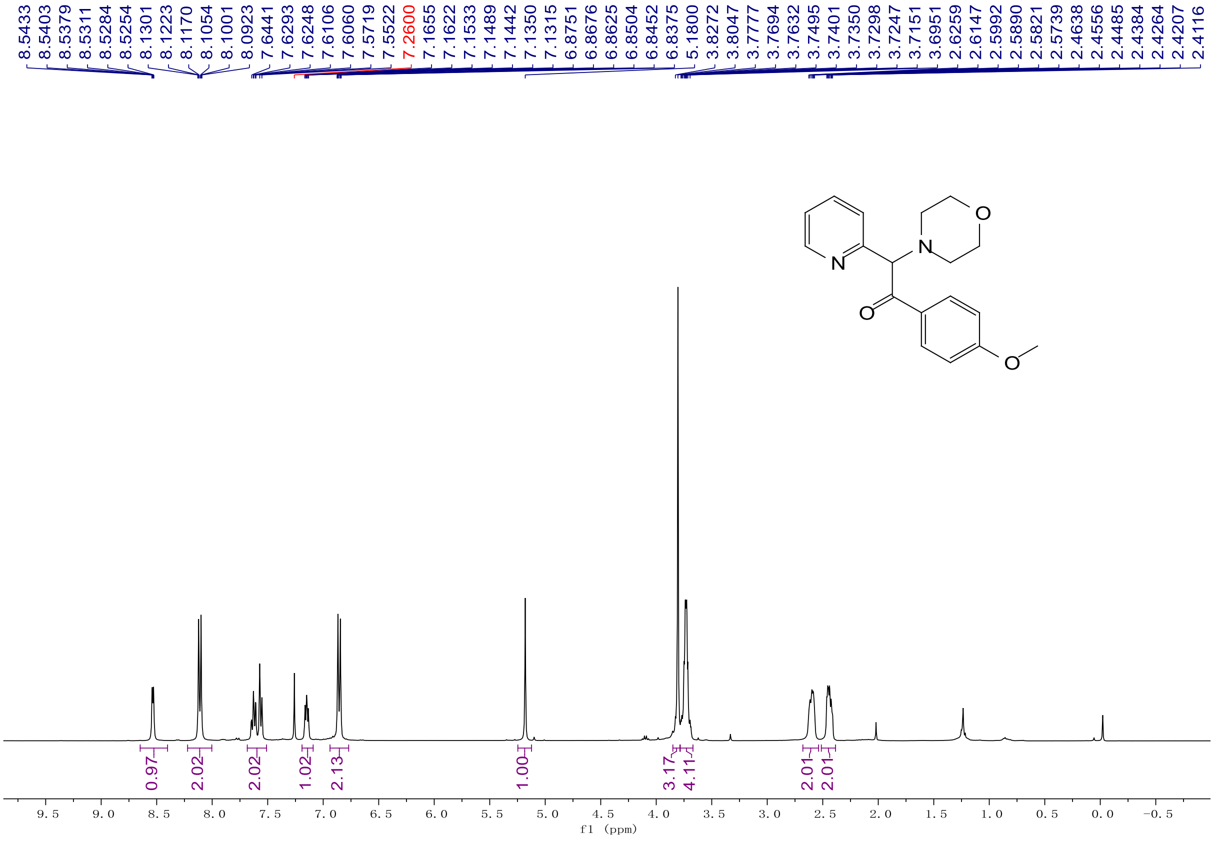

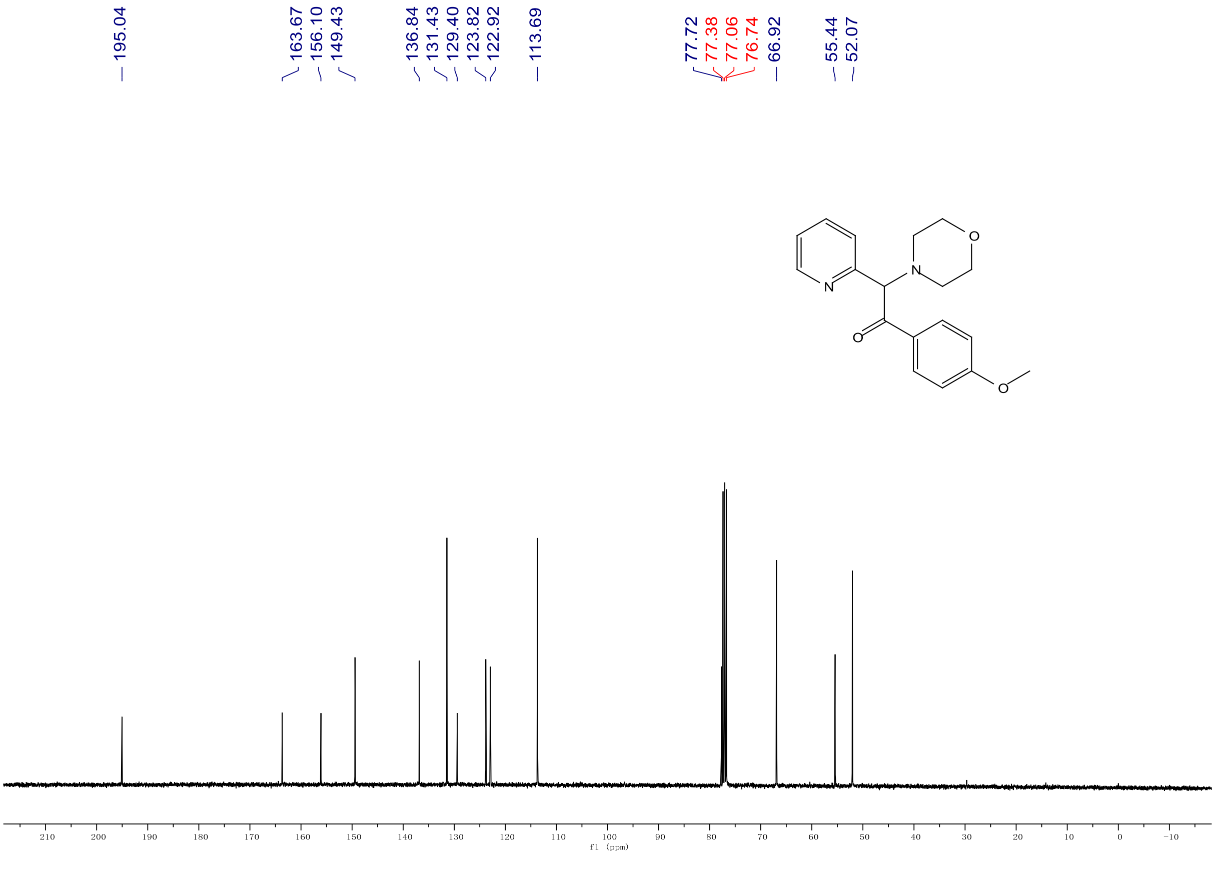

^{1}H and $^{13}C\{^1H\}$ NMR spectra of compound **3ae** in $CDCl_3$

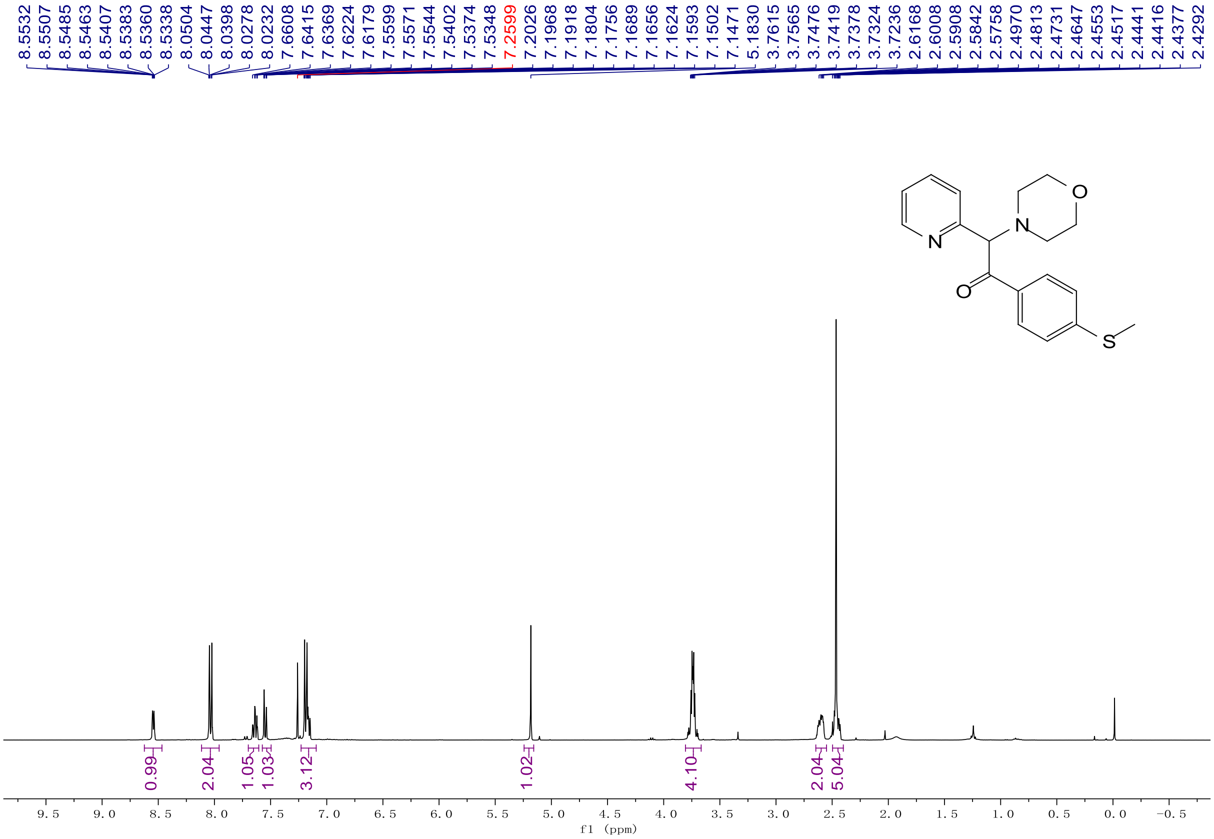

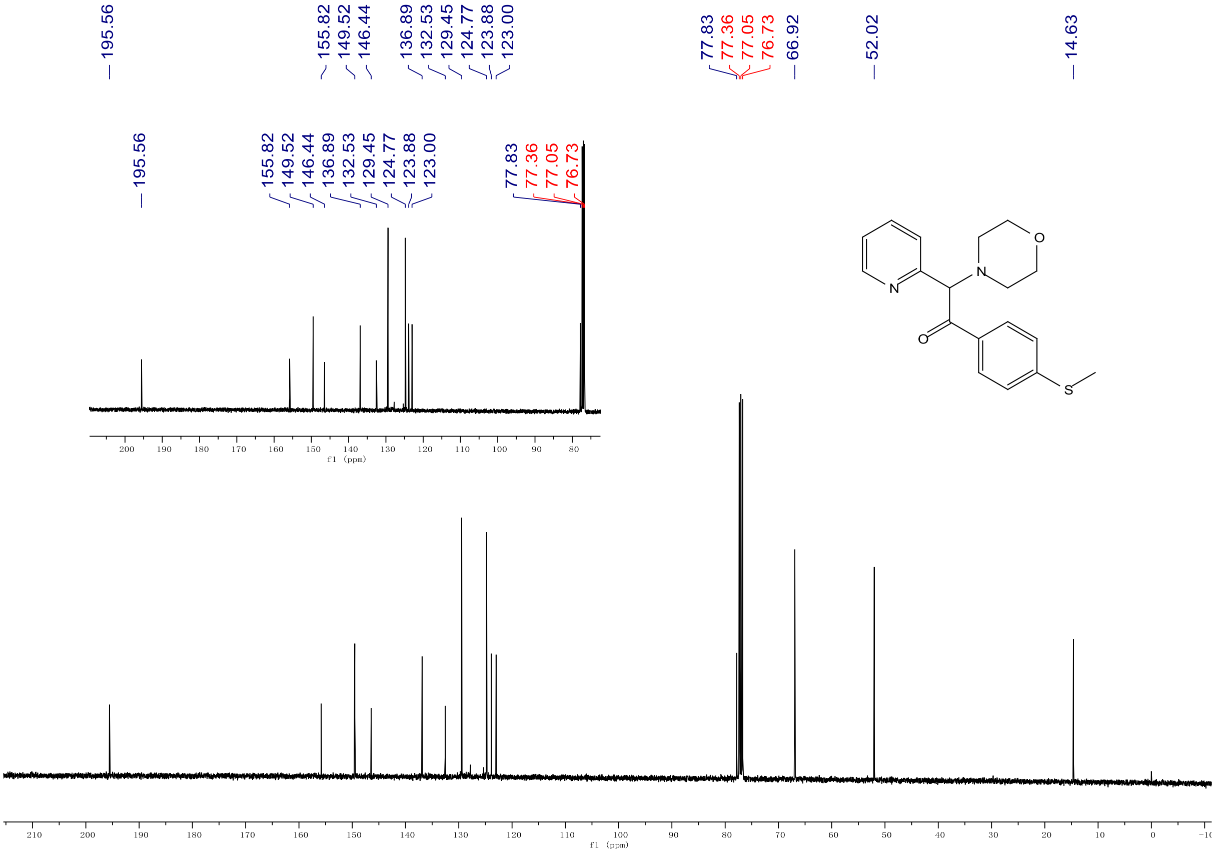

^{1}H and ^{13}C{^{1}H} NMR spectra of compound **3af** in $CDCl_3$

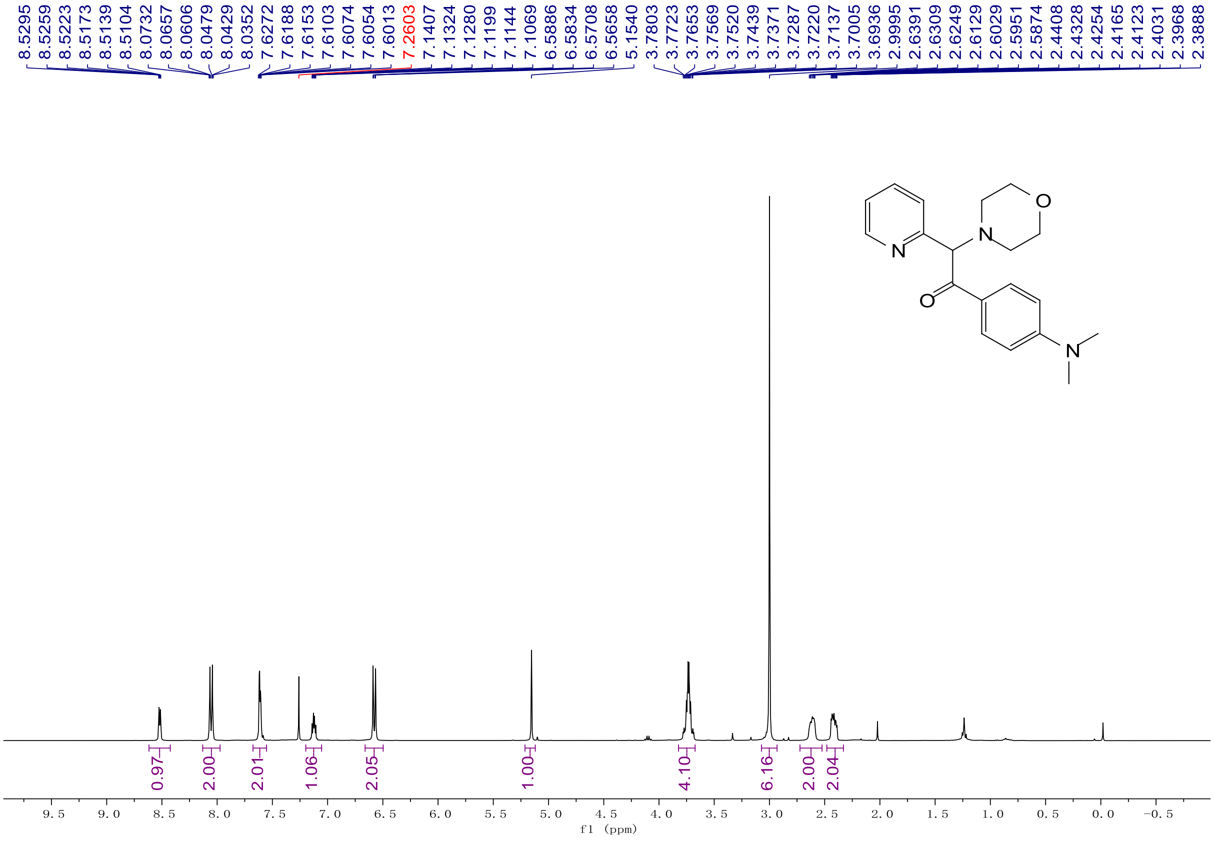

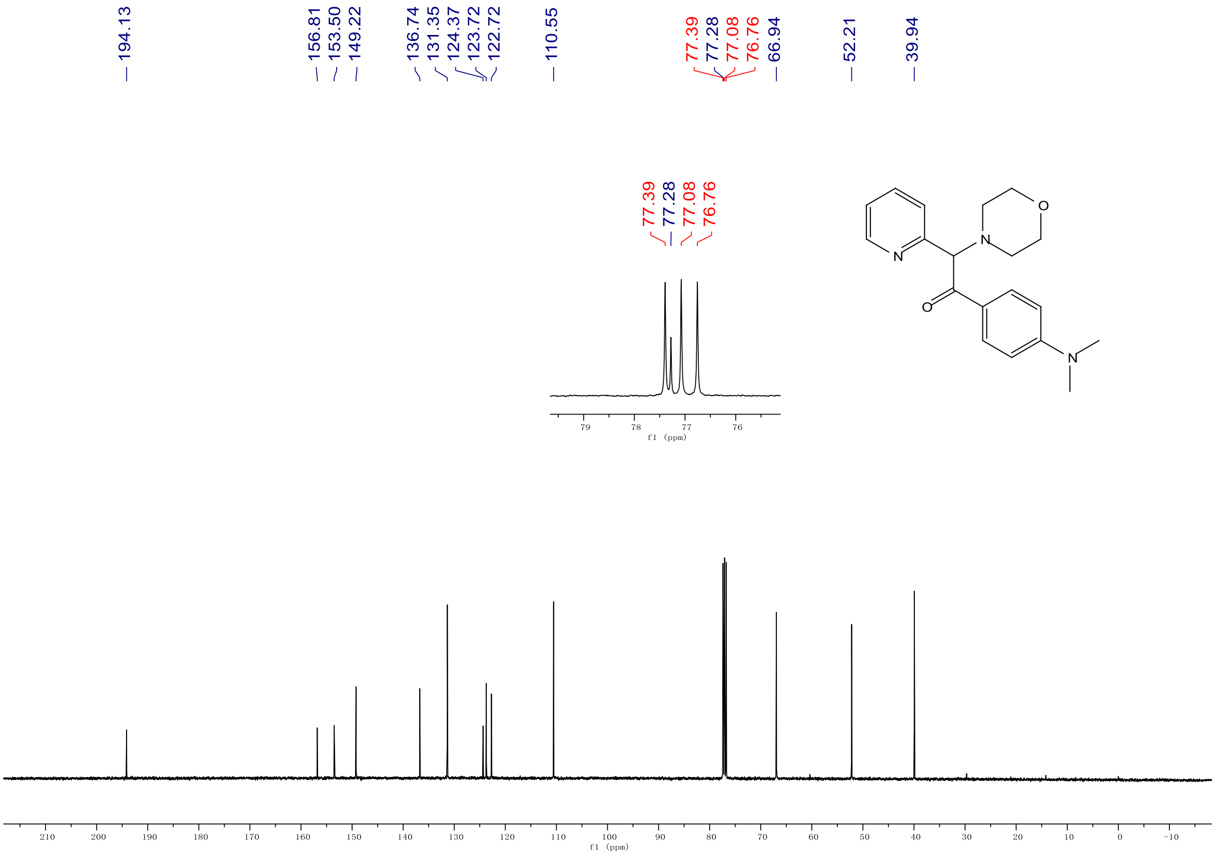

^{1}H, $^{13}C\{^{1}H\}$ and ^{19}F NMR spectra of compound **3ag** in $CDCl_3$

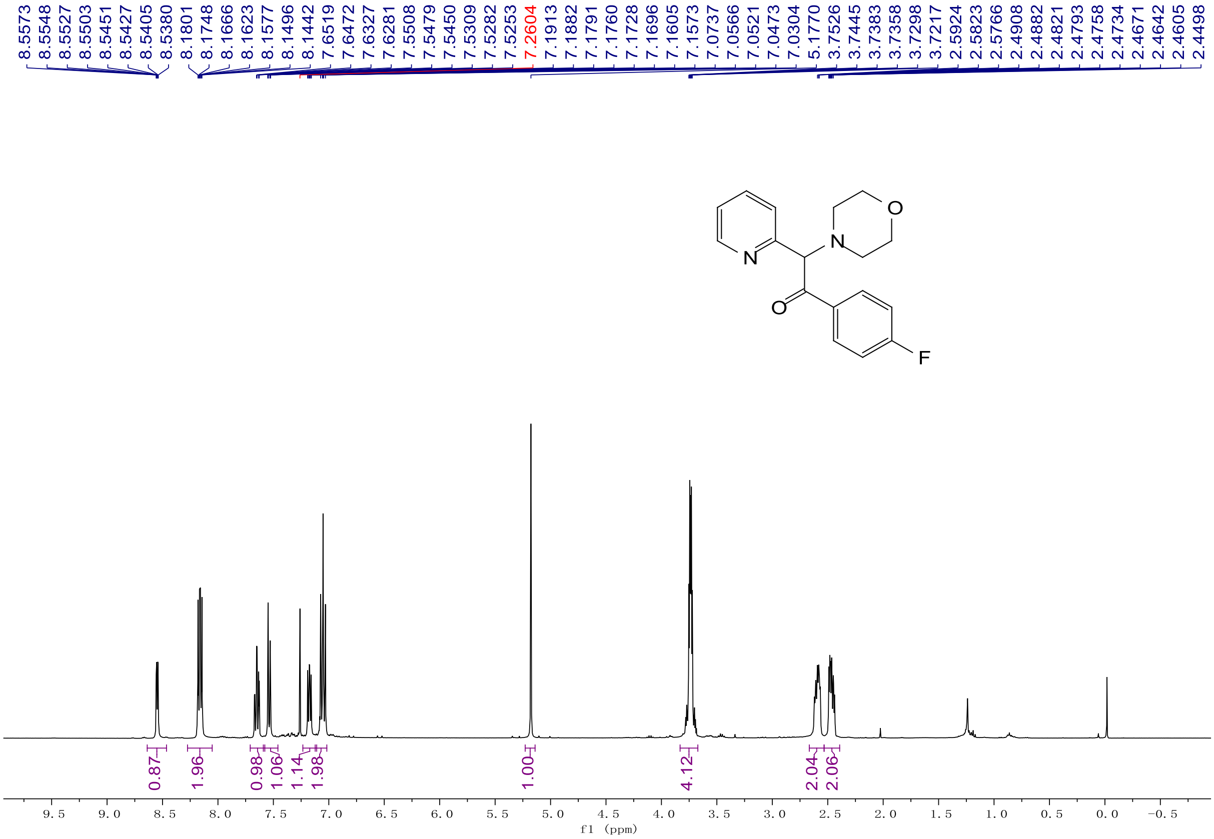

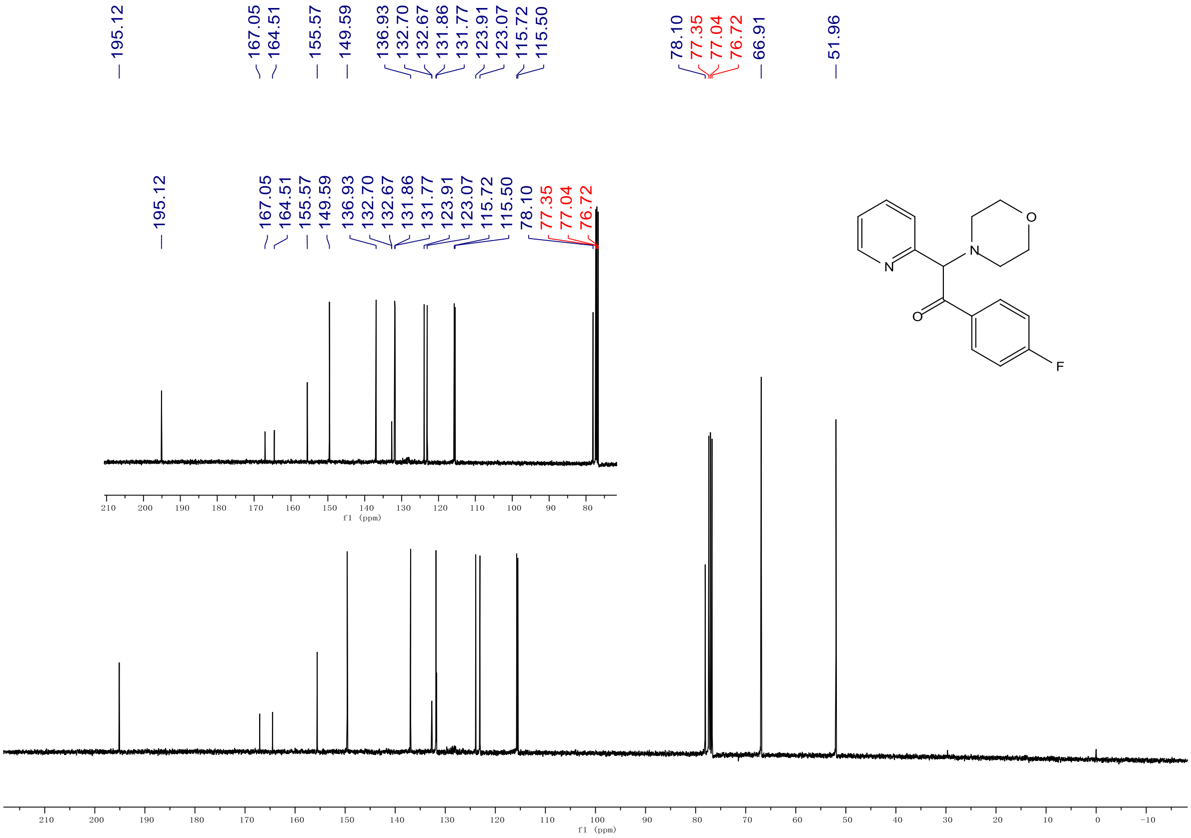

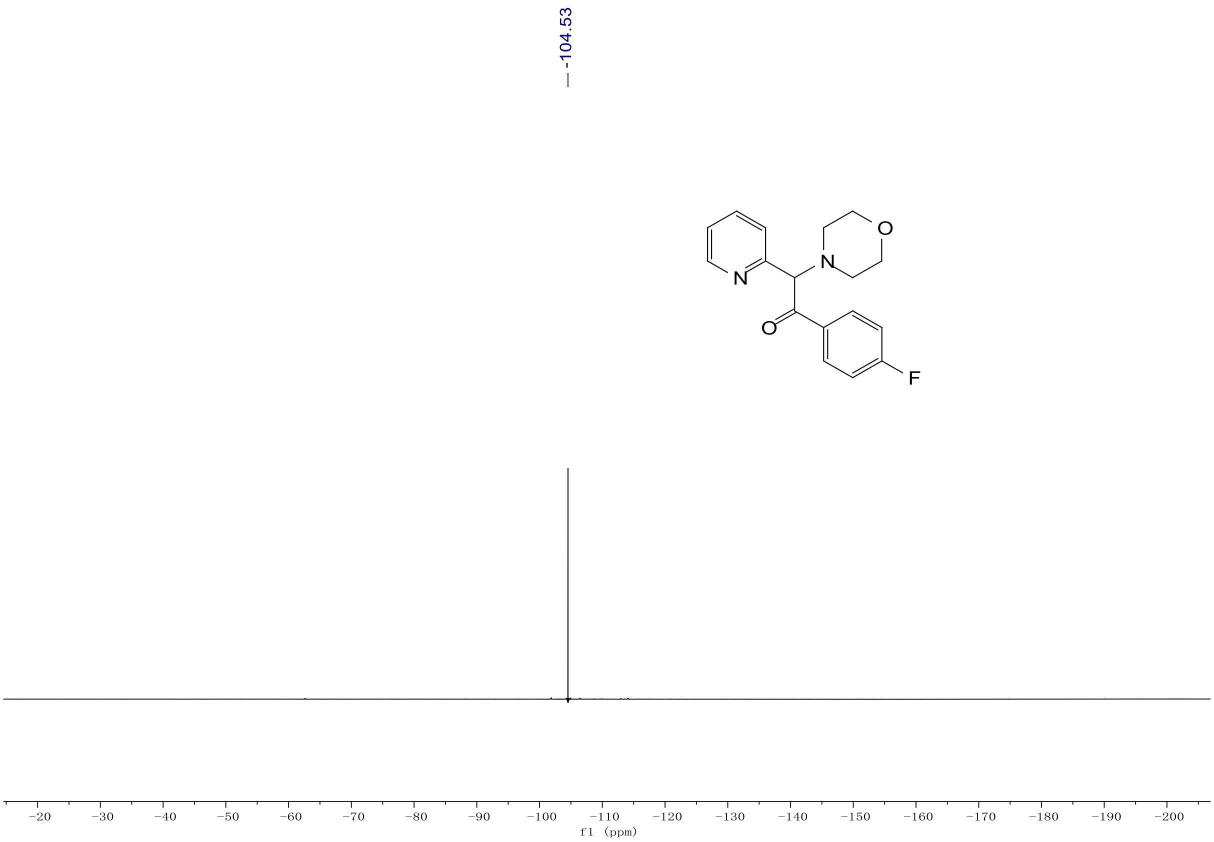
-104.53
N
N
O
O
F
-20 -30 -40 -50 -60 -70 -80 -90 -100 -110 -120 -130 -140 -150 -160 -170 -180 -190 -200
f1 (ppm)

^{1}H and $^{13}C\{^{1}H\}$ NMR spectra of compound **3ah** in $CDCl_3$

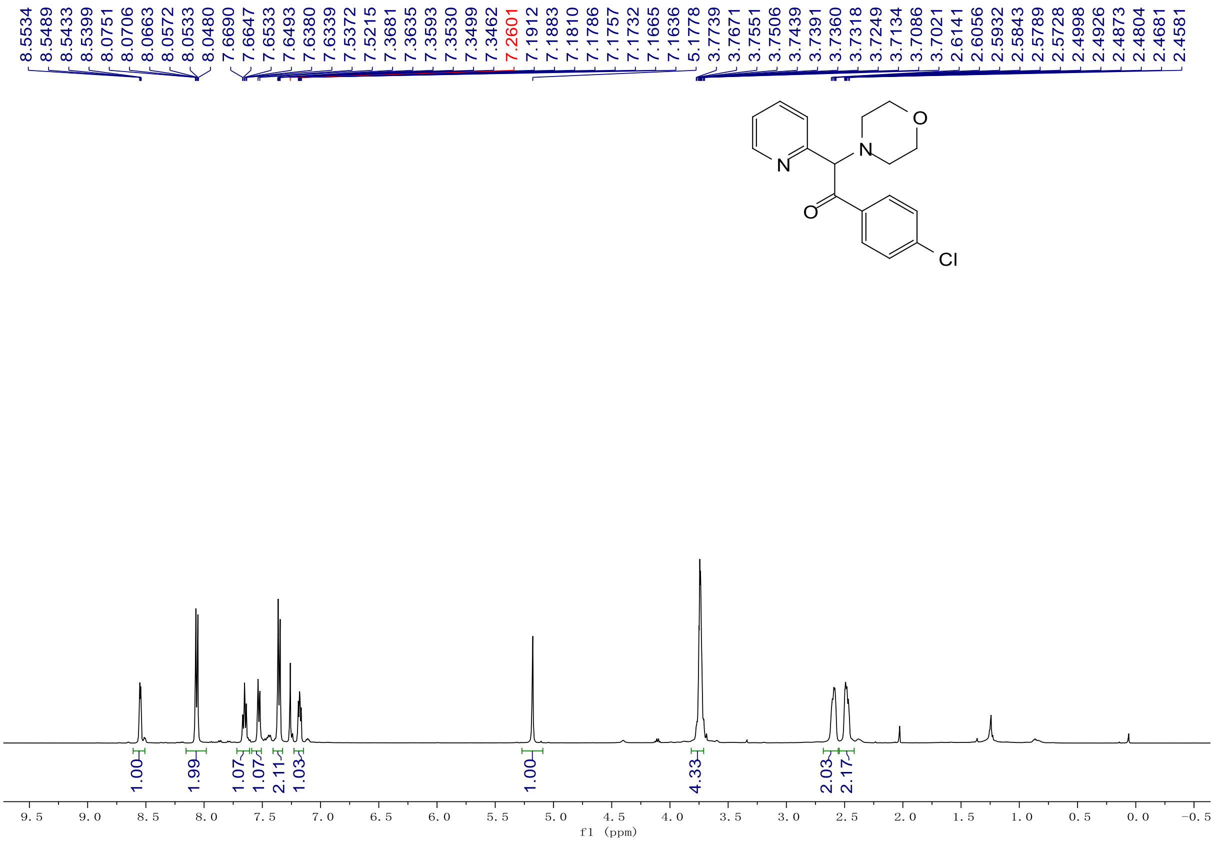

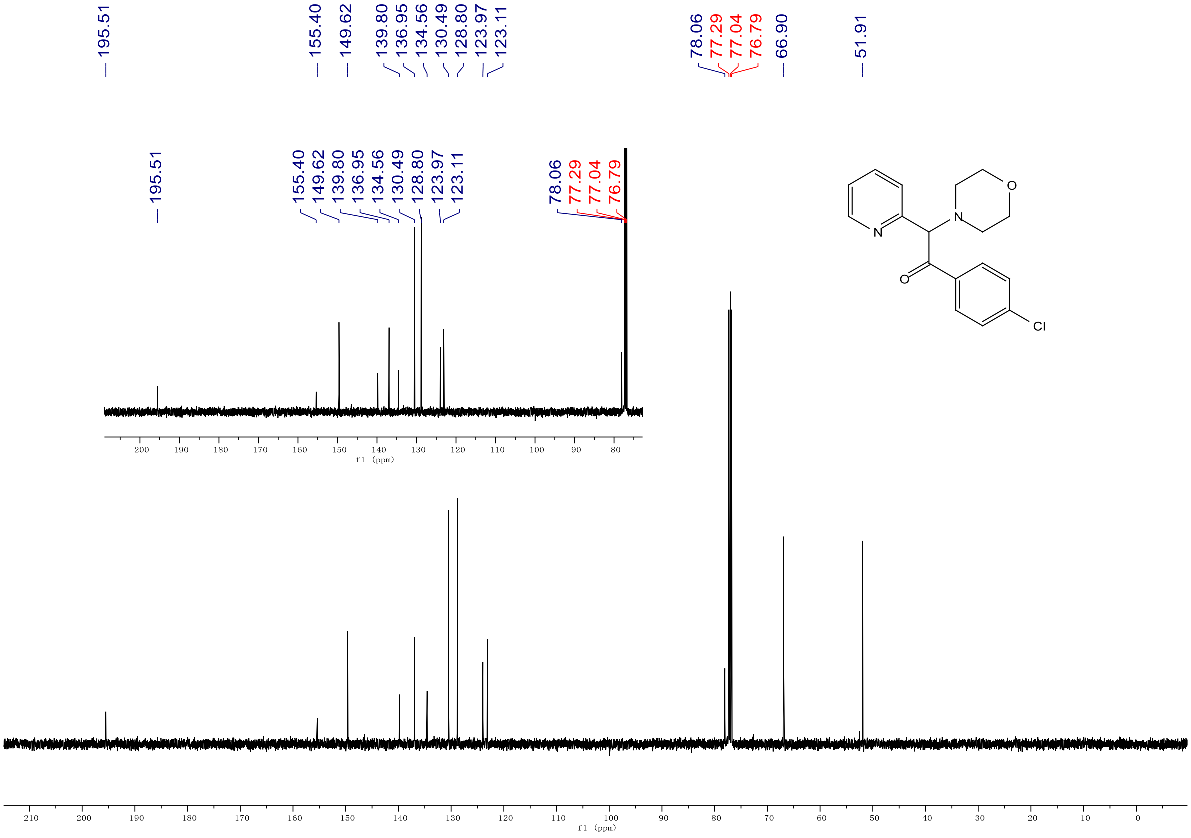

^{1}H, $^{13}C\{^{1}H\}$ and ^{19}F NMR spectra of compound **3ai** in $CDCl_3$

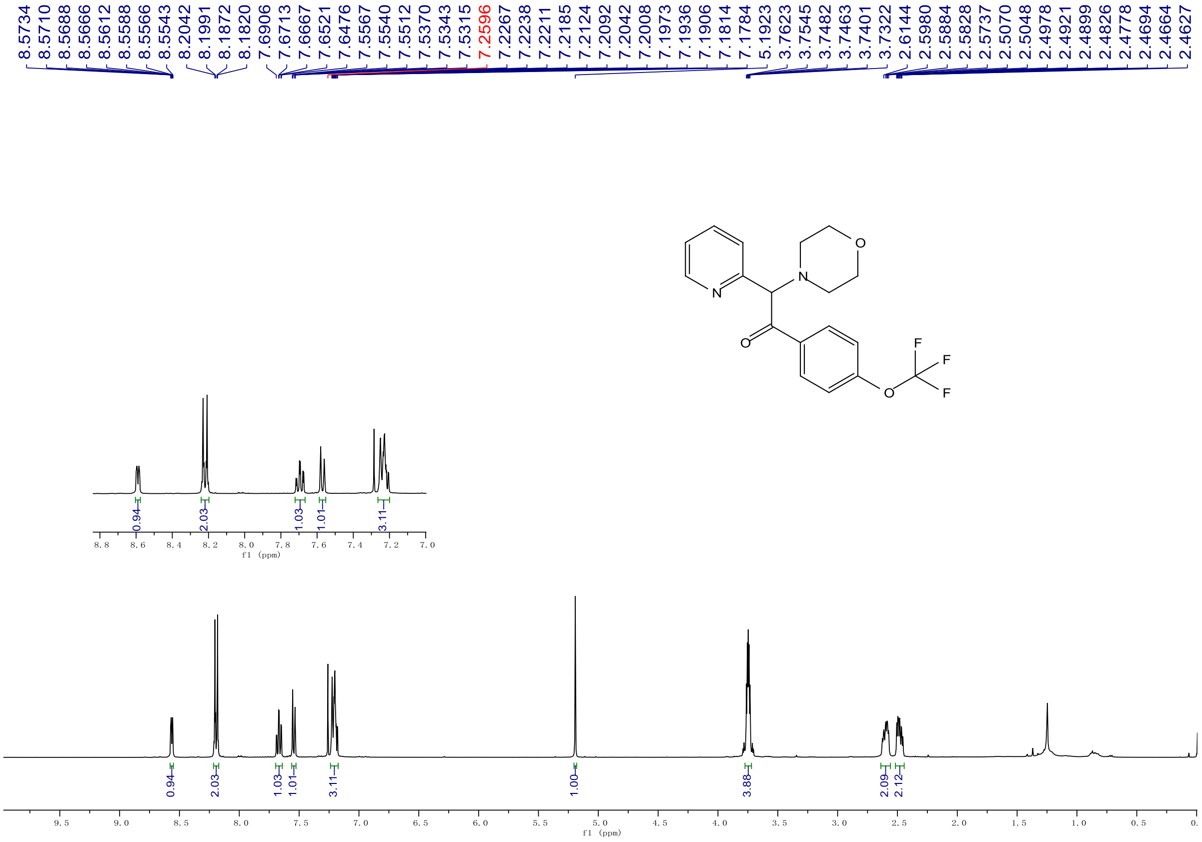

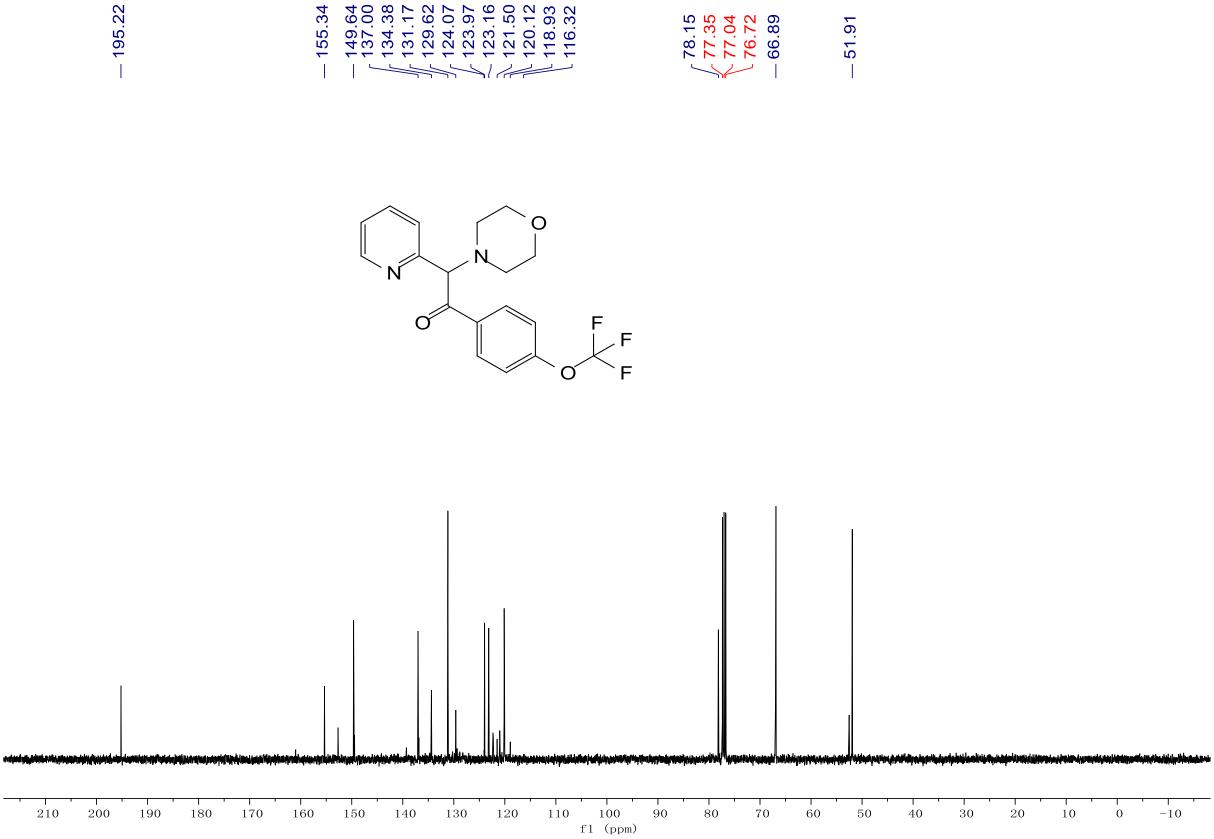

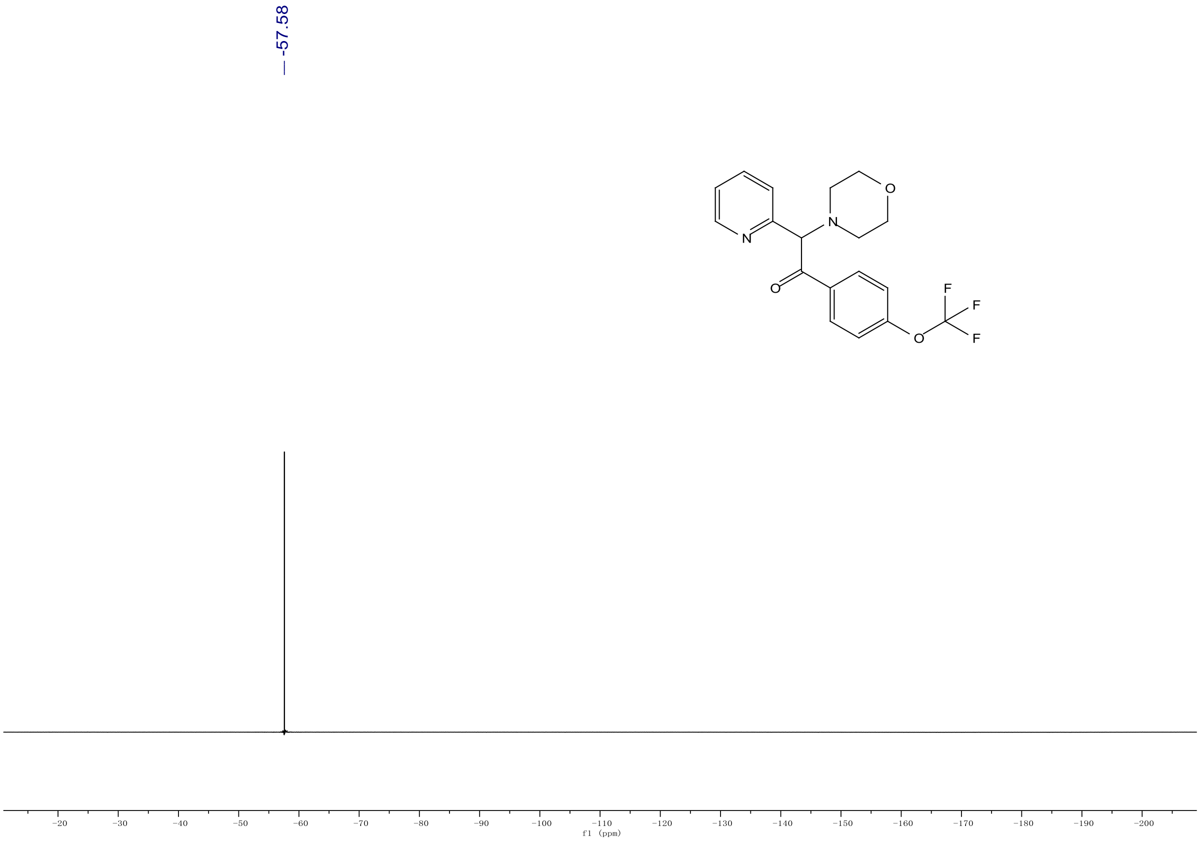
-57.58
N
N
O
O
O
F
F
F
-20
-30
-40
-50
-60
-70
-80
-90
-100
-110
-120
-130
-140
-150
-160
-170
-180
-190
-200
f1 (ppm)

^{1}H and ^{13}C{^{1}H} NMR spectra of compound **3aj** in $CDCl_3$

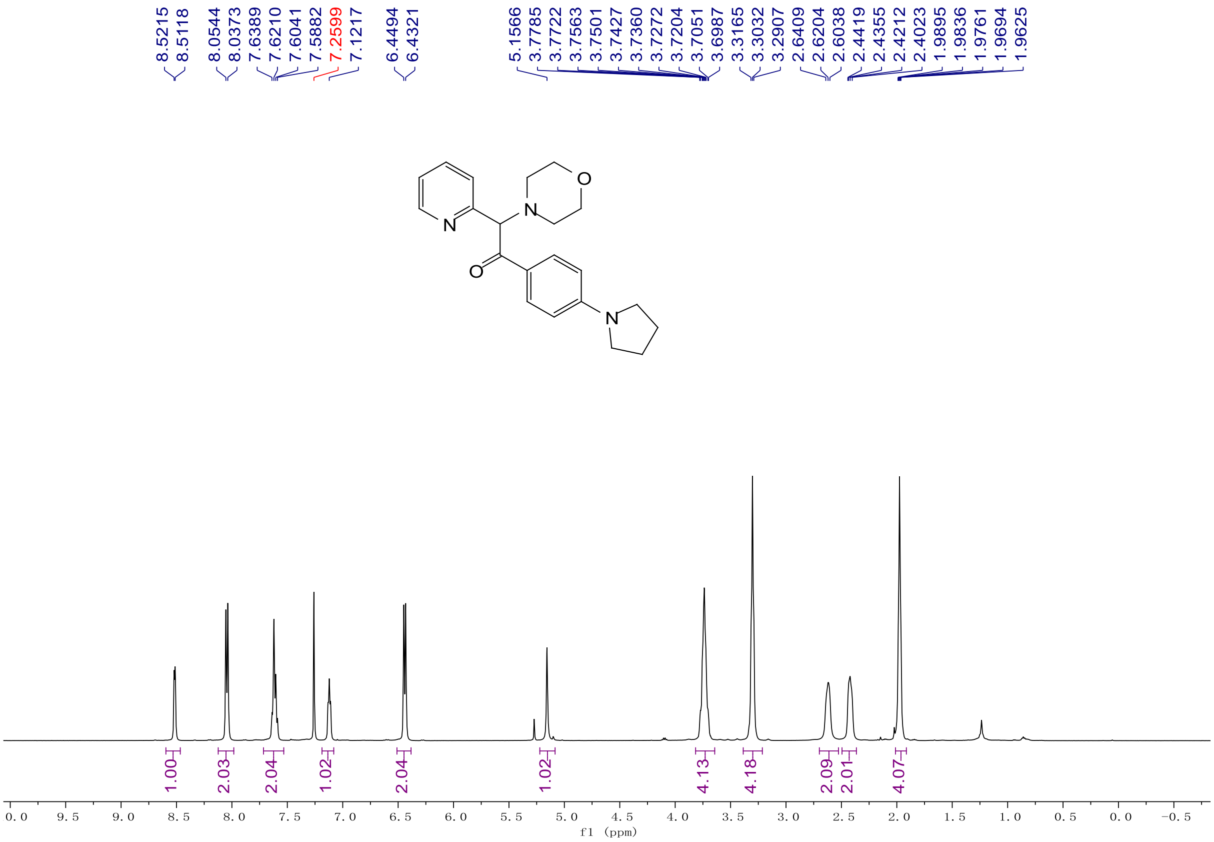

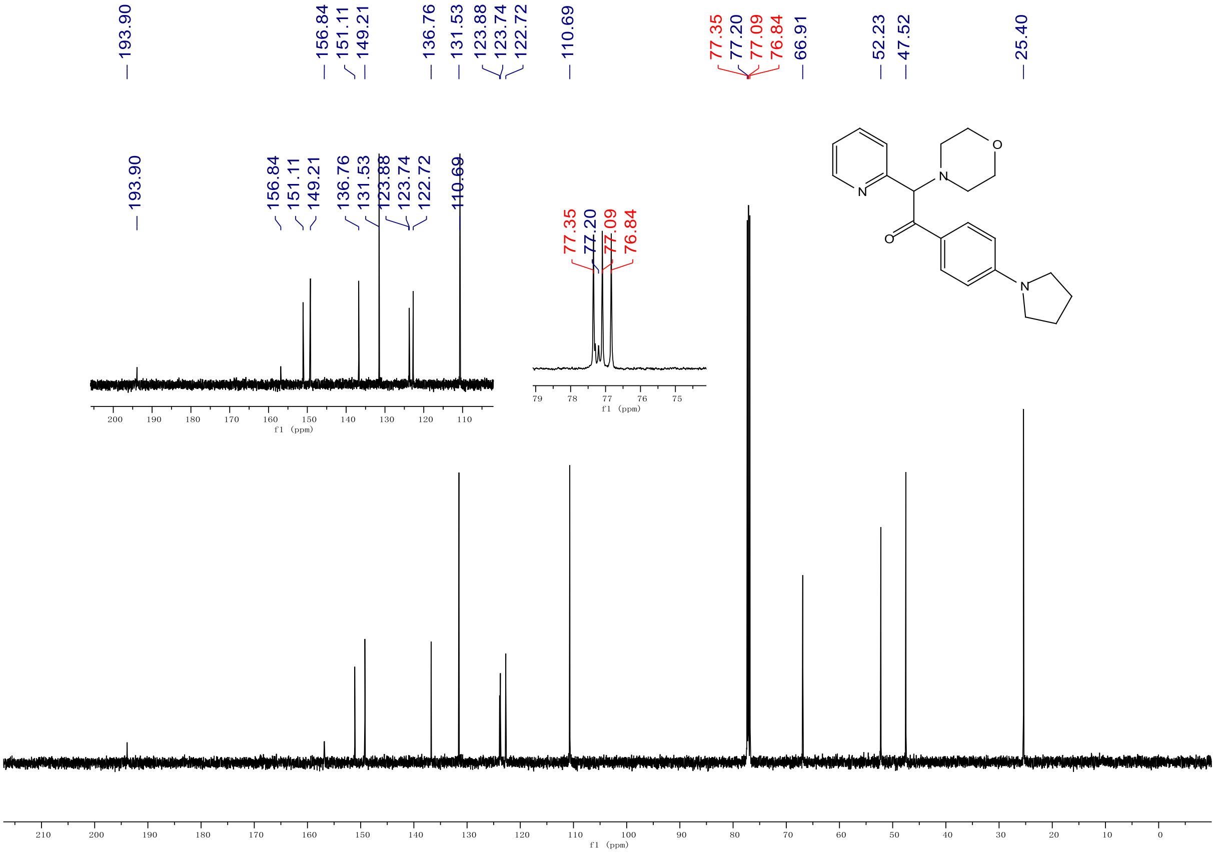

^{1}H and ^{13}C{^{1}H} NMR spectra of compound **3ak** in $CDCl_3$

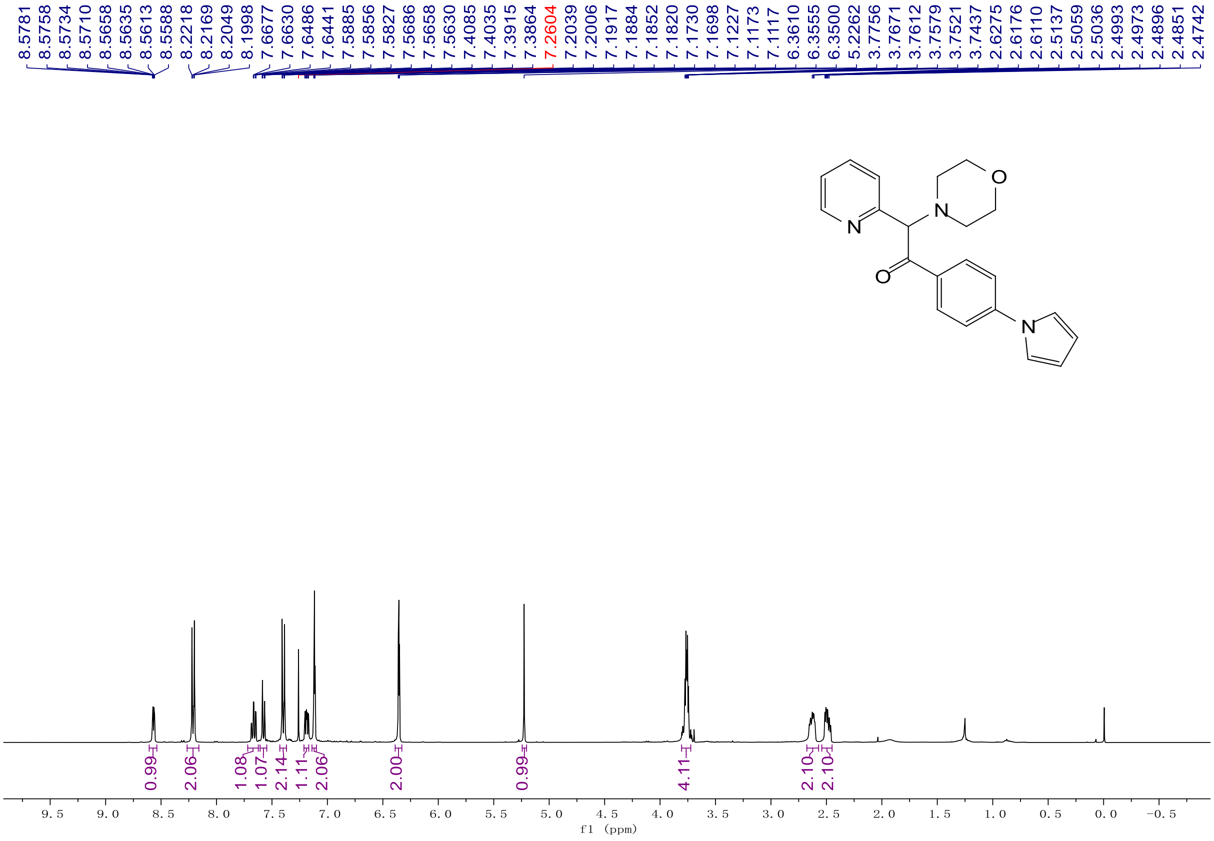

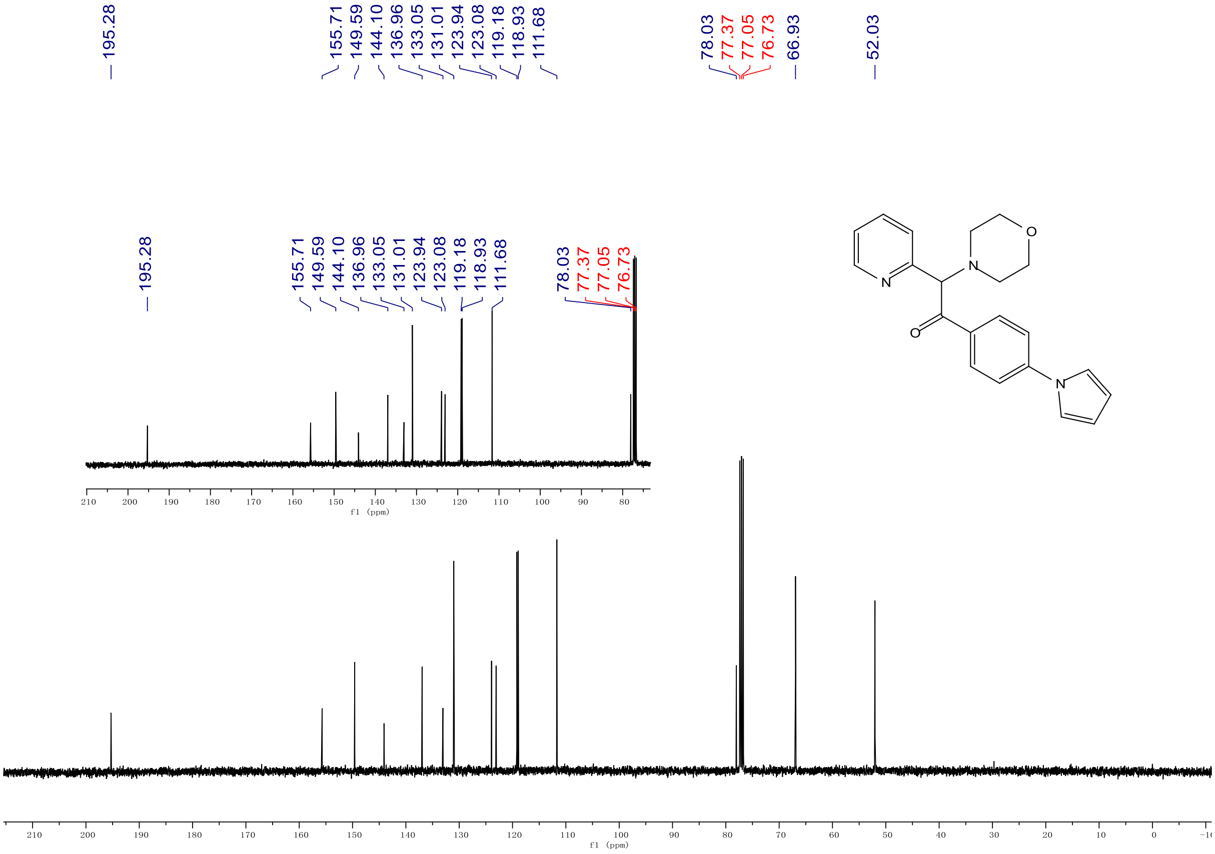

^{1}H and $^{13}C\{^{1}H\}$ NMR spectra of compound **3al** in $CDCl_3$

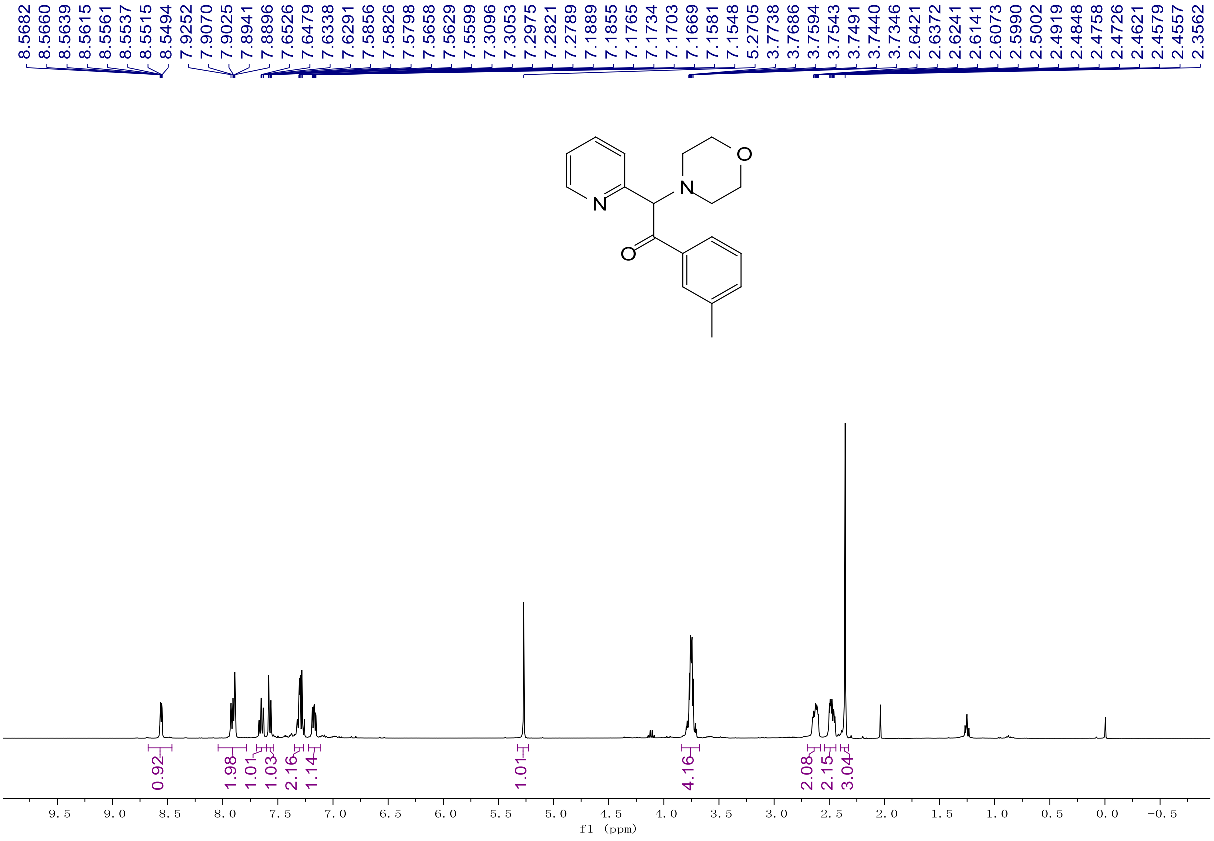

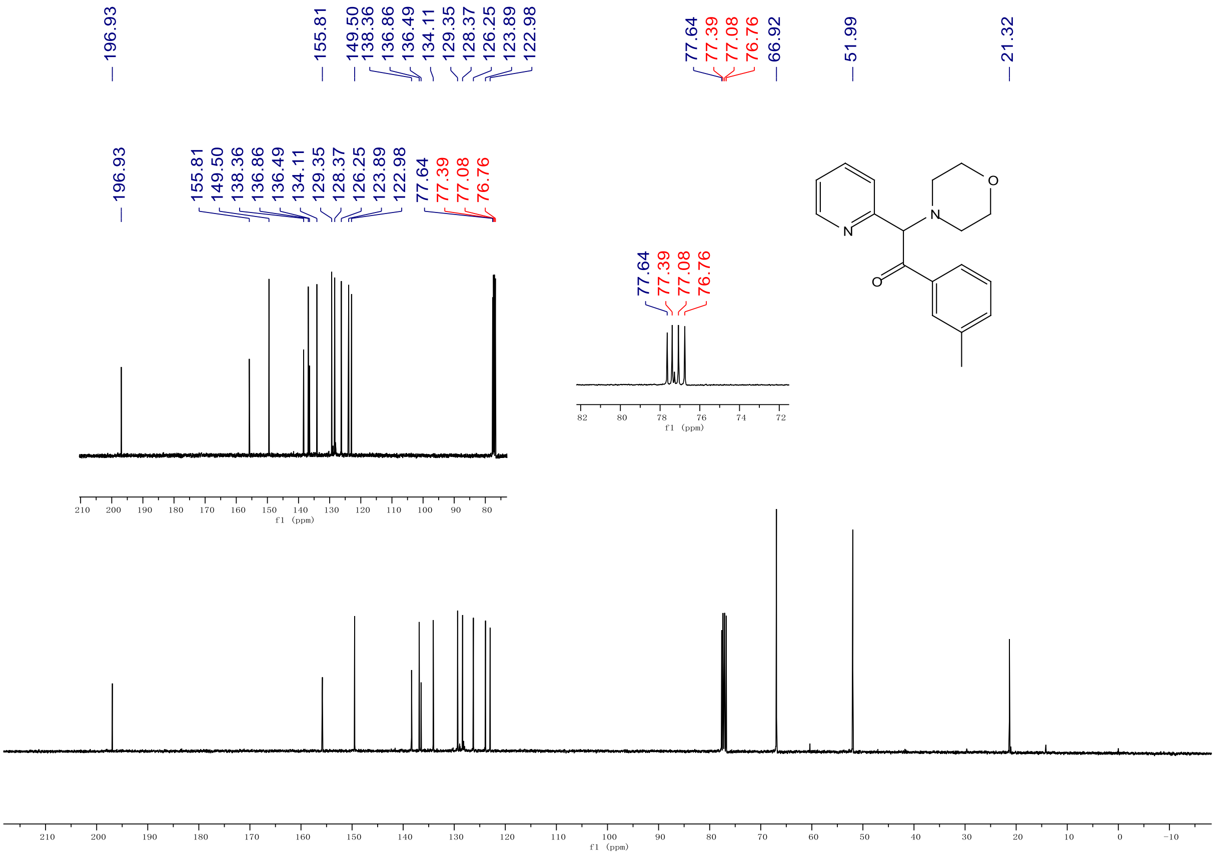

^{1}H and $^{13}C\{^{1}H\}$ NMR spectra of compound **3am** in $CDCl_3$

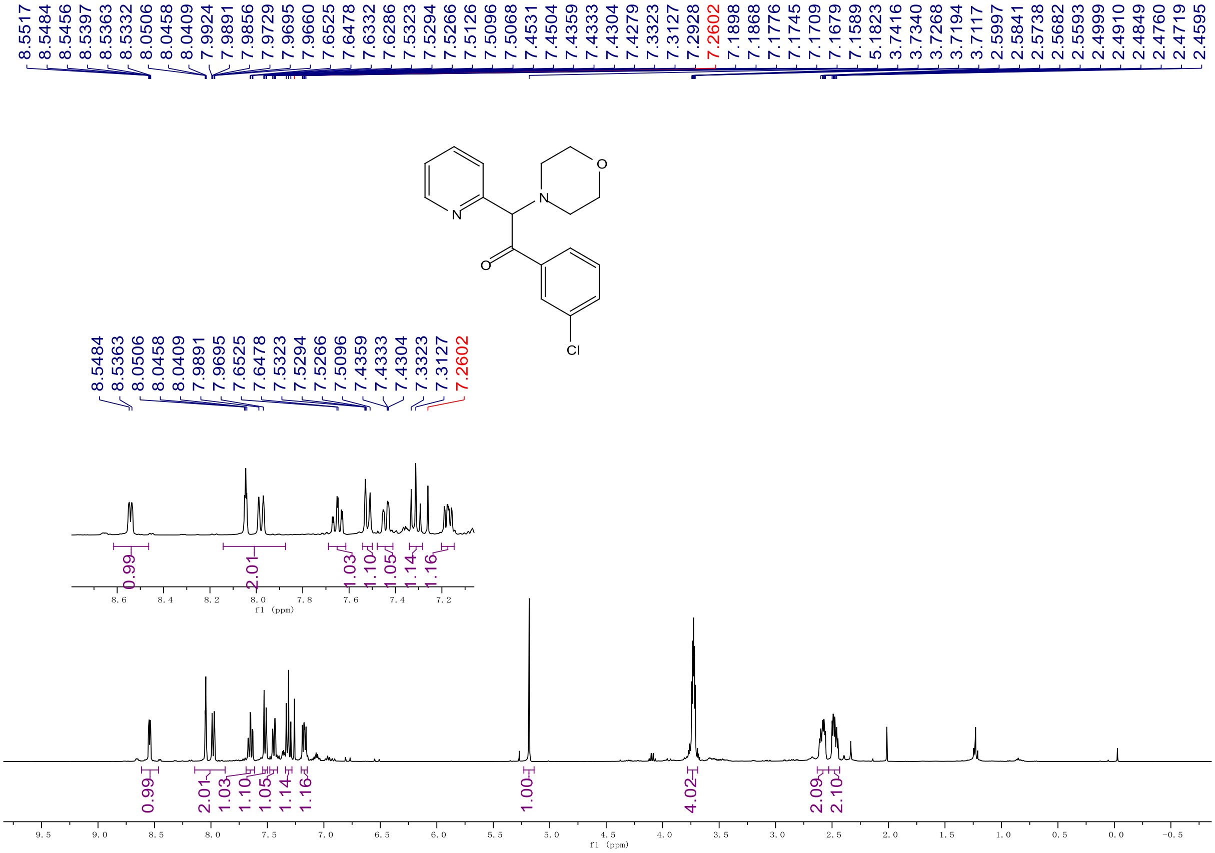

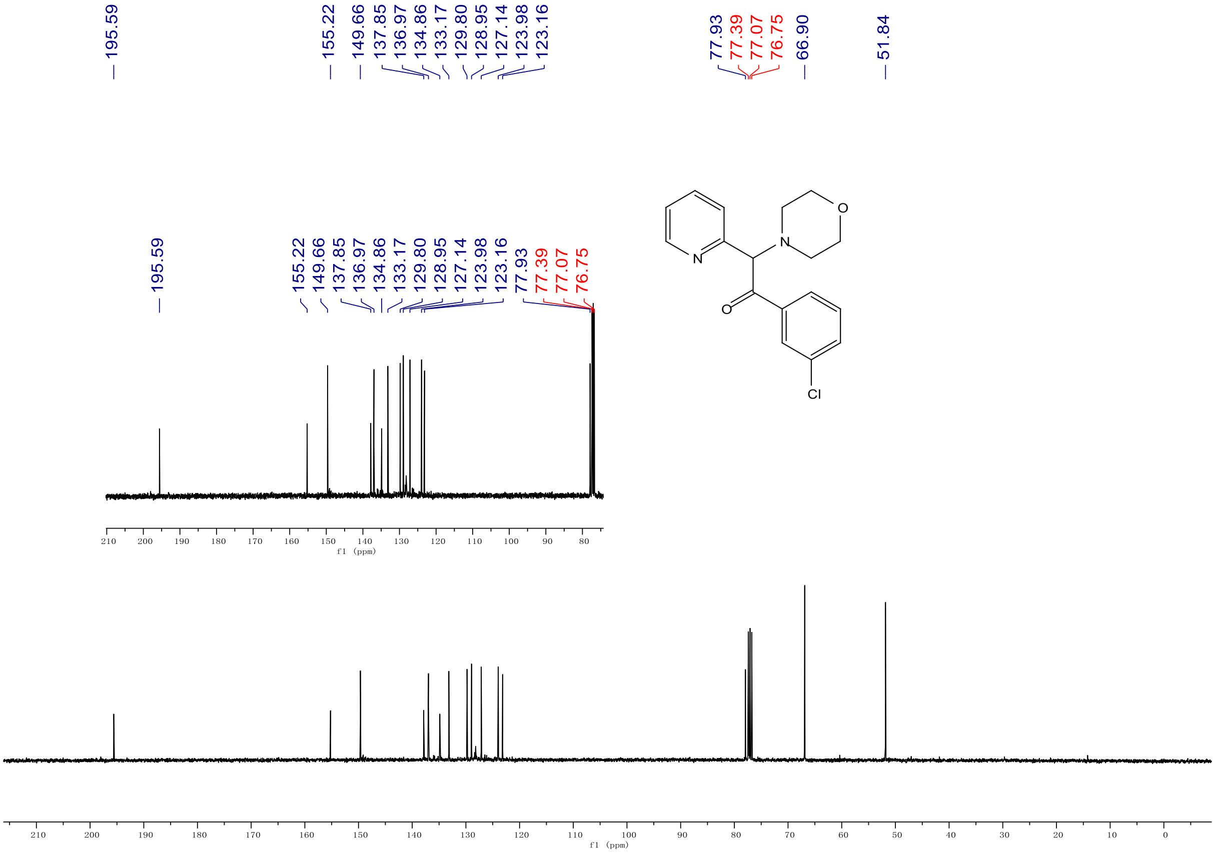

^{1}H, $^{13}C\{^{1}H\}$ and ^{19}F NMR spectra of compound **3an** in $CDCl_3$

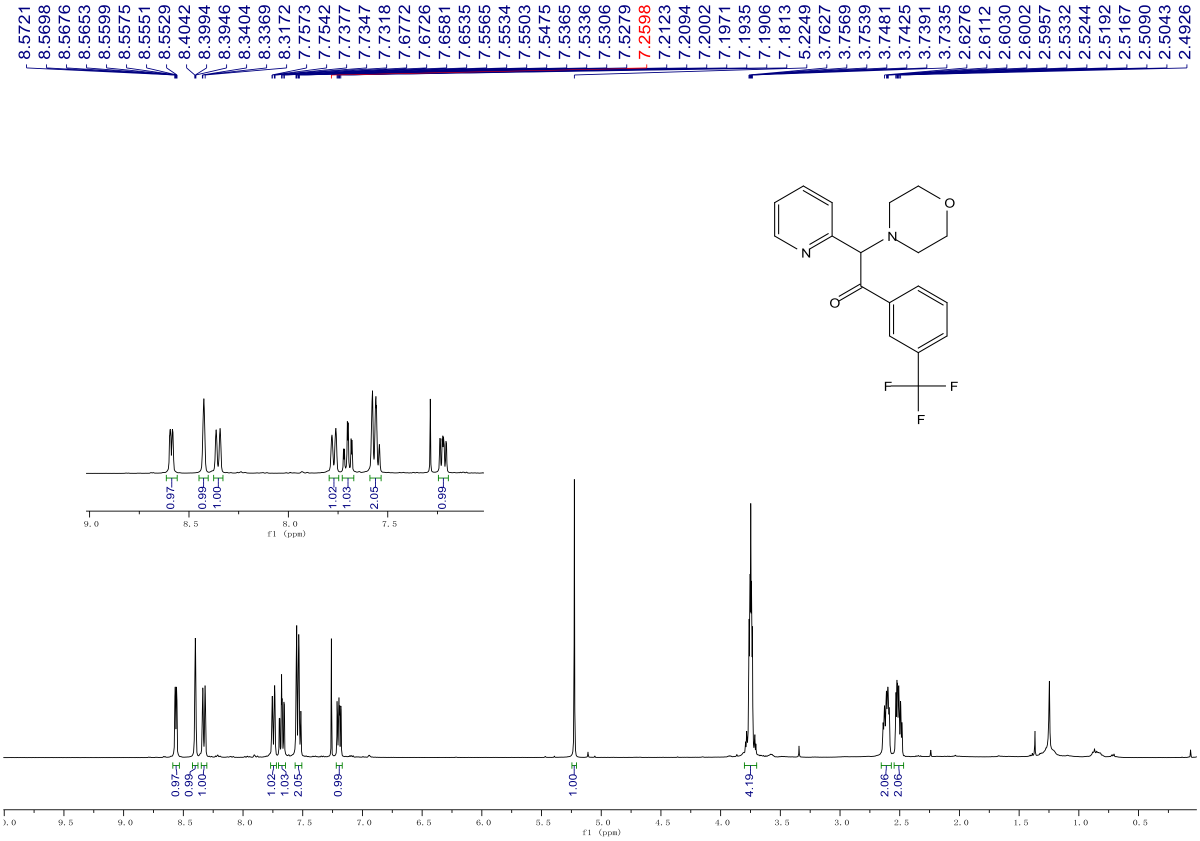

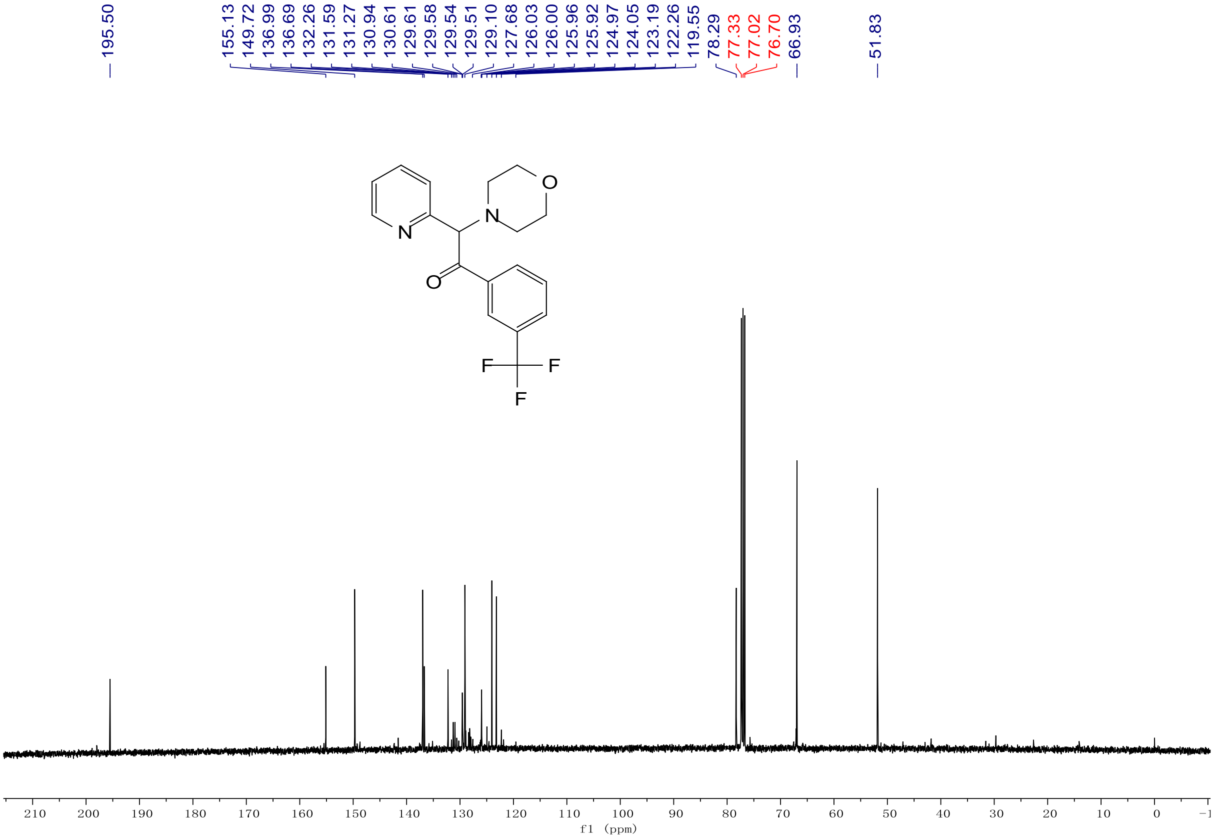

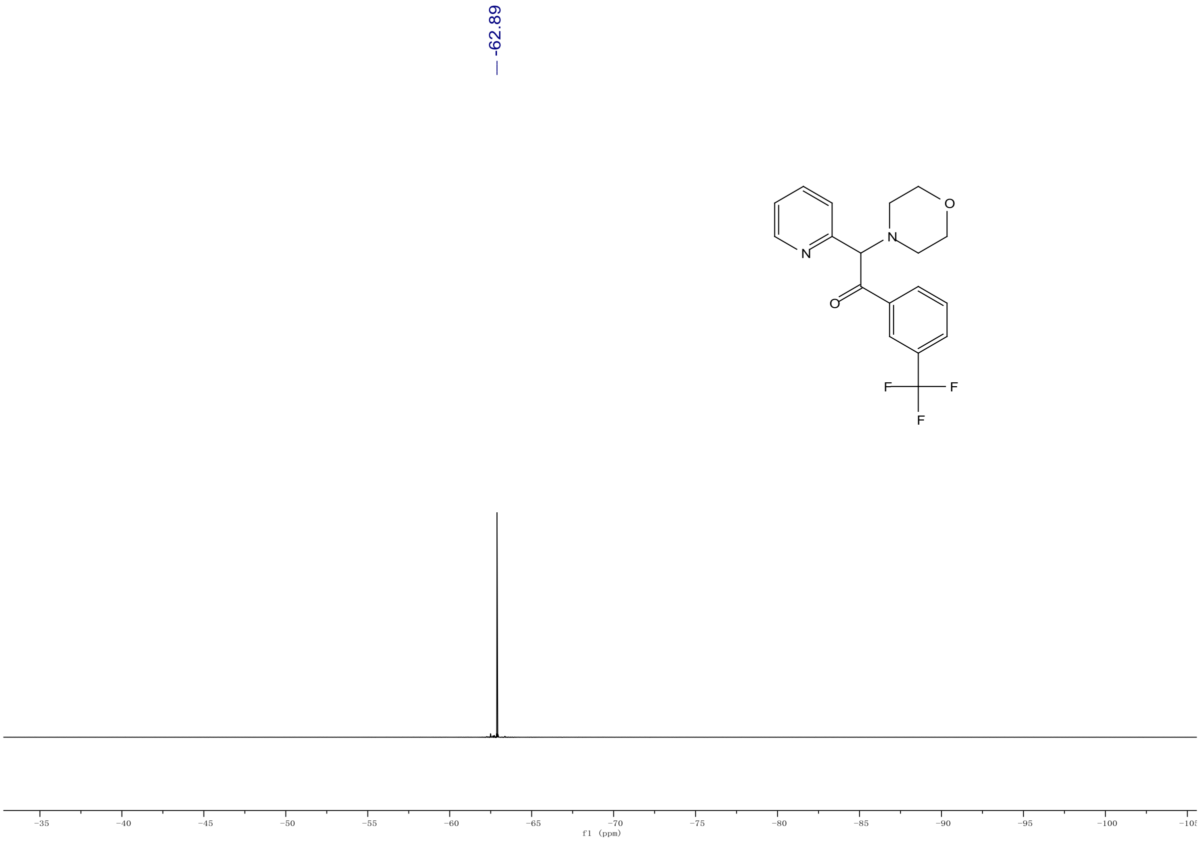

-62.89
F
F
F
N
N
O
O
-35
-40
-45
-50
-55
-60
-65
-70
-75
-80
-85
-90
-95
-100
f1 (ppm)

^{1}H and ^{13}C{^{1}H} NMR spectra of compound **3ao** in $CDCl_3$

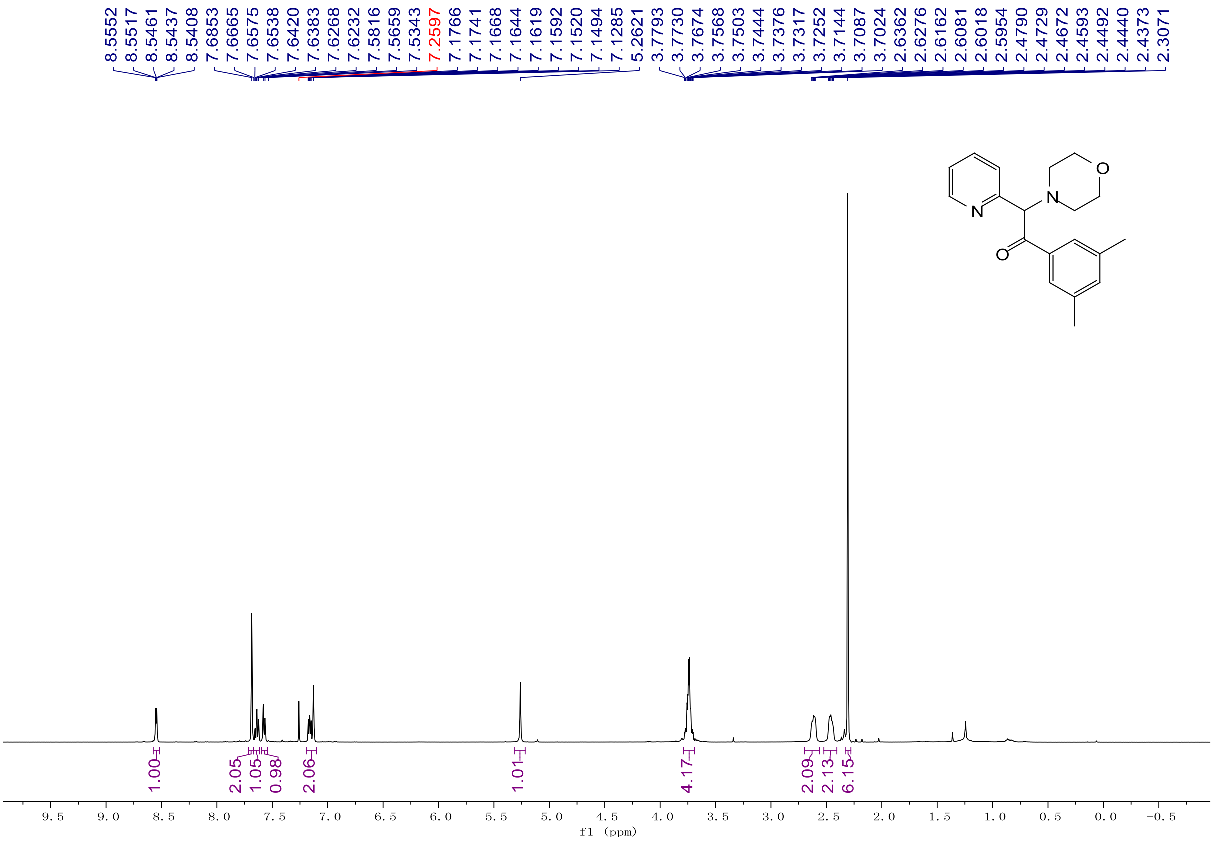

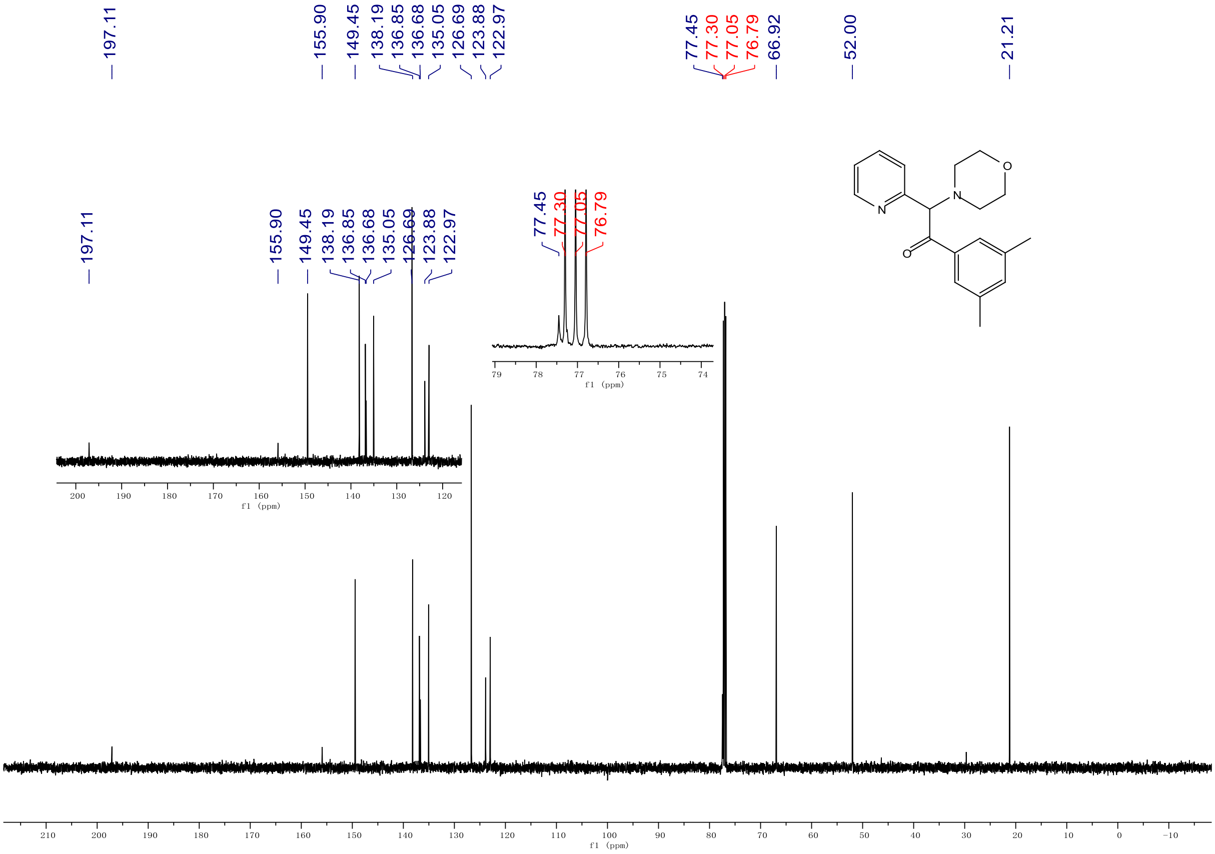

^{1}H and ^{13}C{^{1}H} NMR spectra of compound **3ap** in $CDCl_3$

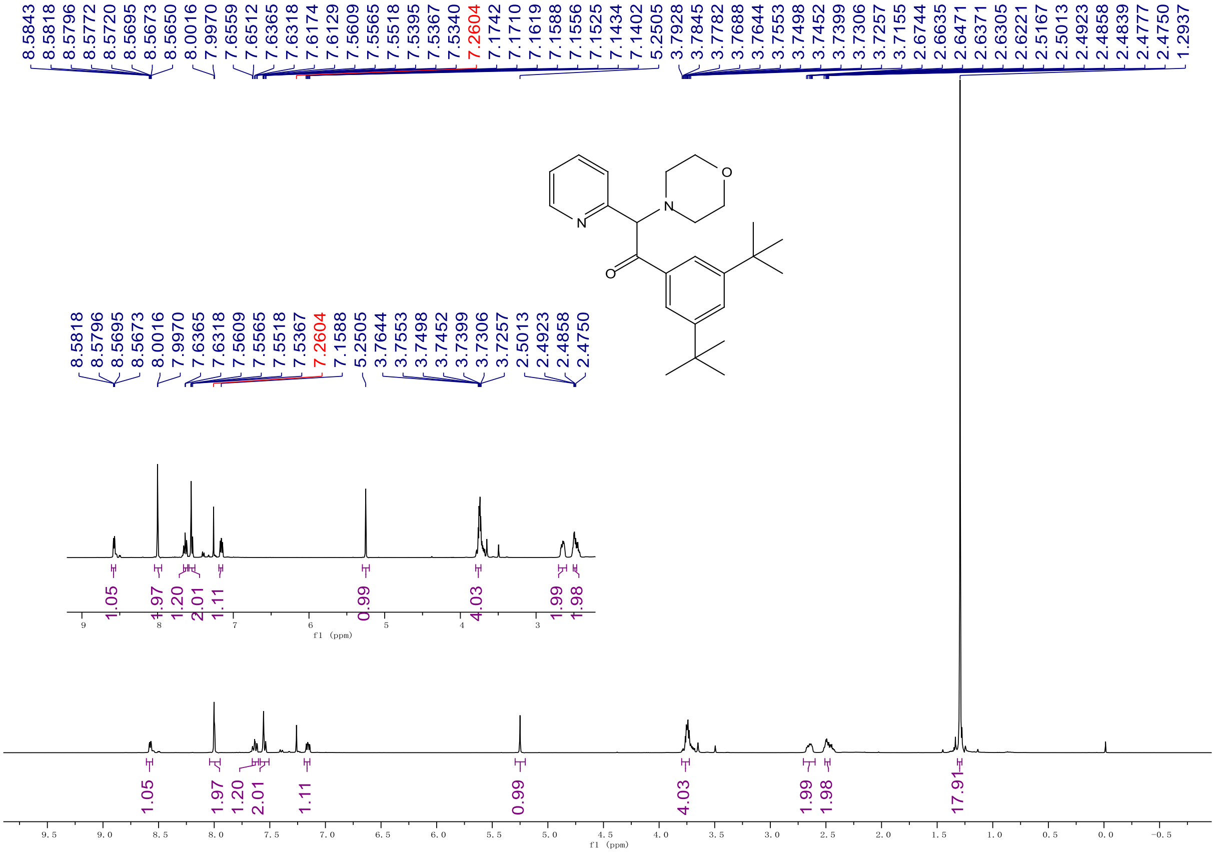

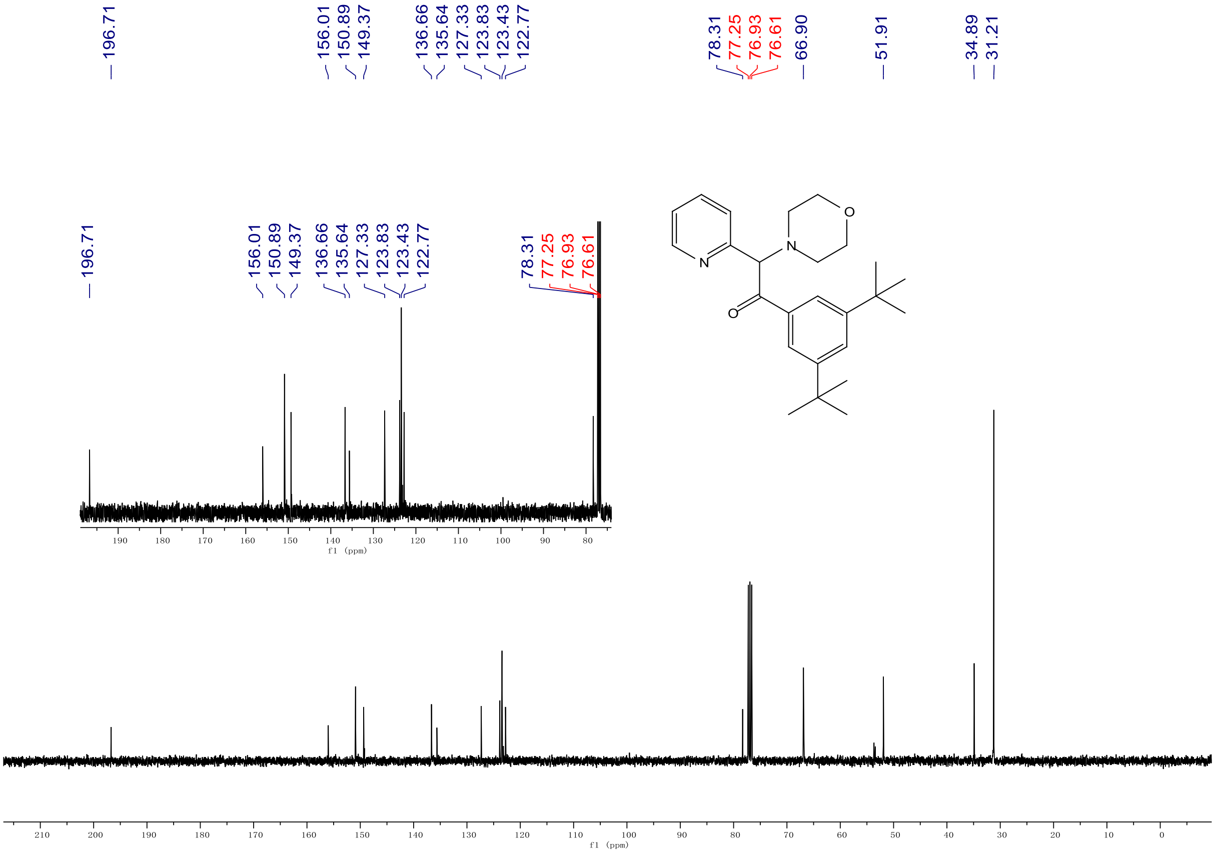

^{1}H and ^{13}C{^{1}H} NMR spectra of compound **3aq** in $CDCl_3$

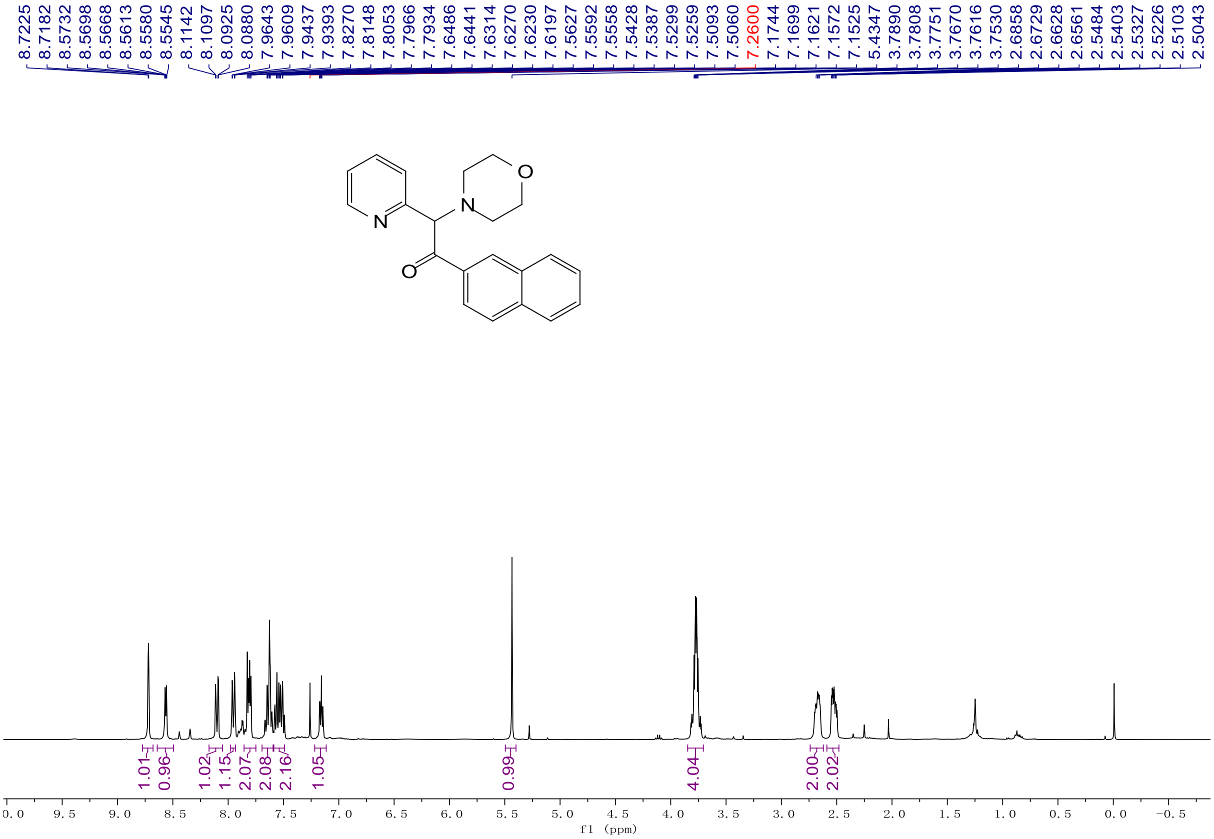

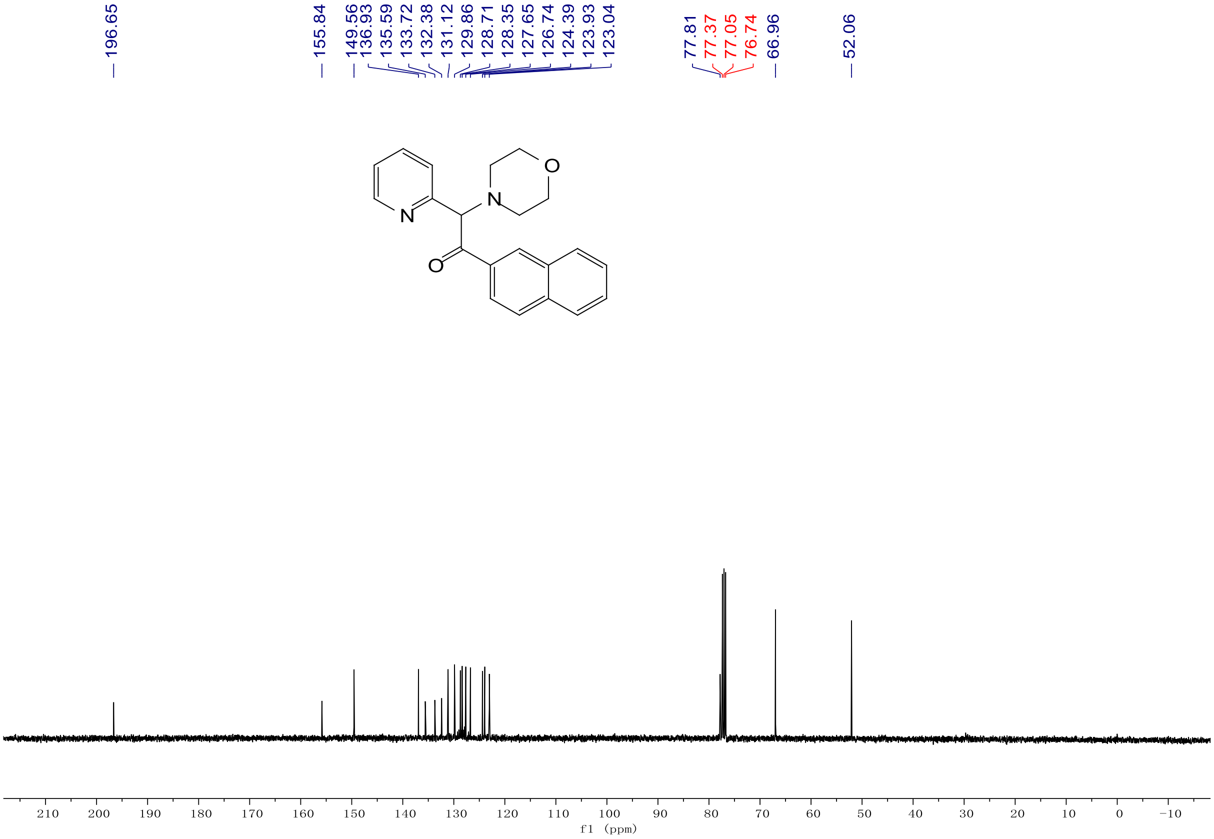

^{1}H and ^{13}C{^{1}H} NMR spectra of compound **3ar** in $CDCl_3$

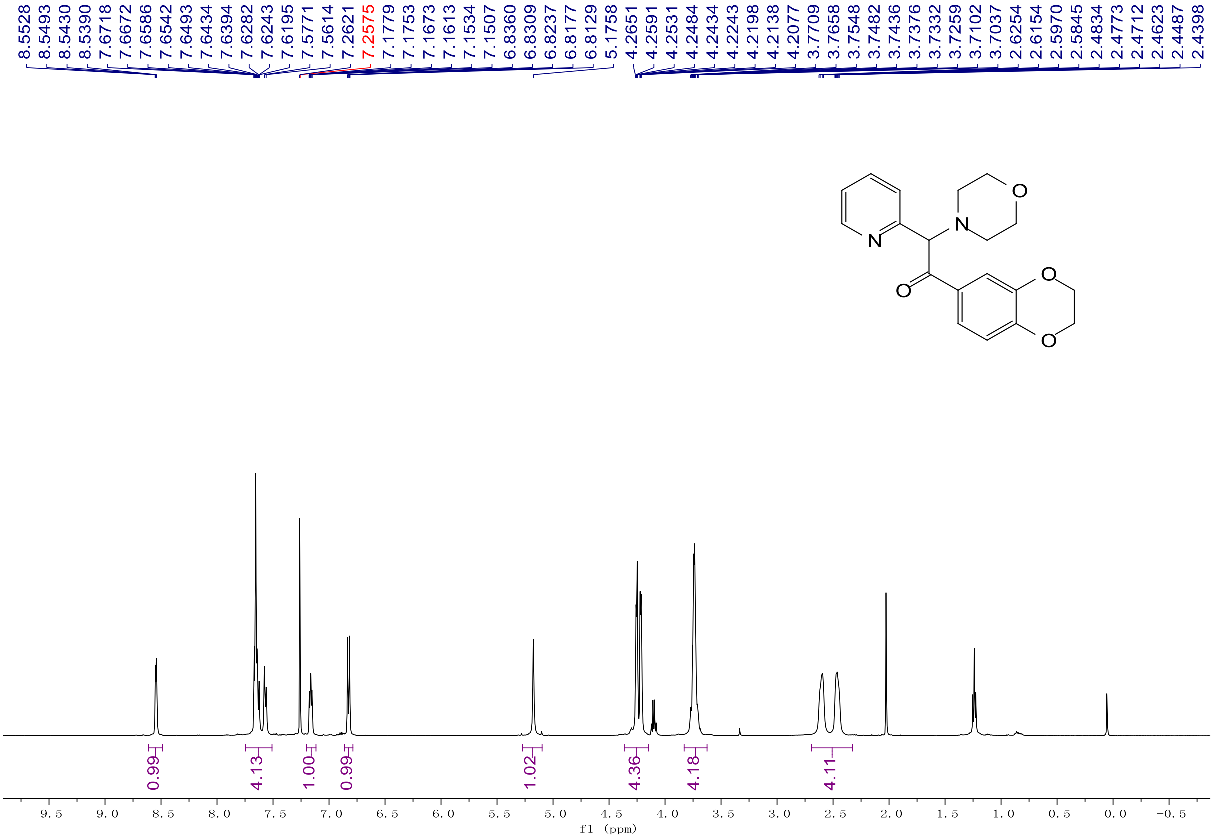

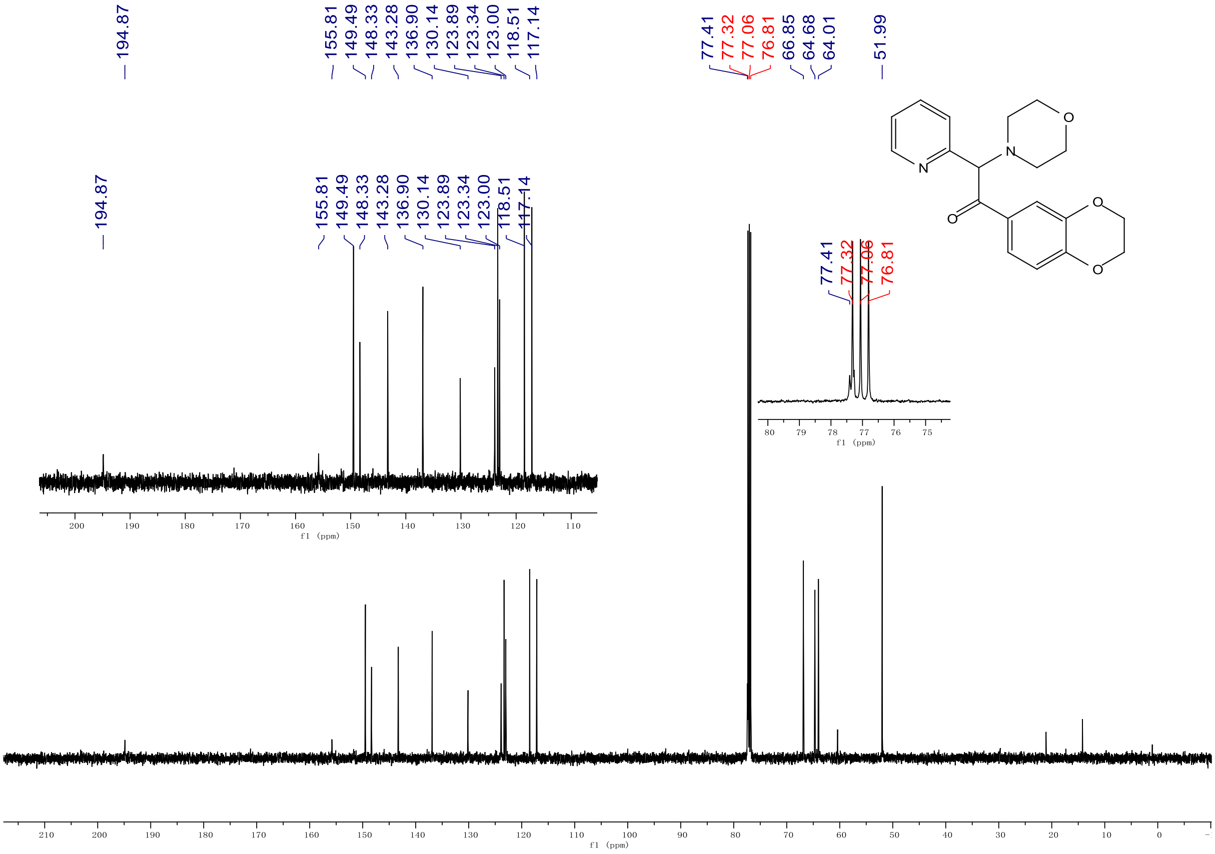

^{1}H and $^{13}C\{^{1}H\}$ NMR spectra of compound **3as** in $CDCl_3$

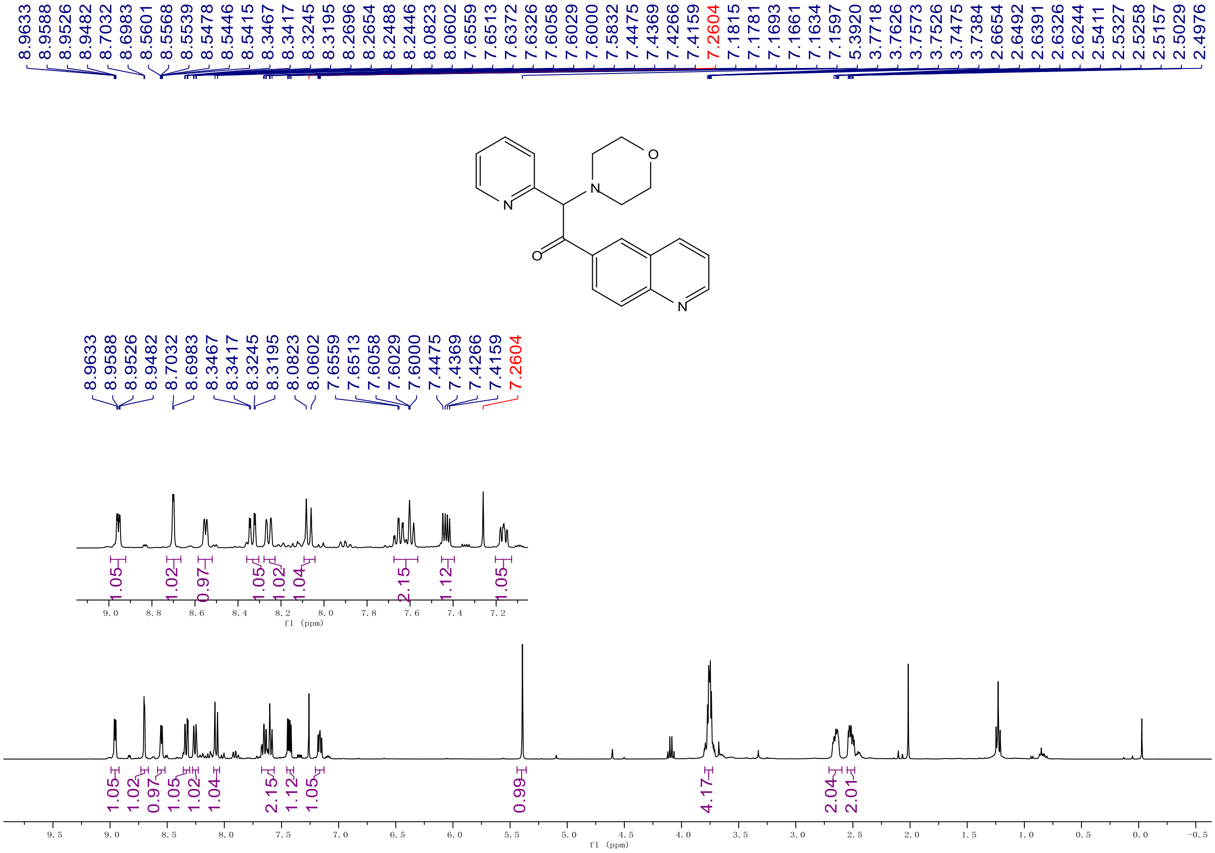

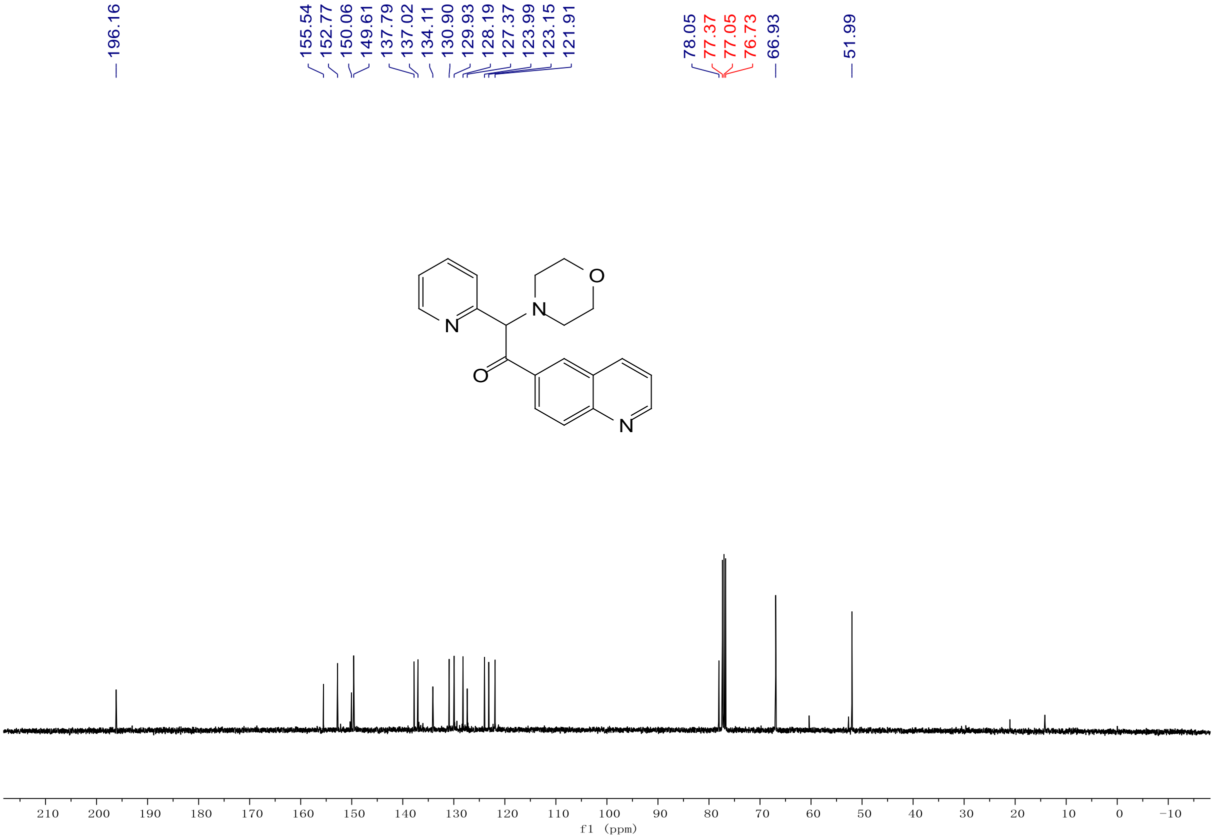

^{1}H and $^{13}C\{^{1}H\}$ NMR spectra of compound **3at** in $CDCl_3$

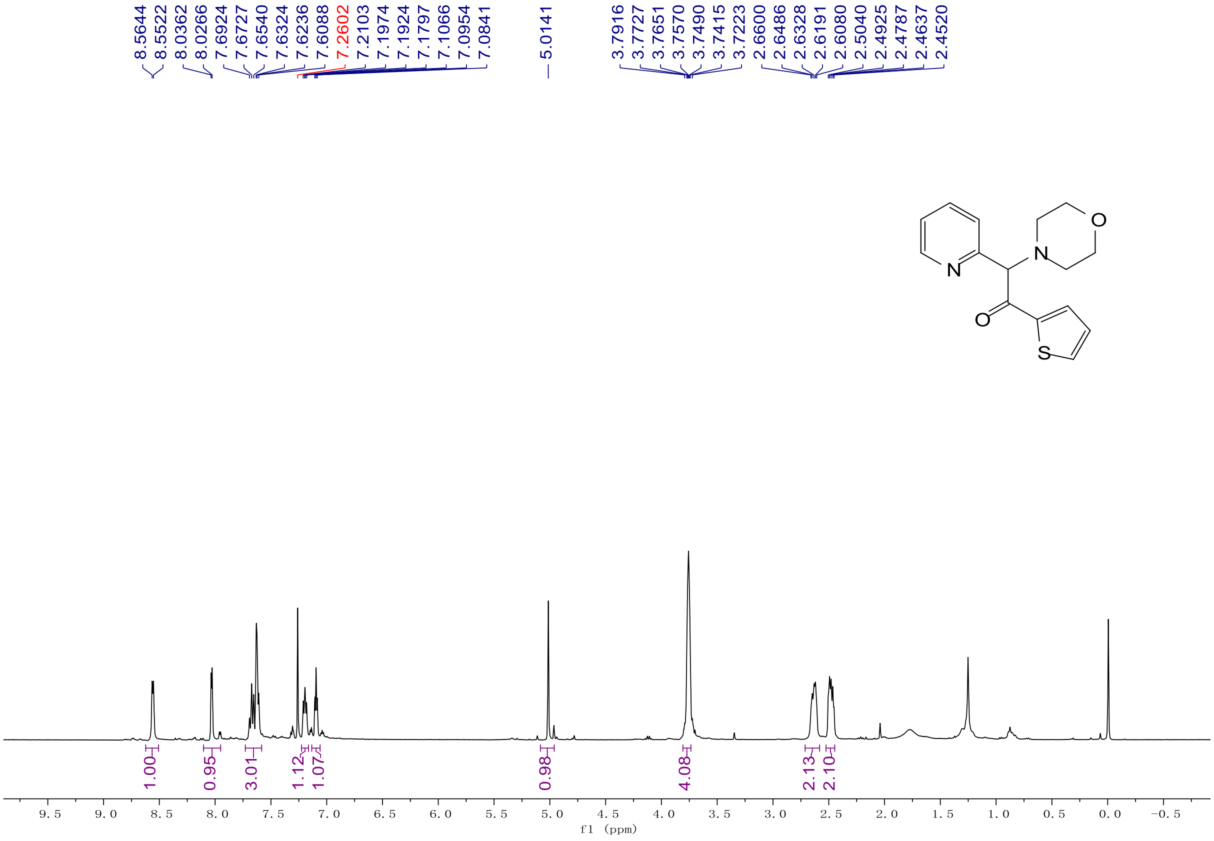

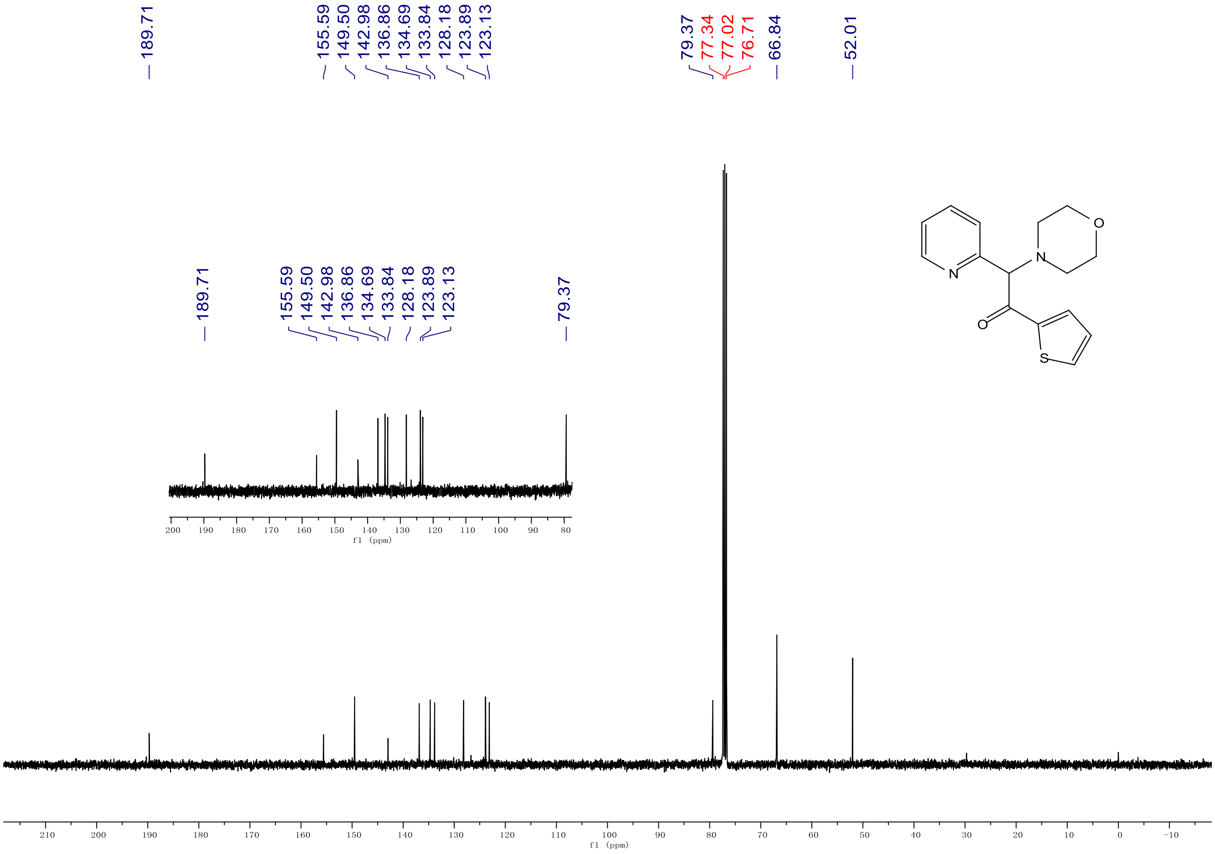

^{1}H and $^{13}C\{^{1}H\}$ NMR spectra of compound **4** in $CDCl_3$

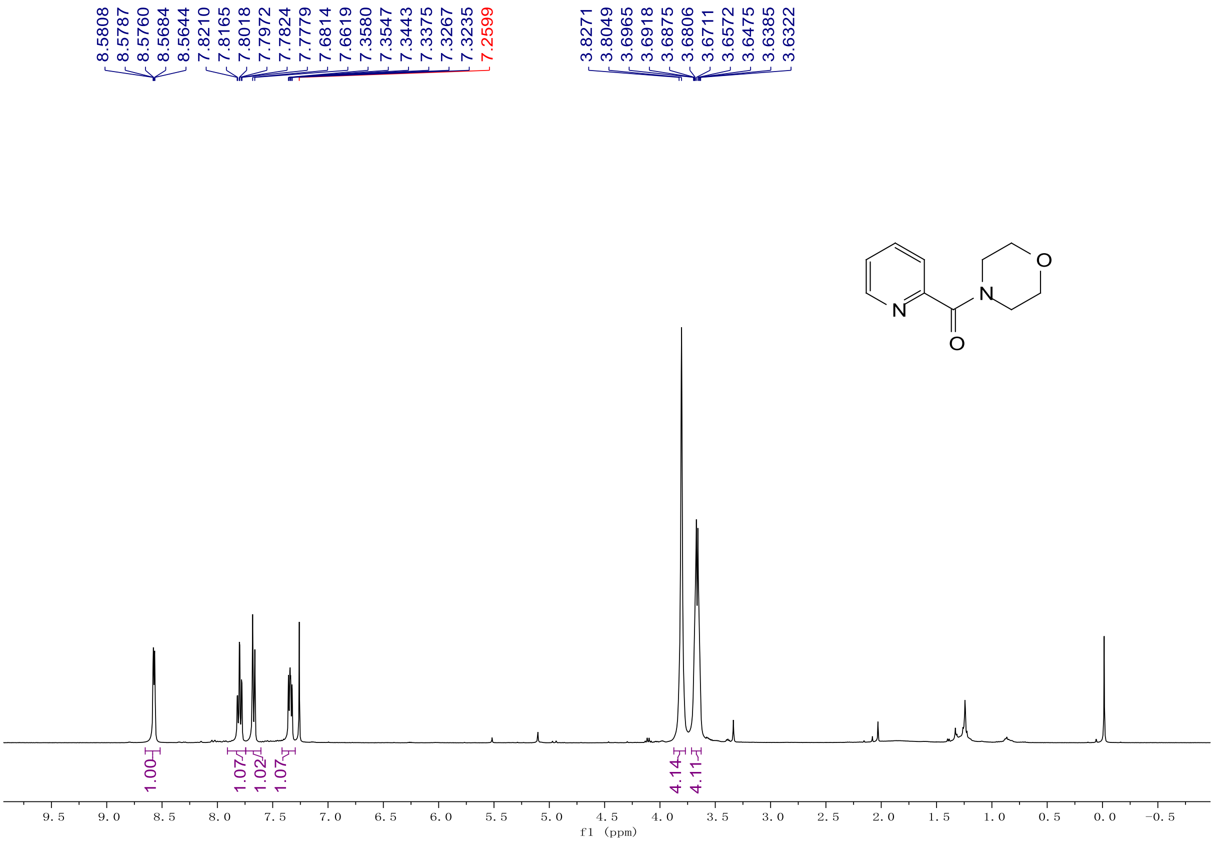

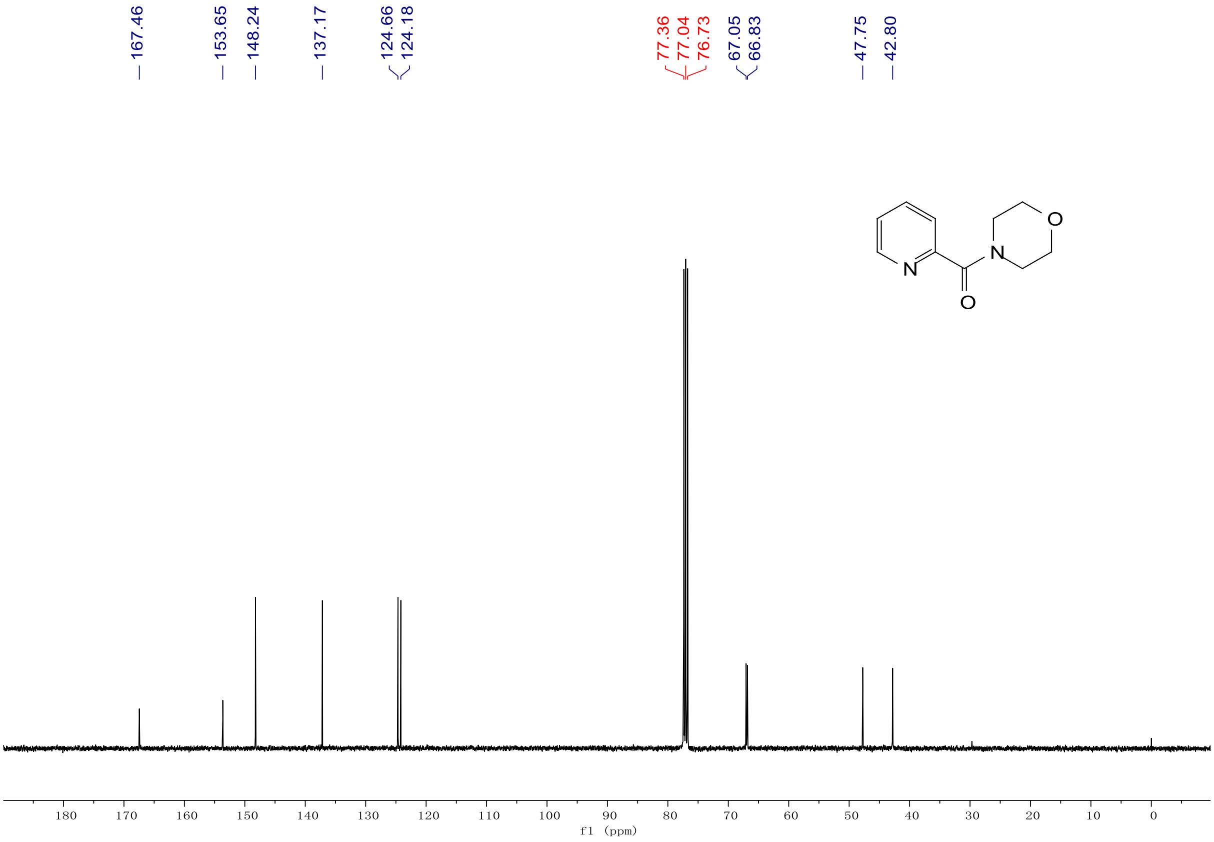

^{1}H and ^{13}C{^{1}H} NMR spectra of compound **5** in $CDCl_3$

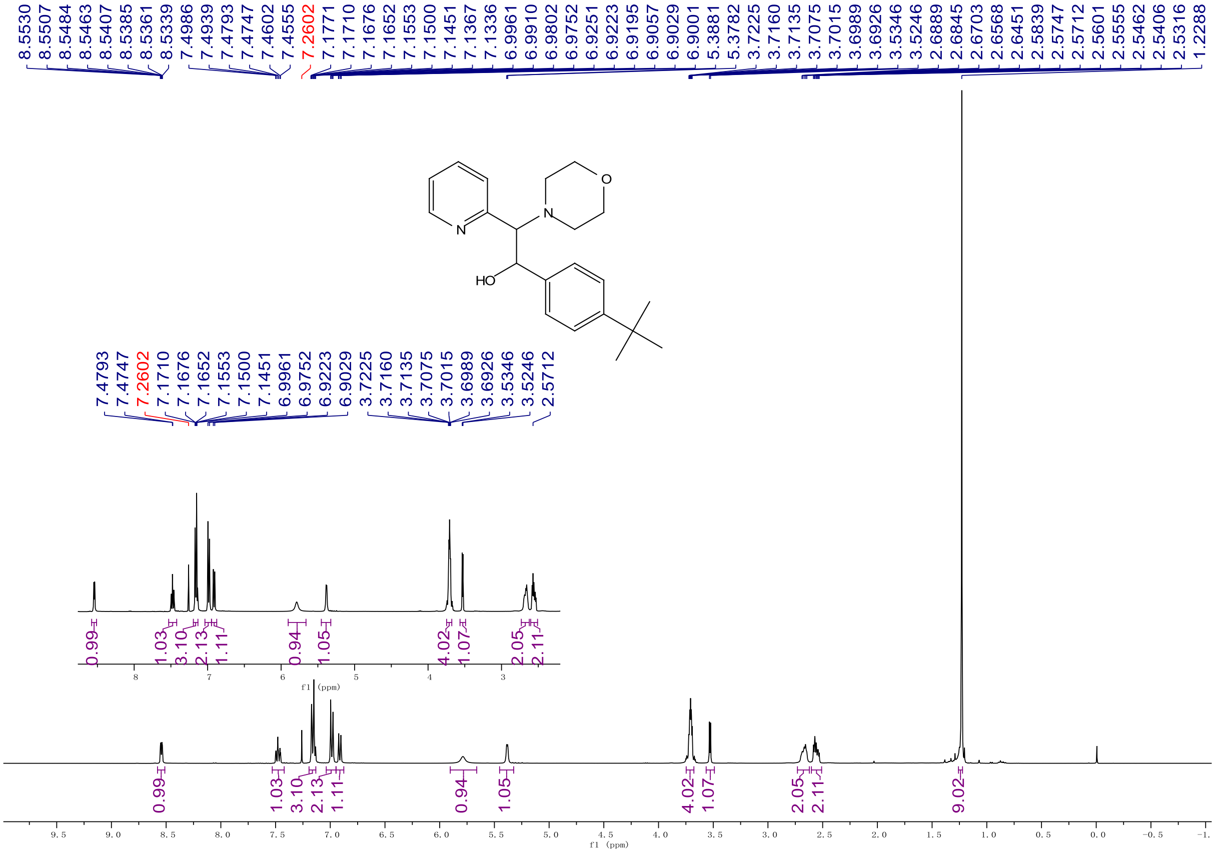

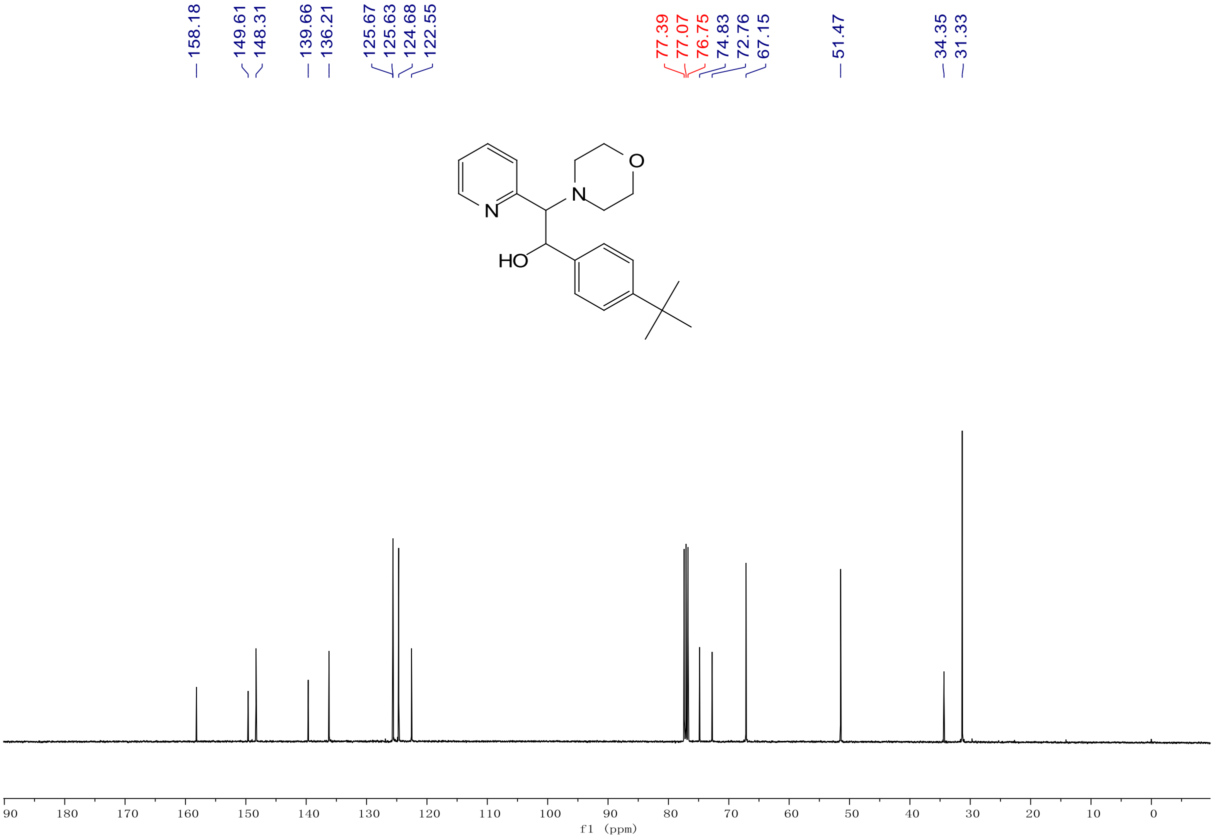

^{1}H and $^{13}C\{^{1}H\}$ NMR spectra of compound **6** in $CDCl_3$

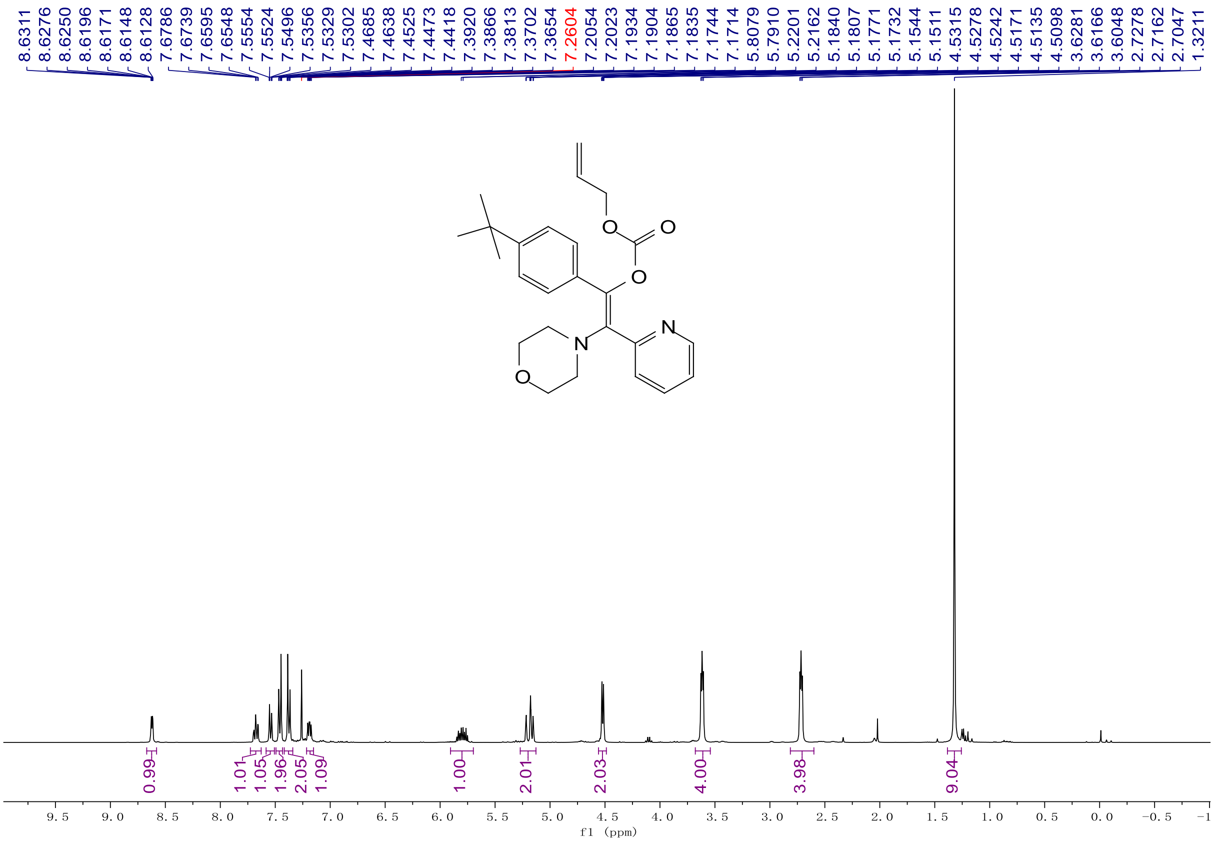

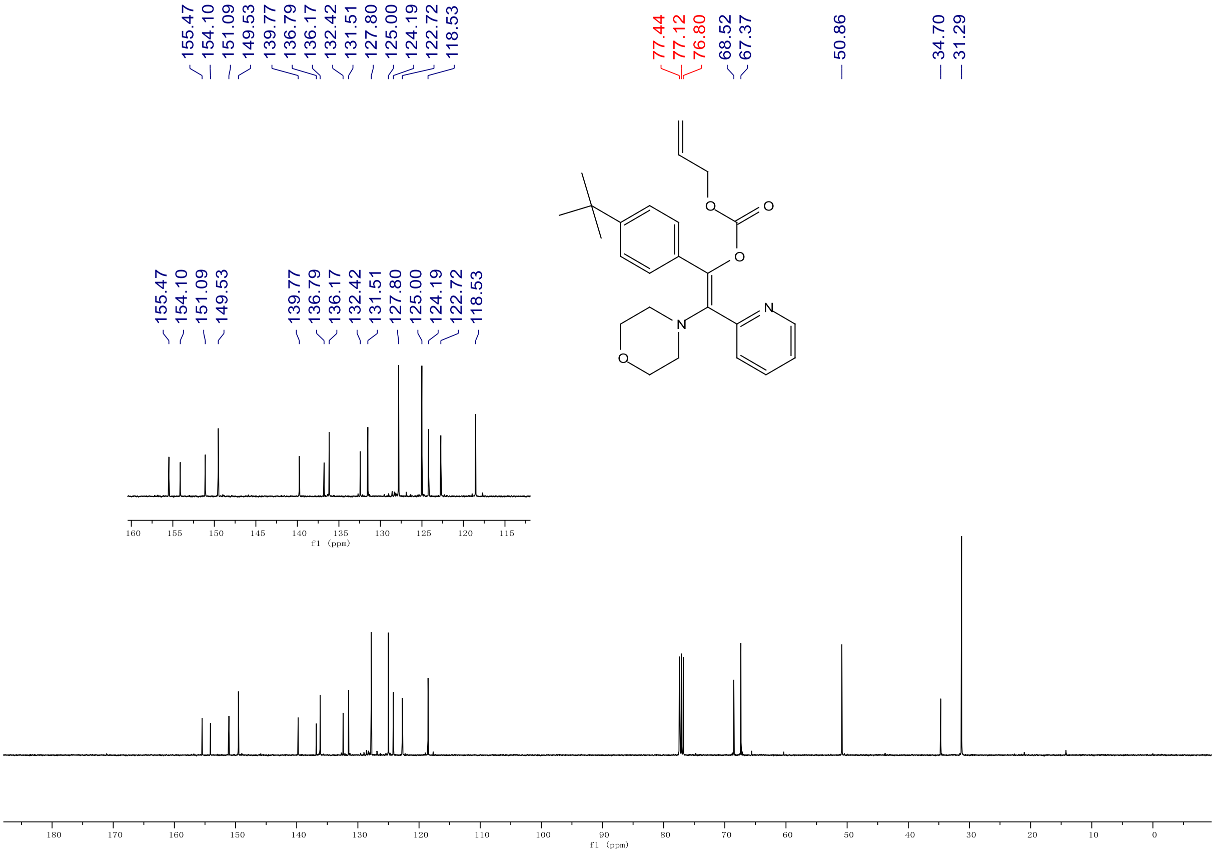

1D-NOESY ^{1}H NMR spectra of compound **6** in $CDCl_3$

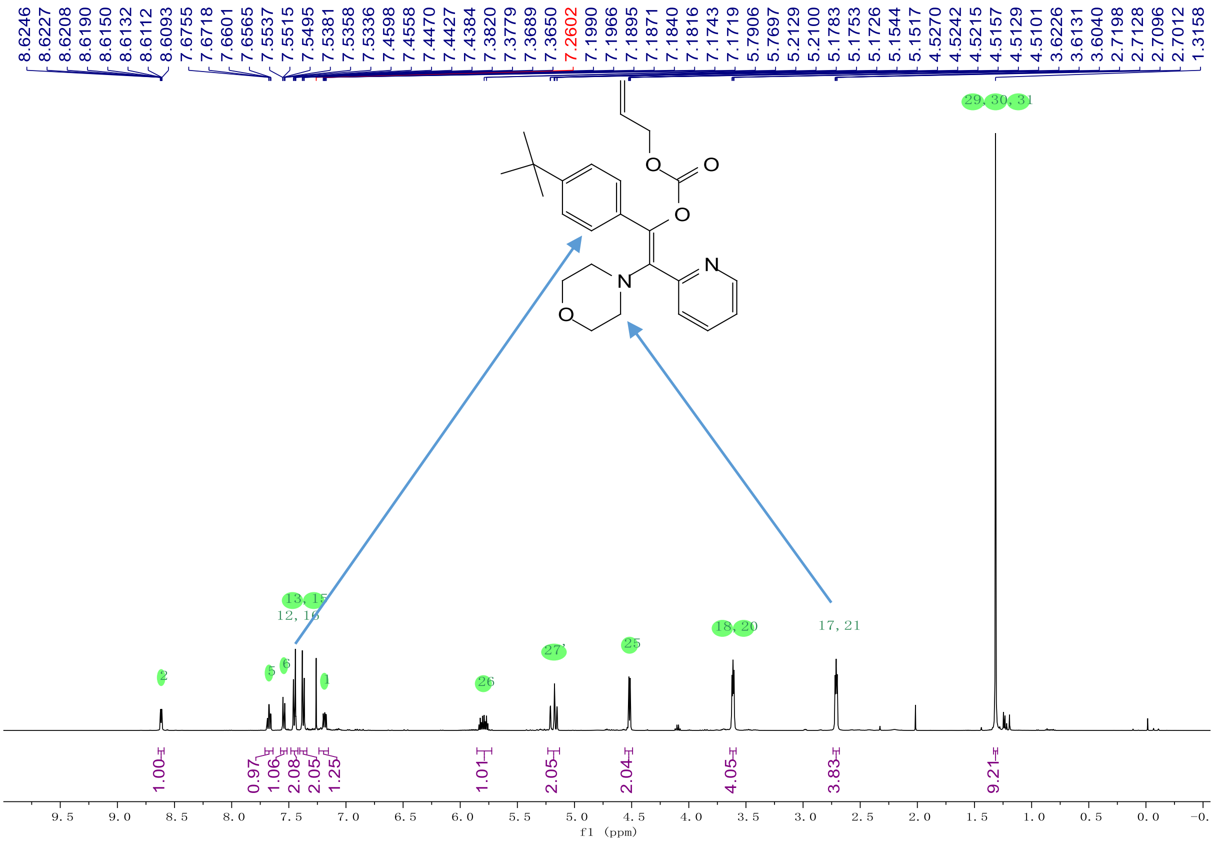

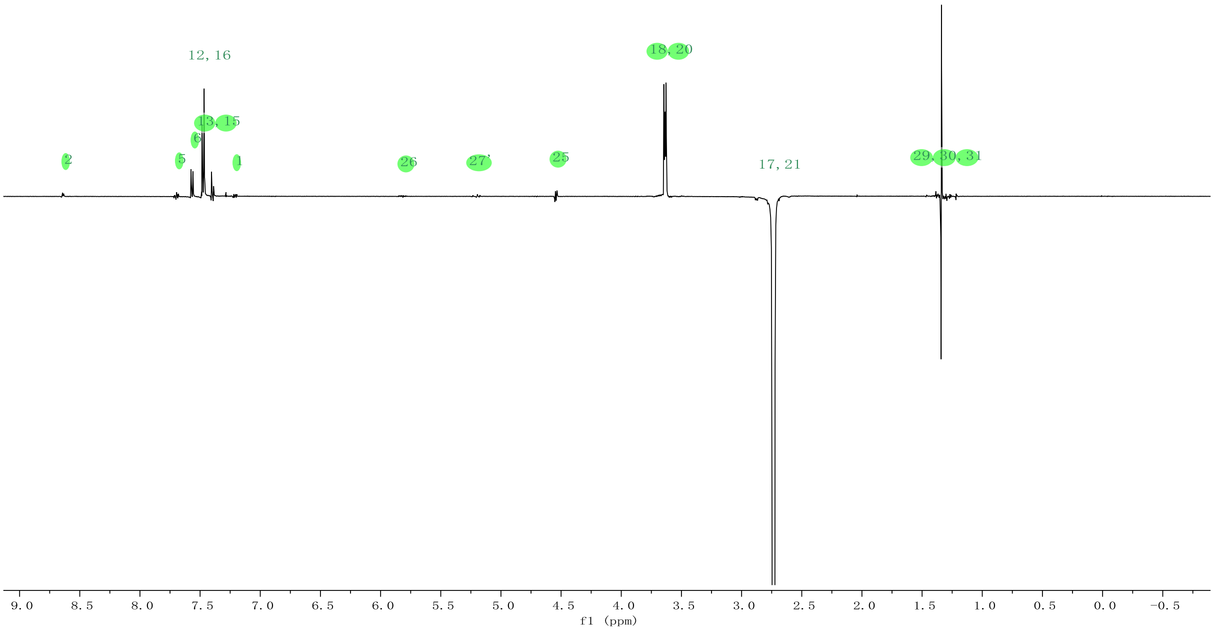

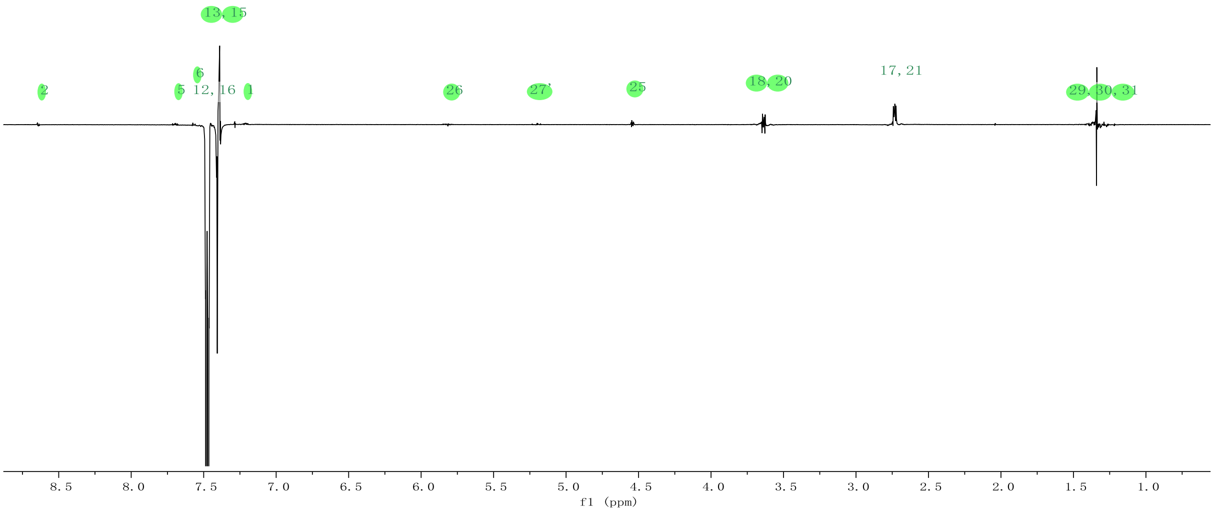
13, 15
6
2
5 12, 16
1
26
27
25
18, 20
17, 21
29, 30, 31
8.5
8.0
7.5
7.0
6.5
6.0
5.5
5.0
4.5
4.0
3.5
3.0
2.5
2.0
1.5
1.0
f1 (ppm)
